# Discovery of 2-deoxy glucose surfaced mixed layer dendrimer: a smart neuron targeted systemic drug delivery system for brain diseases

**DOI:** 10.7150/thno.95476

**Published:** 2024-05-19

**Authors:** Anubhav Dhull, Zhi Zhang, Rishi Sharma, Aqib Iqbal Dar, Anu Rani, Jing Wei, Shamila Gopalakrishnan, Amanda Ghannam, Victoria Hahn, Anunay James Pulukuri, Stefanie Tasevski, Sara Moughni, Boyang Jason Wu, Anjali Sharma

**Affiliations:** 1 Department of Chemistry, College of Arts and Sciences, Washington State University, 1470 NE College Ave, Pullman, WA, USA 99164.; 2 Department of Natural Sciences, College of Arts, Sciences, and Letters, University of Michigan -Dearborn, 4901 Evergreen Rd, Dearborn, MI, USA 48128.; 3 Department of Pharmaceutical Sciences, College of Pharmacy and Pharmaceutical Sciences, Washington State University, Spokane, WA, USA 99202.

**Keywords:** neuron targeting, non-invasive brain targeting, nanotherapeutics, dendrimer, drug conjugate, targeted drug delivery

## Abstract

The availability of non-invasive drug delivery systems capable of efficiently transporting bioactive molecules across the blood-brain barrier to specific cells at the injury site in the brain is currently limited. Delivering drugs to neurons presents an even more formidable challenge due to their lower numbers and less phagocytic nature compared to other brain cells. Additionally, the diverse types of neurons, each performing specific functions, necessitate precise targeting of those implicated in the disease. Moreover, the complex synthetic design of drug delivery systems often hinders their clinical translation. The production of nanomaterials at an industrial scale with high reproducibility and purity is particularly challenging. However, overcoming this challenge is possible by designing nanomaterials through a straightforward, facile, and easily reproducible synthetic process.

**Methods:** In this study, we have developed a third-generation 2-deoxy-glucose functionalized mixed layer dendrimer (*2DG-D*) utilizing biocompatible and cost-effective materials *via* a highly facile convergent approach, employing copper-catalyzed click chemistry. We further evaluated the systemic neuronal targeting and biodistribution of *2DG-D*, and brain delivery of a neuroprotective agent pioglitazone (*Pio*) in a pediatric traumatic brain injury (TBI) model.

**Results:** The *2DG-D* exhibits favorable characteristics including high water solubility, biocompatibility, biological stability, nanoscale size, and a substantial number of end groups suitable for drug conjugation. Upon systemic administration in a pediatric mouse model of traumatic brain injury (TBI), the *2DG-D* localizes in neurons at the injured brain site, clears rapidly from off-target locations, effectively delivers *Pio*, ameliorates neuroinflammation, and improves behavioral outcomes.

**Conclusions:** The promising *in vivo* results coupled with a convenient synthetic approach for the construction of *2DG-D* makes it a potential nanoplatform for addressing brain diseases.

## Introduction

Delivering therapeutic molecules across the blood-brain barrier (BBB) has long been the most prominent obstacle in the treatment of brain disorders 1. Roughly 98% of drugs identified through high-throughput screening fail to advance to the next phase of drug development because they cannot effectively penetrate this protective barrier. Due to this reason, there is a greater incidence of failures during the later stages of CNS drug development as compared to non-CNS drugs. Consequently, there has been a reduced enthusiasm in the pharmaceutical industry for creating new CNS medications 2. However, there has been a simultaneous rise in the academic CNS drug discovery 3. Lately, nanomaterials have become indispensable in the diagnosis and targeted treatment of various unmet clinical needs including brain diseases. Nanoparticles not only improve drug pharmacokinetics and biodistribution, but also offer controlled release kinetics at the intended target site by navigating through various biological barriers effectively 4, 5 Efforts have persistently been underway to develop novel nanocarriers which are capable of precisely transporting drugs across the BBB to target the specific regions of brain damage 6-8. However, there is a limited presence of nanocarriers in the literature which are capable of specifically delivering therapies to neurons at the site of brain injury from non-invasive systemic administration routes 9, 10. Even if drugs or nanoparticles get across the impaired BBB following brain injury or neuroinflammation, their uptake into the critical brain cells such as neurons, involved in brain diseases remains challenging 11. Targeting neurons is specifically more complex since they are far lower in number and less phagocytic in nature compared to the other immune cells in the brain 12. Moreover, the brain comprises various neuronal subtypes, each serving distinct functions, making it crucial to pinpoint those relevant to the specific disease 13, 14.

Traumatic brain injury (TBI) stands as a prominent global contributor to fatalities and impacts countless individuals, with even more dire consequences in low and middle-income countries 15, 16. Survivors endure long term disabilities, compromised neurological function, shifts in behavior, depression and require extensive long-term rehabilitation 17. The pathology of TBI is complicated and involves a primary insult due to direct physical trauma to the brain, which in turn leads to a secondary insult, such as neuroinflammation, oxidative stress and excitotoxicity, caused by destructive biochemical cascades ultimately leading to the death of glia and neurons 18, 19. Microglia and astrocytes are activated after encountering TBI, which leads to the overproduction of neuroinflammatory mediators that intensify TBI, resulting in neuronal damage 20. Although huge research advancements have been made in the field of TBI, there is no approved therapy available to mitigate long term outcomes. Numerous potential treatments have faced challenges in late-stage clinical trials because they didn't achieve the necessary drug levels at the specific disease site. These drug delivery obstacles can be surmounted by developing innovative and biocompatible nanocarriers that possess enhanced targeting capabilities. Of particular significance is the precise delivery of drugs to vital cells like neurons at the site of brain injury.

Within the realm of polymer-based nanoparticulate drug delivery systems, dendrimers, which are hyper-branched, uniformly sized, monodispersed synthetic macromolecules with precisely defined structure and composition, have gained extensive utilization in the field of drug delivery systems 21, 22. This arises from the meticulous control over their properties like molecular structure, size, shape and solubility. Dendrimers also offer the prospect of attaching targeting agents, imaging dyes, small molecule therapeutics and biologics to their multi-valence surface groups, enabling precise targeting, imaging, and therapeutic interventions for various diseases 23-25. Notwithstanding these benefits, the successful translation of different dendrimer-based drug delivery systems to clinical applications remains infrequent 26. Beyond the obstacle of attaining precise target specificity, there are additional challenges in the clinical implementation and commercialization of dendrimer-based drug delivery, including concerns related to cytotoxicity, scalability, structural imperfections, complex synthetic design, consistency in production, product purity, and *in vivo* stability. These hurdles can be circumvented by creating simple design, incorporating biocompatible and non-toxic building blocks, utilizing inexpensive starting materials, incorporating non-cleavable linkages within the structure, and employing highly efficient and easily scalable chemical transformations.

In context of dendrimers mediated intracellular drug delivery to brain, hydroxyl terminating polyamidoamine (PAMAM-OH) dendrimers have shown tremendous potential for the treatment of central nervous system disorders and are currently undergoing clinical translation 27, 28. Systemically administered PAMAM-OH dendrimers have shown to cross the impaired BBB in multiple animal models of neuroinflammation, and target activated microglia and macrophages at the site of injury in the brain 24, 29. This precise targeting has translated to significant efficacies for PAMAM-OH-drug conjugates in comparison to the free drugs, clearly demonstrating the effect of delivering therapeutics to the affected cells at the site of pathology in the brain 30-32.

We here report the rational design and synthesis of the 2-deoxy glucose (*2DG*) surfaced mixed-layer dendrimer (*2DG-D*) for targeted delivery of drugs to neurons at the site of injury in the brain. Among a few rare reports on neuron-targeted systemic nanocarriers, glucosylated polymeric micelles have been reported to be distributed in the brain with specific localization in neurons 33. We incorporated *2DG* at the surface of *2DG-D* to achieve neuron targeting *via* GLUT transporters. To avoid any delivery of 2-deoxyglucose to neurons in injured brain regions which in some neurological conditions can have deleterious effects, we utilized robust chemical linkages within the dendrimer backbone, preventing degradation, and enabling efficient clearance (4.3 nm in size) from non-target organs, primarily through renal excretion. We further demonstrated the *in vitro* and *in vivo* neuronal targeting of *2DG-D* and brain delivery of a neuroprotective agent pioglitazone (*Pio*) in a pediatric mouse model of TBI. *Pio* is an anti-diabetic drug being widely investigated for brain diseases, including TBI 34-38. However, its poor aqueous solubility and peripheral side-effects are a concern that can be addressed using *2DG-D* mediated targeted delivery 39-41. Collectively, this strategy has been rationally crafted to facilitate precise targeting of neurons at the affected brain site, enhancing the delivery of therapeutic agents for the treatment of TBI and various other neurological disorders.

## Methods

### Chemistry experimental section

Experimental methods related to materials, instrumentation, synthesis protocols, *in vitro* drug release studies, and *2DG-D-Pio* formulation stability studies are presented in supplementary information.

#### Materials and reagents

PHEMS buffer, PBS, Poly-L-lysine, 4′,6-diamidino-2-phenylindole (DAPI), 3-(4,5-dimethylthiazol-2-yl)-2,5 diphenyltetrazolium bromide (MTT), and Triton X-100 were procured from Aaron Chemicals. The cell painting kit and PhenoVue neuronal differentiation staining kit were purchased from Revvity. CATH.a cell line was obtained from ATCC, United States. DMEM, RPMI-1640 was purchased from Cytiva, FBS was obtained from Gibco Scientific and DAPI-cell mounting medium was obtained from Vectorlabs. Rat red blood cells (RBCs) were purchased from Innovative Research. All these above-mentioned reagents were used as such.

### *In vitro* studies experimental section

***Primary culture of the cortical neurons:*** The cortical neurons were isolated as per the described procedure 42, 43. Briefly, the brain tissue from the Sprague Dawley rats was collected at post-natal day 0. The dorsal and frontal regions of the cortex were then dissected and incubated in the buffer for digestion. The buffer contained 10 unit/mL papain, 100 unit/mL DNase I, and 5 mg/mL cystine, in Hibernate A nutrient medium. After 30 min of the digestion, the neurons were further seeded on poly-D-lysine (50 µg/mL)-coated plates (1.5×10^5^) in a 24 well plate. For culturing of the cortical cells, the medium was Neurobasal A with B27 supplement with 1% anti-anti and glutamate. The cells were then directly used for further experiments.

***Cellular compatibility/MTT assay using primary and secondary neuronal cultures:*** For evaluating the applicability of the developed *2DG-D* dendrimers for *in vivo* studies, these were first evaluated under *in vitro* settings with both cortical and CATH.a neuronal cells. In this assay, briefly 1×10^5^ cortical neuron cells and 1×10^4^ CATH.a neurons were plated in a 24 well and 96 well plate, respectively, in presence of the respective growth media with 10% FBS supplement. Following this, the cells were grown overnight in a CO_2_ incubator with 5% CO_2_ at 37 °C. The media was removed further, and the corresponding cells were treated with different dendrimers and control samples along with the fresh media for 24 and 48 h. Afterwards, the media was removed, and the cells were carefully washed thrice by 1X PBS (10 mM, pH 7.4). Following this, 10 µl MTT (5 mg/mL) was added to each of the wells and incubated for almost 3 h at 37 °C in a CO_2_ incubator. All the media was aspired carefully, and to each well 150 µL DMSO was added and incubated for 15 min for proper dissolution of the formazan aggregates. Finally, the cell viability was calculated by recording the absorbance at 570 nm using multi-mode microplate reader (Thermo Scientific Multiskan SkyHigh Microplate Spectrophotometer). All the samples were done in triplicates with proper controls. The cell viability (%) was calculated by equation (1).



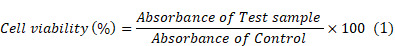



***Cell viability studies using macrophages:*** 2.5 x 10^4^ RAW Blue macrophages were allowed to adhere to a 96 well plate overnight. The following day, cell media was changed and the *2DG-D* dendrimers at the concentrations tested were added. The cells were allowed to incubate at 37 °C for 48 h. Following incubation, the luminescence of the viable cells was measured using the CellTiter-Glo Luminescent Cell Viability assay according to manufacturer's instructions. Cell viability (%) was calculated using the luminescence values obtained from the controls used in the experiment. The experiment was performed in triplicate.

***Cellular internalization/uptake and blocking studies of 2DG-D-Cy5:*** In order to evaluate the uptake of *2DG-D-Cy5* dendrimers by the cells, cellular internalization studies were carried out with both primary isolated cortical neurons and CATH.a neurons. Both these cell lines were employed to increase the mimic for *in vivo* studies for TBI mouse model. First, qualitative uptake studies were carried out only with the primary isolated cortical neurons to optimize the dendrimer concentration for the uptake studies. For this, the isolated cortical neurons were seeded in a 24 well plate having poly -L lysine coated cover glasses with a seeding density of 1×105 cells/well. The cells were grown for 24 h at 37 °C with 5% CO_2_ and 95% moisture, in a CO_2_ incubator. The media was removed after 24 h and the cells were washed with 1X PBS. Different concentrations (100, 50, 25, 12.5, 6.3 and 3.1 µg/mL) of *2DG-D-Cy5* was added to the respective wells with the fresh media and the cells were incubated for 12 h in the CO_2_ incubator. The cells were washed again with chilled 1X PBS followed by treatment with 10 μL of (50 μM) DAPI for 20 min. For properly fixing the cells, the media was again aspirated, and wells were washed multiple times with chilled 1X PBS. Afterwards 1 mL of PHEMS buffer containing 4% paraformaldehyde was added and incubation was done for 20 min. The cells were finally washed with cold PBS and mounted on the clean glass slide using mounting media. Imaging was carried out on Leica, SP-5 confocal laser scanning microscope at 20X magnification with water immersion.

A similar study was performed with cortical neurons and CATH.a cells in presence of cell trafficking inhibitors viz., chlorpromazine (CPZ, inhibitor for clathrin-mediated endocytosis), methyl β-cyclodextrin (MβCD, inhibitor for caveolae-mediated endocytosis), cytochalasin B (GLUT-inhibitor) and phloretin (GLUT inhibitor) 44, 45. For this, the overnight grown cells on the poly-L lysine coated cover glasses in 24 well plate were treated with CPZ (10 μg/mL, 30 µM), MβCD (2.5 mg/mL, ~2 mM), cytochalasin B (10 μg/mL, 20µM) and phloretin (300 μg/mL, ~1 mM) for 1 h at 37 °C in a CO_2_ incubator. The inhibitors were aspirated, and the cells were carefully washed with 1X PBS thrice and further incubated with *2DG-D-Cy5* for 4 h at 37 °C and then washed again with chilled 1X PBS. Further, the cells were fixed by 4% paraformaldehyde (equal volumes of 8% paraformaldehyde and 2X PHEMS buffer at room temperature for ~20 minutes. The fixative was aspirated, and the cells were washed multiple times with cold 1X PBS. The fixed cells were permeabilized by treating with 0.2% of triton-X-100 for ~10 min and washing with cold PBS again. For CATH.a neuronal culture, the staining was carried out with 10μL of (50 μM) DAPI for 20 min, followed by washing and staining with 20 μL of PhenoVue Fluor 568-Phalloidin (~0.4 nM) for 1 h, followed by washing with cold 1X PBS and imaging was carried out on Leica, SP-5 confocal microscope. In contrast to CATH.a neurons, for primary cortical neurons immunofluorescence staining procedure was carried out to specifically probe and differentiate the neurons from the glial cells present in the primary culture. For this, the triton-X-100 permeabilized cells were blocked with 5% bovine serum albumin (1X PBST) for 1 h at RT to limit the false positive staining. After blocking was done, the solution was aspirated, and cells were washed multiple times with 1X PBST. The blocked cells were incubated with primary anti-Nestin antibody (1:100) and kept at 4 °C overnight. After primary antibody treatment, the cells were thoroughly washed using PBST on a dancing shaker for 15 min (3 washes). For probing Nestin the cells were incubated with PhenoVue Fluor 488-Rat anti-mouse IgG1 secondary antibody for 2 h at 37 °C. Following this, the cells were again washed using 1X PBST thrice (3 X 5min) and stained with DAPI as mentioned above. The cover glass containing the stained cells were finally mounted on the cleaned glass slides using mounting medium and imaging was carried out on Leica, SP-5 microscope as mentioned above. All the measurements were done in replicates with proper controls.

### *Ex vivo* dendrimer quantification

The frozen organs (heart, lungs, liver, kidneys, spleen, and brain) were gradually thawed on ice, and weighed. The tissue samples were dissected to measure known amounts of tissues from each organ. The brain was dissected to separate the injured and non-injured regions. The tissue samples underwent homogenization with stainless steel beads in methanol at a 1 ml:100 mg tissue ratio using a tissue homogenizer. The homogenized samples were centrifuged at 4 °C and the clear supernatant was transferred to protein LoBind Eppendorf tubes and stored at -80 °C. For fluorescence quantification, the thawed supernatants were centrifuged again, fluorescence intensity was measured using Fluoromax spectrofluorophotometer. Fluorescence intensities for Cy5 (λex = 645 nm, λem = 662 nm) were determined and were adjusted for background fluorescence from control tissue. The fluorescence intensity values were converted to *2DG-D-Cy5* concentrations using calibration curves of *2DG-D-Cy5* at different slit widths. Serum, diluted 10-fold in Dulbecco's PBS, was also measured after filtration.

### Hemolysis/ hemocompatibility assay

With the rationale of using these molecules for *in vivo* studies, the hemolysis assay was carried out to see the effect of *2DG* and *2DG-D-Pio* on the rat red blood cells (RBCs) 46. In brief, ~5 mL of rat RBCs were diluted with 15 mL of 1X PBS (pH 7.4). Further, 250 μL of the RBC solution was added to 250 μL of *2DG* or *2DG-D-Pio* dendrimer at different concentrations (5, 2.5, 1.25, 0.63 and 0.31 mg/mL) in a micro centrifuge tube. All these samples were then incubated for almost 3 h at 37 °C in an incubator shaker at a rotation of ~80 rpm. Further, the resulting solution was carefully centrifuged at 5000 rpm for 10 min at room temperature and ~200 μL of supernatant from each of the dendrimer treated RBC samples was taken into a 96 well plate for evaluating the absorbance values recorded at 540 nm which corresponds to the hemoglobin release from the RBCs, using Thermo Scientific Multiskan SkyHigh Microplate reader. The RBCs that were treated with the 1X PBS (10 mM, pH 7.4) were kept as a negative control, whereas 1 % triton-x -100 treated RBCs were taken as positive control for hemolysis. From the recommendation by ASTM E2524-08 standard, any materials showing less than 5 % hemolysis can be considered as hemocompatible 46. The percentage hemolysis was calculated by following equation (2).



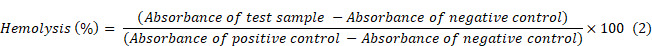



### *In vivo* studies experimental section

#### Biosafety studies

Animal studies: To investigate systemic toxicity, 3 week-old C57BL/6 mice (3 males and 3 females per group) were purchased from Jackson Laboratory. After 1 week of acclimation, mice were randomly separated into 3 groups: 1) Saline group: mice were i.p. injected with saline as blank control. 2) *2DG-D-Pio* group: mice were i.p. injected with *2DG-D-Pio* at the dose of 5 mg/kg *Pio* equivalent. 3) *2-DG-D* group: mice were i.p. injected with equivalent *2-DG-D* dendrimer solution as control dendrimer platform. All three groups were administered as a single intraperitoneal injection. Mice were weighed daily for 3 days after drug injection. On day 3, the liver and kidney were harvested for histological analysis. Blood was collected and then centrifugated at 3000 rpm for 15 min. The serum was collected for biochemical analysis following the kit instructions, including Alanine aminotransferase (ALT, BioAssay System, EALT-100) and Aspartate aminotransferase (AST, BioAssay System, EASTR-100), creatinine (CRE, BioAssay System, DICT-100), and urea nitrogen (BUN, BioAssay System, DIUR-100), following the assay instructions.

Histology Examination: Mouse liver and kidney tissues were fixed in 4% v/v phosphate-buffered formaldehyde, embedded in paraffin, sectioned, and stained with hematoxylin and eosin (H&E).

#### Efficacy studies

##### Animals

Male and female C57BL/6 mice (2-3 month of age; Jackson Laboratory, Bar Harbor, ME) were in-house bred. All of the pups were delivered naturally and remained with their mother after birth until weaning. All animals were housed under ambient conditions (20-22 °C, 40-60% relative humidity, and a 12-h light/dark cycle) with free access to food and water. Multiple precautions, including adequate habituation, gentle handling, minimization of procedure duration, and the use of humane endpoints according to “Recognition and Alleviation of Distress in Laboratory Animals” 47, were taken throughout the study to minimize pain and stress associated with experimentation. All experiments followed the Guide for the Care and Use of Laboratory Animals, eighth edition, published by the National Research Council (National Academies Press, 2011). Experimental procedures were approved by the Institutional Animal Care and Use Committee (IACUC) of the University of Michigan.

##### Impact acceleration model of TBI

On postnatal day 20-21 (P20-21), male (M) and female (F) animals (n = 73, 38M/35F) from the same litter were randomized into Sham (n = 17, 9M/8F) and TBI (n = 52, 29M/27F) groups using a random number generator. Randomization was stratified by sex. Anesthesia was induced with 4% isoflurane and tail and/or paw pinches were used to ensure the animal was fully sedated. The TBI animals underwent injury procedure as previously described 48, 49. In brief, anesthesia was induced with 4% isoflurane and tail and/or paw pinches were used to ensure the animal was fully sedated. After fully anesthetized, the animal was placed chest-down on a platform with a trapdoor that supported the body weight of a mouse (~7-10 g body weight) with little to no resistance or restraint upon impact. The animal's head was directly in the path of a falling weight. A weight (30 g) was held at 1.0 meter above the platform and secured by a pin. The lab personnel pulled the pin, allowing the weight to fall vertically through a guide tube to strike the animal on the head in the midline between bregma and lambda (at approximately bregma -2.5 mm). The animal rapidly underwent a 180° rotation, falling through the trapdoor and landing in a supine position on a cushion. The animal was removed immediately from the apparatus and placed in a clean warm cage. Sham animals were anesthetized with 4% isoflurane without TBI impact. All animals were closely monitored postoperatively with weight and health surveillance recordings, as per IACUC guidelines.

##### 2DG-D co-localization with neurons

Male and female TBI mice (n = 2 per sex) received intraperitoneal administration of fluorescent *2DG-D* (50 mg/kg, 100 μL) at 6-h post-injury, and were euthanized at 24-h post-injection. Brains were removed, postfixed in 10% formalin for 48 h, and then cryoprotected in 30% sucrose (in PBS). Coronal sections (20 µm, 1:6 series) were prepared on a cryostat (Leica Microsystems, IL, USA). Brain sections were incubated overnight at 4 °C with rabbit anti-NeuN (a neuronal marker; 1:250, Abcam, MA. U.S.A.) or rabbit anti-IBA1 (a microglial marker; 1:250, FUJIFILM Wako Chemicals U.S.A. Corporation, VA. U.S.A). Sections were subsequently washed and incubated with fluorescent secondary antibodies (1:250; Life Technologies, MA, U.S.A.) for 2 h at room temperature. The slides were dried, and cover-slipped with fluorescent mounting medium with DAPI (Sigma-Aldrich, MO, USA). Images were acquired using Nikon Eclipse TS2R fluorescent microscope (Nikon, NY, USA).

##### Bio distribution study of 2DG-D-Cy5

The mice from the same litter (n = 24, 12M/12F) were randomly divided into sham (n = 6, 3M/3F) and TBI (n = 18, 9M/9F) groups. The animals received intraperitoneal administration of *2DG-D-Cy5* (50 mg/kg, 100 μL) at 6-h post-injury. Mice in the TBI group were euthanized at 1, 3 and 24-h post-injection (n = 6, 3M/3F, per time point). Mice in the sham group were euthanized at 24-h (n = 6, 3M/3F) post-injection. Animals were transcardially perfused with PBS. The brain (injured regions and non-injured regions), heart, lungs, liver, spleen, kidneys, plasma, and urine were harvested.

##### *In vivo* pioglitazone/dendrimer-pioglitazone administration

Mice in the TBI group were randomized into TBI+saline (n = 11, 6M/5F), TBI+pioglitazone (TBI+*Pio*) (n = 11, 6M/5F), and TBI+Dendrimer- pioglitazone (TBI+*2DG-D-Pio*) (n = 12, 6M/6F) groups. Animals received intraperitoneal administration of free *Pio* (5 mg/kg, 100 μL), *2DG-D-Pio* (containing 5 mg/kg pioglitazone, 100 μL) or PBS (100 μL) at 6-h post-injury. The mice from the sham group (n = 11, 6M/5F) did not receive any intervention.

##### Body weight

Body weight was measured before injury (baseline) and at 1-day (d) post-treatment. The changes in the body weight were calculated as: (Body weight)_change_ = (body weight)_1d_ - (body weight)_baseline._

##### Behavioral tests

All of the behavioral testing was performed between 7AM to 6 PM. Mice were habituated in the test room for at least 30 min before the behavioral tests 48, 50. The lab personnel were blinded to experimental groups.

**Grip strength:** Muscular strength was evaluated with a grip strength test using a grip strength meter (BIOSEB, FL, USA) before injury and at 1-d post-treatment. In brief, the grip strength meter was positioned horizontally, and the animals were held by the tail and lowered towards the apparatus. The animals were allowed to grab the metal grid and were then pulled backwards in the horizontal plane. The force applied to the grid was recorded as the peak tension. Each animal underwent a grip strength test in three consecutive trials. The results were recorded and averaged for each animal. The change in the grip strength before and after injury was calculated as: (grip strength)_ change_ = (grip strength)_ 24h_ - (grip strength)_ baseline_

**Rotarod:** Sensorimotor coordination, endurance, and fatigue resistance was evaluated with a touchscreen five station accelerating Panlab RotaRod for mouse (BIOSEB, FL, USA) before injury and at 1-d post-treatment based on a published protocol^51^. Each animal was situated on a stationary rod for 10 s, and the rod was then set in motion with an accelerating speed of 3-30 rpm. Each animal underwent three consecutive trials (5 min each). The latency to the first fall in each trial was recorded and averaged for each animal. The change in the latency to the first fall before and after injury was calculated as: (Latency)_ change_ = (latency)_ 24h_ - (latency)_ baseline_

**Tail suspension test:** The tail suspension test was performed at 1-d post treatment as previously described to evaluate depression-like behaviors 51. In brief, mice were suspended by taping their tails (three quarters of the distance from the base of the tail) to a vertical bar on a tail suspension stand. The animal tail was aligned with the bottom of the bar. The animals' activities were monitored continuously for 6 min. The time spent immobile over the 6 min period were quantified and compared among groups.

**Light/dark box test:** The light/dark box was purchased from Stoelting Co. (Wood Dale, IL, USA), and the test was modified from published protocols to evaluate anxiety-like behaviors 52. The test was performed at 1-d post treatment. In brief, mice were placed in the middle of the brightly illuminated chamber and were allowed to move freely between the light and dark chambers for 10 min. Video recording was used to record animal behaviors. The time spent in the light chamber and the number of transitions between the light and the dark chambers was recorded and analyzed.

**Novel object recognition:** The novel object recognition test was modified from published protocols 50, 53 and performed at 1-d post treatment. In brief, the test was composed of two trials. The mice explored two identical objects for 5 min during the “training trial” and then were placed back in their cages. After an inter-trial break of 4-h, one of the previously exposed “old” objects was replaced with a new “novel” object, and the animals were allowed to explore these two objects for 5 min during the “probe trial”. The discrimination index for the probed trial was used to analyze the cognitive outcomes. Discrimination index=time spent exploring the novel object/(time spent exploring the old object + time spent exploring the novel object)×100%.

#### Isolation of primary neurons

Primary neuron isolation was modified from a published protocol 54, 55. In brief, brains were harvested and rinsed in HBSS solution on ice. Meninges were removed and the area of injury (approximately between bregma +2 mm and bregma -1 mm) and the area of non-injury (approximately between bregma -1 mm and bregma -3 mm) in the TBI mice, and the matching area in the sham mice were micro-dissected as previously described 48. Brain tissues were transferred to HABG solutions [60 mL HA, 1.2 mL B27, 0.176 mL Gln (0.5 mM final)], and minced (~0.5 mm) on ice. Brain tissues were incubated in HABG solution for 8 min at 30ºC in a Boekel shaking incubator with a shaking speed of 90 rpm (Cole-Parmer, Vernon Hills, IL, USA). Tissues were transferred to papain solutions [12 mg papain solids per 6 mL HA-Ca, 0.015 mL Gln (0.5 mM final)], and incubated for 30 min at 30ºC in a shaking incubator with a shaking speed of 90 rpm. Tissues were washed in HABG solution for 5 min at room temperature, triturated with sterile pipette for 45 s, and sit at room temperature for 1 min. The supernatants were collected, and the trituration was repeated two times. The supernatants were combined and centrifuged in OptiPrep™ Density Gradient Medium at 800 xg for 15 min at 22 ºC, and the fractions of neurons were collected as previously described 54. Cells were washed in HABG solutions and centrifuged at 200 xg for 2 min at 22 ºC. Supernatants were removed, and cells were washed in HBSS solution and centrifuged at 200 xg for 2 min at 22 ºC. Cell pellets were harvested for RNA isolation.

#### RNA isolation and quantitative real-time polymerase chain reaction (qPCR)

The mRNA expression of TNF-α (tumor necrosis factor alpha), IL-1β (interleukin-1 beta), IL-4, IL-6, IL-10, IL-13, TGF-β1 (transforming growth factor beta 1), iNOS (inducible nitric oxide synthase), NLRP3 (NLR Family Pyrin Domain Containing 3), and TLR4 (Toll-like receptor 4) were measured. The neurons isolated from the injured brain tissues (or the matching area of sham) were micro-dissected for RNA isolation as previously described 48. The total RNA was extracted using TRIZOL (Sigma-Aldrich, MO, USA), according to manufacturer's instructions. RNA samples were quantified using the Nanodrop ND-2000 Spectrophotometer (Thermo Fisher Scientific, MA, USA). Single-stranded complementary DNA (cDNA) was reverse transcribed from RNA using the High-Capacity cDNA Reverse Transcription Kit with RNase inhibitor (Thermo Fisher Scientific, MA, USA). qPCR was performed with iTaq(tm) Universal SYBR(R) Green Supermix (Bio-Rad, CA, USA) with CFX connect real-time PCR detection system (Bio-Rad, CA, USA). Amplification conditions included 30 sec at 95 °C, 40 cycles at 95 °C for 5 sec, and 60 °C for 30 sec. Primers were custom designed (**Table 1**) and ordered from Integrated DNA Technology (Coralville, IA, USA). The comparative threshold cycle (Ct) method was used to assess differential gene expressions. The sham group was the reference group, and glyceraldehyde 3-phosphate dehydrogenase (*Gapdh*) was the housekeeping gene. Gene expression levels for each sample were normalized to the expression level of *Gapdh* within a given sample (ΔCt); the differences between sham and TBI groups were used to determine the ΔΔCt. The 2-ΔΔCt gave the relative fold changes in gene expression.

#### Statistical analysis

Data were analyzed using GraphPad Prism 6 (Version 6.04; CA, USA). All data were presented as mean ± SEM. D'agostino and Pearson omnibus normality test was used for normality measurement. Two-way ANOVA and Bonferroni post hoc tests were used for multiple group comparisons. Statistical significance was set at p < 0.05 for all analyses.

## Results and Discussion

We rationally developed the *2DG-D* guided by the following principles. (1) We utilized biocompatible building blocks and Generally Recognized As Safe (GRAS) reagents, with approximately 17% 2DG, 57% polyethylene glycol (PEG), 13% triazole, 9% core, and 4% gallic acid composition. (2) We incorporated *PEG* and *2DG*-based building blocks, which are inexpensive and non-toxic. (3) We employed an efficient convergent synthetic approach to reduce synthetic complexity. (4) We created the *2DG-D* with neutral surface charge and small size for enabling extravasation through impaired blood vessels and easy movement in the brain tissue parenchyma 56. The *2DG-D* is a generation-3 mixed-layer glycodendrimer synthesized in an expedited manner using a combination of hypermonomer strategy, convergent synthesis, and highly efficient and orthogonal copper (I) catalyzed click chemistry (CuAAC) approach. The innermost layer, the core, comprises alkyne-terminating generation-1 PAMAM dendrimer, followed by a second layer composed of gallic acid building blocks, and the outermost layer consists of *2-DG*. In contrast to the conventional method of dendrimer synthesis, where identical building blocks are employed sequentially throughout the generations, our approach involves utilizing a diverse combination of building blocks. This synthetic method offers us the flexibility to efficiently create dendrimers while allowing us to achieve entirely distinct structures with ease simply by altering the arrangement of building blocks within a given generation. The *2DG-D* is highly soluble in water and there are 72 hydroxyl groups on the surface which can be easily manipulated to attach bioactive molecules of interest.

### Synthesis and characterization of 2DG-D

**Figure 1:**
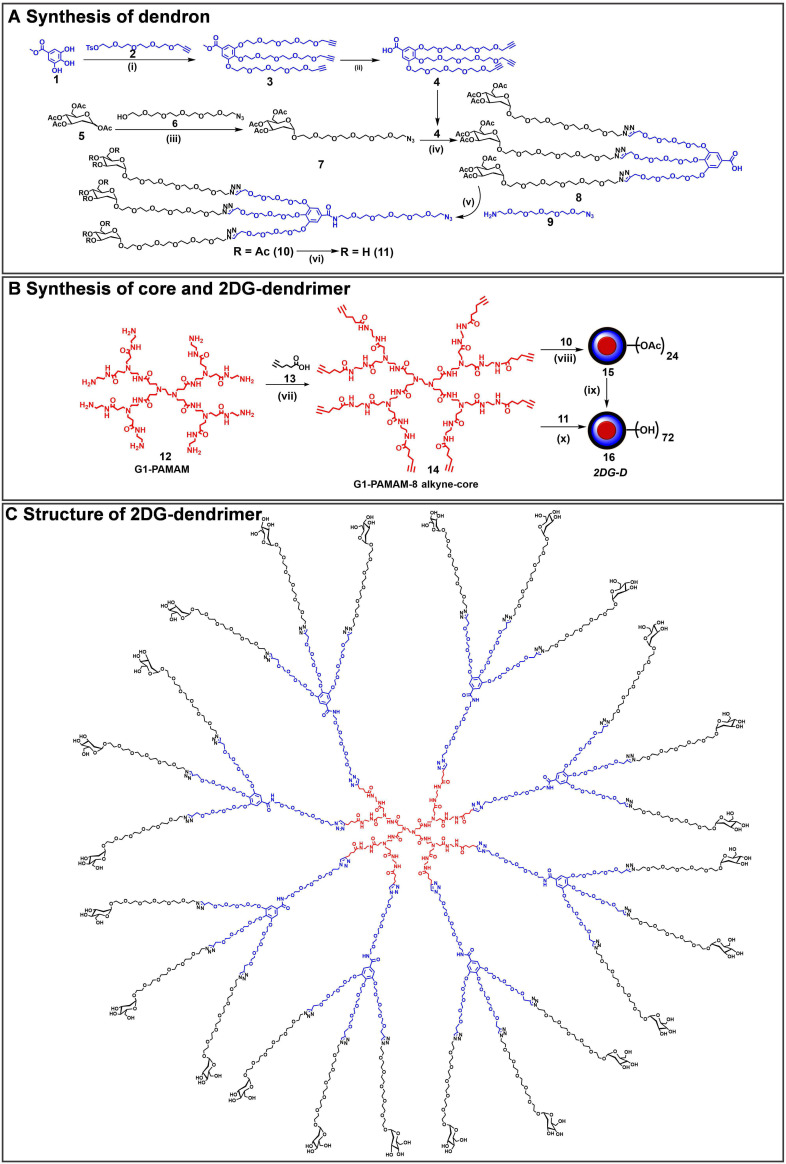
**Synthetic route to 2DG-dendrimer (*2DG-D*). A)** Synthetic route to a clickable dendron containing three 2DG molecules; **B)** Synthesis of *2DG-D* from 8-arm alkyne core; **C)** structure of *2DG-D* depicting 24 -2DG molecules at the surface. *Reagents and conditions:* (i) K_2_CO_3_, DMF, 16 h, 50 °C, MW, 60%; (ii) LiOH.H_2_O, THF:H_2_O, RT, 48 h, 95%; (iii) BF_3_.OEt, Dry DCM, 24 h, RT, 50%; (iv) CuSO_4_.H_2_O, Sod. Ascorbate, DMF, 40^o^C, MW, 10 h, 90%; (v) EDC.HCl, HOBt, DCM, RT, 1.5 h, 90%; (vi) NaOMe, MeOH, RT, 24 h, 89% (vii) EDC.HCl, DMAP, DMF, 16 h, RT, 84%; (viii) CuSO_4_.5H_2_O, Sodium ascorbate, 40 °C, MW, 10 h, 90%; (ix) NaOMe, MeOH, RT, 24 h, 90%; (x) CuSO_4_.5H_2_O, Sodium ascorbate, 80 °C, MW, 15 h, 95%.

**Figure 2:**
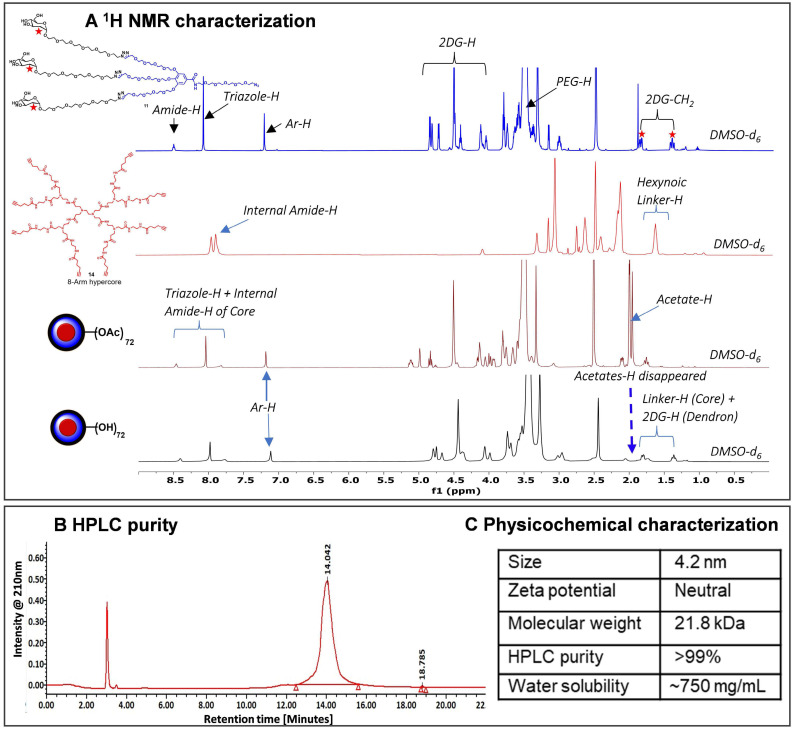
**Characterization of 2DG-dendrimer. A)** ^1^H NMR spectra of intermediate dendron, hyper-core, protected and deprotected *2DG-D* representing the appearance and disappearance of characteristic protons; **B)** HPLC chromatogram of *2DG-D* showing >99% purity; **C)** Table representing the physicochemical properties of *2DG-D*.

The *2DG-D* synthesis was initiated with the preparation of a clickable G2 dendron (**Figure 1A**). Methyl gallate (**1**) was alkylated at three hydroxyls with propargyltetraethyleneglycol-4-methylbenzenesulfonate (**2**) to afford tri-alkynated compound (**3**). The ^1^H NMR analysis clearly showed the characteristic alkyne signals (3H) at δ 2.43 ppm (**Figure S1**). This was followed by hydrolysis with lithium hydroxide to produce compound (**4**) in 80% overall yield. On the other hand, peracetylated 2-deoxy-D-glucose (**5**) was modified with a short PEG linker with azide focal point. This involved the glycosylation of 2DG with azido-HEG-alcohol (**6**) in presence of Lewis acid BF_3_.OEt_3_, resulting in compound (**7**) as α-anomer (α_D_^20^= 53.8° (c= 1, CHCl_3_). The resultant compound (**7**) was then clicked to the three acetylene branches of gallic acid monomer (**4**) *via* CuAAC reaction under microwave irradiations to yield compound (**8**). The disappearance of acetylene protons and the appearance of triazole (3H) protons at δ 7.69-7.75 ppm confirmed the completion of click reaction (**Figure S10**). Furthermore, the proton NMR spectrum also revealed the presence of acetate protons (27H) linked to three 2DG sugars, exhibiting chemical shifts within the range of δ 1.96-2.03 ppm. The carboxylic acid group on compound (**8**) was then reacted with azido-PEG-5-amine (**9**) using EDC-HOBt promoted amide coupling to afford acetyl-protected dendron (**10**). Appearance of characteristic amide proton (N*H*) at δ 8.46 ppm as well as additional PEG protons in the NMR confirmed the structure of compound **10** (**Figure S13**). Subsequently, de-*O*-acetylation of protected dendron (**10**) under Zemplén transesterification conditions (NaOMe, MeOH) provided the final deprotected 2DG azide dendron (**11**) in quantitative yield **(Figure 1A)**. The ^1^H NMR clearly showed the disappearance of acetate protons (**Figures 2A** and** S15**).

Next, we synthesized the core based on PAMAM generation-1 amine dendrimer (**12**) which carried 8 amine functional groups on the periphery. It was reacted with the 5-hexynoic acid (**13**) in the presence of amide coupling reagents to produce 8-armed acetylene functionalized core (**14**) in 80% yield (**Figure 1B**). The complete per-NH_2_-acetylene insertion at all eight terminals was confirmed by ^1^H NMR analysis, which showed the characteristic methylene (-CH_2_) protons (16*H*) of attached hexynoic acid linker at δ 1.62-1.69 ppm and additional new amide protons (8-N*H*-) along with internal amides (12-N*H*-) of G1 PAMAM dendrimer at 7.61-8.21 ppm (**Figures 2A and S18**).

Finally, the synthesis of the *2DG-D* was carried out through two separate synthetic methods, one involving protected 2DG-G2 azide dendron (**10**) and the other using unprotected 2DG-G2 azide dendron (**11**) as depicted in **Figure 1B**. To construct the dendrimer *via* the protected route, the CuAAC click reaction was performed between the octa-alkyne G1 PAMAM dendrimer core (**14**) and the peracetylated 2DG-azide (**10**) using classical click reagents, a catalytic amount of copper sulfate pentahydrate (5 mol% per alkyne), and sodium ascorbate (10 mol% per alkyne). The rection was carried out under microwave (MW) irradiation at 40^o^C for 10 h to achieve 2DG-OAc dendrimer **15** (**Figure 1B**) in excellent yield. The appearance of new triazole (8H) protons at δ7.7 ppm confirmed the completion of click. In addition, the number of acetate protons (216H) at δ 1.93-2.01 ppm corresponds to the attachment of 24 -2DG units further confirming the desired structure (**Figures 2A** and **S20**). The deprotection of acetate groups was achieved *via* Zemplén transesterification (NaOMe in methanol) to yield the final dendrimer *2DG-D* (**16**) with 72-OH in 95% yield. The disappearance of acetate peaks in the proton NMR confirmed the formation of the deprotected product (**Figures 2A, S22**). We used protected route to confirm the successful participation of all acetylene branches in the click reaction through the presence of acetate protons in the NMR which made characterization easier and allowed us to observe any defects or missing arms in the final dendrimer. Within the ^1^H NMR spectra, the acetyl protons associated with eight dendrons of 2DG were evident as separate, non-overlapping peaks, distinct from the other protons present in the macromolecule. These characteristic peaks offered a precise way to confirm the successful completion of the reaction. The same deprotected dendrimer **16** was synthesized by the reaction of PAMAM core (**14**) with unprotected 2DG-azide dendron (**11**) *via* click reaction at 80^o^C under MW irradiation in a 95% yield. The *2DG-D* was purified through tangential flow filtration (TFF) utilizing a 3 kDa cassette. As per the HPLC data, the final dendrimer displayed a purity level of >99% (**Figures 2B** and** S24**).

The complete structure of *2DG-D* is shown in **Figure 1C**. The physicochemical properties of *2DG-D* are presented in **Figure 2C**. The *2DG-D* exhibits high water solubility (750mg/mL) which limits the need of excipients for systemic formulations. The size of *2DG-D* is 4.2 nm which allows the clearance from off-target organs through renal filtration (**Figure S26**). The *2DG-D* exhibits neutral zeta potential which is important for the movement of dendrimer within the brain tissue parenchyma (**Figure S27**) 56. Both the protected and unprotected synthetic approaches resulted in the production of *2DG-D* with excellent yields, high purity, and consistent reproducibility. All intermediates and the final dendrimer were characterized *via* NMR and mass spectroscopy **(Figures S1-S25)**. The synthetic approach employed here doesn't necessitate the use of excessive chemicals or reagents as is often required in traditional dendrimer synthesis. Commercially available and most widely used PAMAM dendrimers are typically manufactured using large amounts of reactants, which still frequently results in structural defects at higher generations due to sluggishness of the chemical reactions at multiple ends 57. On the contrary, this entire synthetic process presented here is green, reproducible, and cost-effective, making it well-suited for commercial applications.

### Reproducibility of 2DG-D synthesis

**Figure 3:**
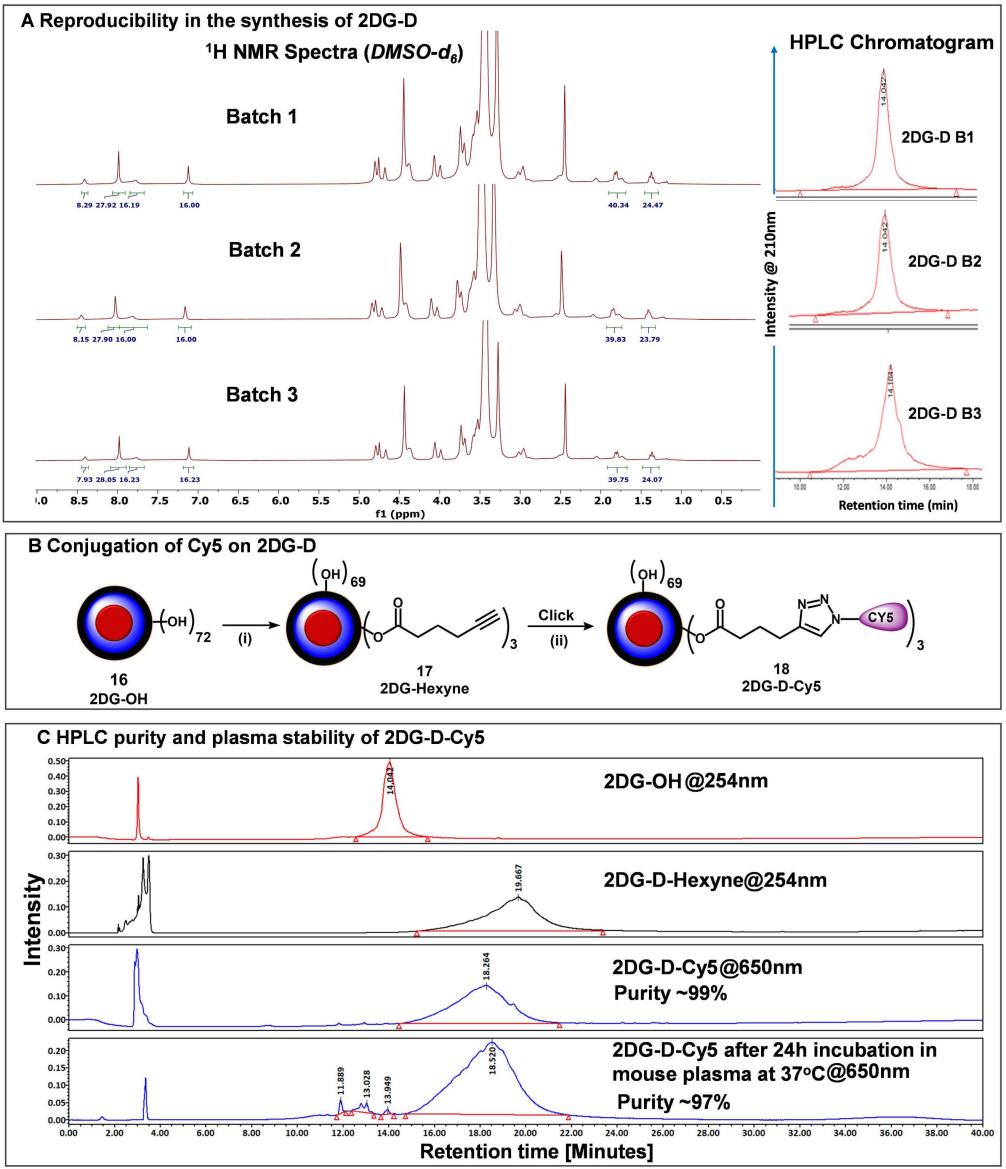
**Reproducible synthesis of *2DG-D* and Cy5 conjugation. A)** The figure shows the reproducibility in the synthesis of multiple 5 g-scale batches of *2DG-D via* ^1^H NMR and HPLC. The ^1^H NMR spectra of 3 batches (5 g scale) show characteristic protons from *2DG-D*. The HPLC chromatographs show reproducibility in the purity of these batches with all these batches showing >99% purity. **B)** conjugation of near-infrared dye Cy5 on *2DG-D* for imaging and biodistribution studies. *Reagents and conditions:* (i) 5-hexynoic acid, EDC.HCl, DMAP, DMF, 16 h, RT, 84%; (ii) Cy5-azide CuSO_4_.5H_2_O, sodium ascorbate, 40 °C, MW, 10 h, 87%.**C)** HPLC chromatogram showing a shift in retention time during *2DG-D-Cy5* synthesis. The *2DG-D-Cy5* is stable when incubated in plasma at physiological temperature for 24 h.

The major hurdle in the clinical translation of nanoparticle-based therapeutics is the lack of reproducibility and scalability of their synthesis process. To address these issues, we synthesized *2DG-D* using a simple, convenient, and expedited synthetic strategy that allowed the precise characterization of intermediates at each step as demonstrated above. To further validate the reproducibility in the synthesis of *2DG-D*, we constructed several 5 g-scale batches of *2DG-D* and compared their ^1^H NMRs and purity by HPLC. ^1^H NMR spectra of three different batches (**Figure 3A, left**) clearly depicted the presence of 24 -2DG molecules on the surface, showing consistency in the synthesis of *2DG-D*. The HPLC chromatogram of these three batches of *2DG-D* showed corresponding peaks at same retention time (14 minutes), confirming the consistency in the purity of these batches (**Figure 3A, right**).

### Fluorescently labeling of 2DG-D

To study the *in vivo* brain cell uptake and organ biodistribution of *2DG-D via* confocal microscopy and fluorescence spectroscopy, we introduced a near-infrared dye cyanine 5 (Cy5) at the surface of *2DG-D*. We modified ~3 hydroxyl groups on the surface of *2DG-D* by reacting with 5-hexynoic acid in the presence of EDC-HOBt to afford compound (**17**) as illustrated in **Figure 3B**. The ^1^H NMR showed the presence of the additional methylene protons corresponding to hexynoic linker in the aliphatic region of dendrimer (**Figure S28**). The HPLC chromatogram showed a shift in retention time from 14 minutes for *2DG-D* to 19.6 minutes for *DG-D-Hexyne* (**17**). The *2DG-D-Hexyne* with acetylene groups on the surface was reacted with azide-terminating Cy5 using CuAAC reaction to obtain fluorescently labeled *2DG-D-Cy5* (**18**). The success of Cy5 attachment was confirmed by the presence of Cy5 protons in the ^1^H NMR spectrum (**Figure 3C**). The number of attached Cy5 molecules on the dendrimer surface was calculated using the proton integration method, suggesting the attachment of ~3 molecules of the Cy5 (**Figure S30**). The purity of *2DG-D-Cy5* was >98% by HPLC, and the HPLC chromatogram showed a clear shift in the retention time from 19.6 to 18.2 min upon conjugation of Cy5 (**Figure 3C**). The *2DG-D* was designed to target and deliver drugs intracellularly to neurons.

However, the dendrimer platform itself was designed to be non-biodegradable using non-cleavable linkages in the backbone. The fluorescent *2DG-D* dendrimers were stable at physiological conditions when incubated in plasma at 37 °C for 24 h (**Figure 3C**) making them suitable for *in vivo* uptake and biodistribution studies.

### Mechanism of 2DG-D uptake by the neuronal cells

**Figure 4:**
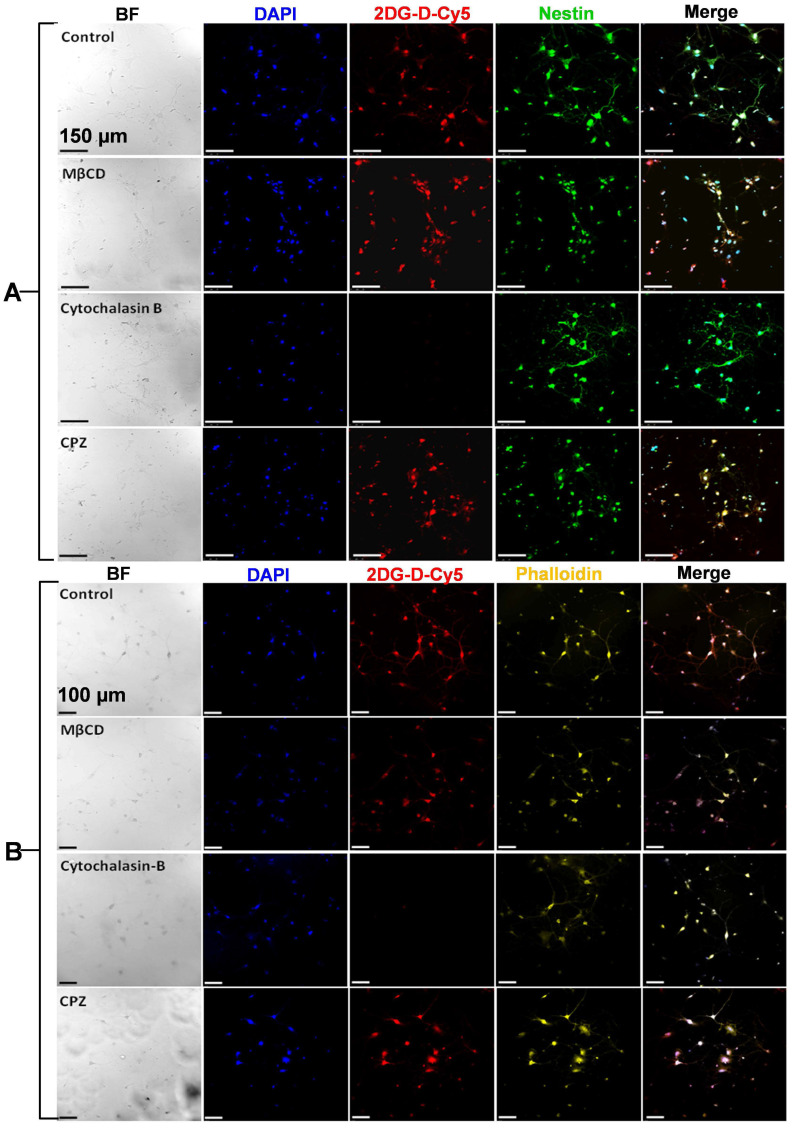
Confocal laser scanning micrographs showing uptake of the *2DG-D-Cy5* dendrimer by **A)** cortical nuerons and **B)** CATH.a neurons, dipicted by the red fluorescence of Cy5 inside the cells. The cells stained with blue, green and yellow, represent nucleus, nestin protein and actin microflament proteins respectively. The scale bar shown in the figure is 150 µm (**A**) and 100 µm (**B**). The images are representative of three independent experiments.

Before initiating *in vitro* uptake studies, we first evaluated the toxicity of the *2DG-D* in cortical neurons, CATH.a neuronal cells, and RAW Blue macrophages (**Figure S42**). The cells were cultured with a gradient of *2DG-D* concentrations up to 1000 μg/mL. The *2DG-D* dendrimer was found to be nontoxic to neurons and macrophages as determined by Two-way ANOVA (Turkey's multiple comparisons test), with no significant effect of treatments at all the concentrations, showing no signs of toxicity (**Figure S42**). Following this, we evaluated the dose-dependent uptake of *2DG-D-Cy5* by primary cortical neurons (**Figure S43**). The microscopy based qualitative uptake results suggested that there was a concentration dependent uptake of the *2DG-D-Cy5* by the cortical neurons, when increased from ~3.13 µg/mL to 100 µg/mL, however, the uptake tended to be saturated when the dendrimer concentration reached above 100 μg/mL. Hence, 100 μg/mL *2DG-D-Cy5* was considered optimal concentration and chosen for the further inhibitor-based uptake studies. GLUT3 is the key transporter protein that is involved in the neuronal uptake of glucose 58. To decipher the actual mechanism for the dendrimer uptake by both primary and secondary neurons in the present study, intracellular trafficking inhibitor-based study using GLUT and other inhibitors was carried out (**Figures 4A and 4B**). From the results, it was observed that, in both cortical and CATH.a neurons, the MβCD treated cells did not show any qualitative reduction in the uptake of *2DG-D-Cy5* (red signal), which was comparable to the control cells (no inhibitor) even after the inhibition of caveolae dependent endocytic pathway. In contrast to this, when cytochalasin B was used as an inhibitor for GLUT transporters, there was negligible *2DG-D-Cy5* signal in both primary and secondary neuronal cells, depicting GLUT receptors as the possible internalization pathway. Further, treatment with CPZ, a clathrin endocytosis inhibitor had no significant impact on the uptake, which was quite comparable to control. The results obtained from cytochalasin B treatment were further confirmed with phloretin, a broad-spectrum GLUT inhibitor (**Figures S44a and S44b**). Similar to the results obtained with cytochalasin B, phloretin treated cells showed negligible *2DG-D-Cy5* internalization in both the neuronal cell types, which further confirmed that GLUT receptors were possibly the key players in the neuronal uptake of 2DG-D. The quantitative results obtained from the ImageJ analysis of the confocal micrographs revealed that in cortical neurons, the normalized MFI of control (~1), was almost similar to MβCD (~1) and CPZ (~1.03), however in comparison to this, the normalized MFI for cytochalasin B and phloretin were ~0.002 and ~0.006, which suggested that for all the non-inhibited samples, the MFI was close to 1 and for the inhibited ones, it was close to zero (**Figure S44c**). Similar results were observed in the case of CATH.a neurons, with normalized MFI values viz., ~1 (control), ~0.89 (MβCD), ~0.93 (CPZ), ~0.046 (Cytochalasin B), and ~0.0001 for Phloretin (**Figure S44d**). Hence from the quantitative results, it was clear that both primary and secondary neurons showed negligible uptake when incubated with GLUT inhibitors.

### Qualitative and quantitative brain and organ uptake of systemically administered fluorescently 2DG-D in a pediatric mouse model of TBI

**Figure 5:**
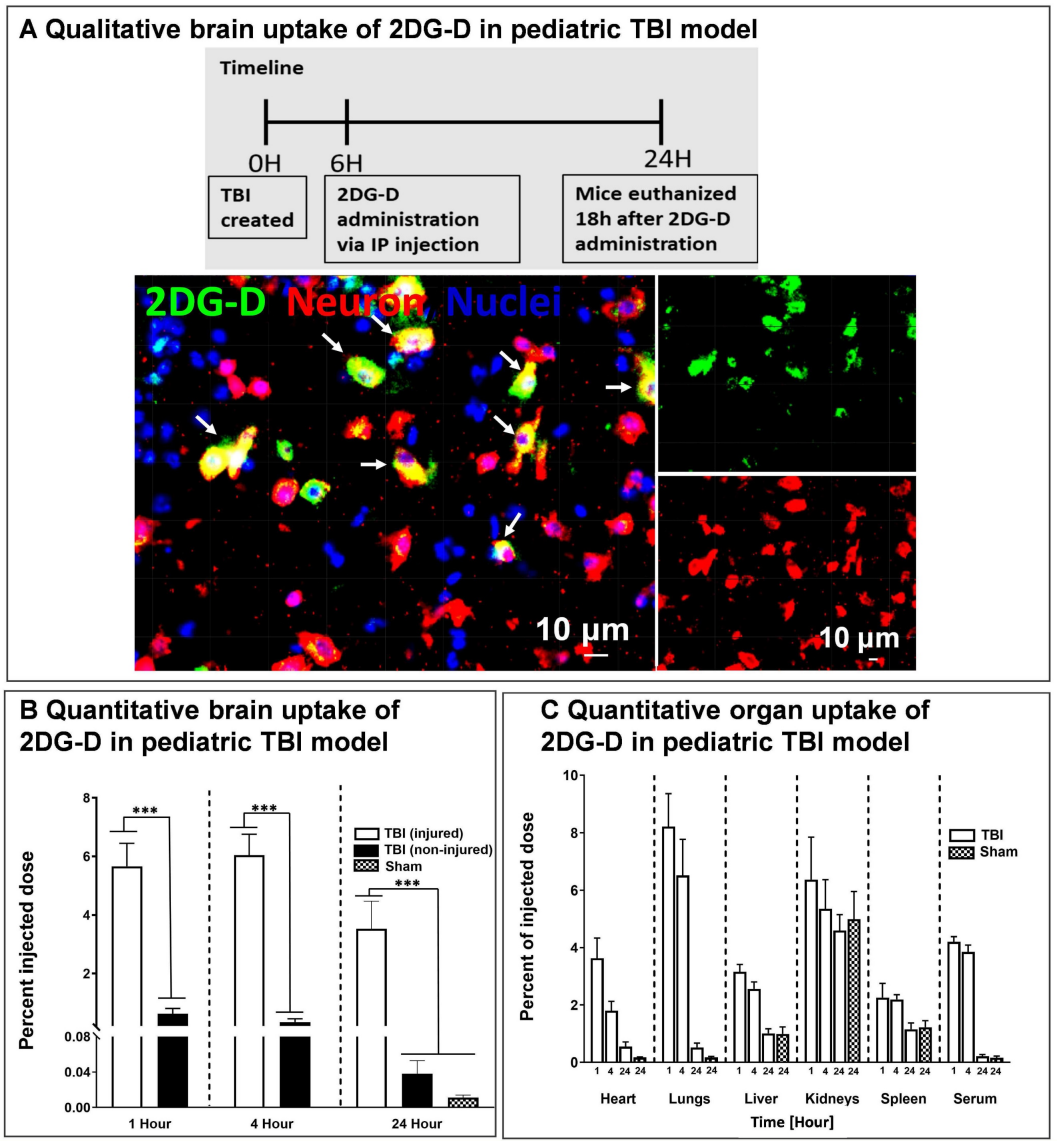
** Brain and organ biodistribution of *2DG-D* in a pediatric mouse model of TBI. A)** *In vivo* cellular localization of fluorescently labeled *2DG-D*. Male and female TBI mice (n = 2 per sex) received intraperitoneal administration of *2DG-D* (50 mg/kg, 100 μL) at 6 h post-injury, and euthanized at 24 h post-injection. Brain slices containing *2DG-D* (green) were co-stained with NeuN (neuronal marker, red) and DAPI (blue). The figure represents the co-localization of *2DG-D* and neurons at the site of injury. Scale bars: 10 µm. **B)** Quantitative biodistribution of *2DG-D-Cy5* in TBI animals in injured and non-injured regions at different time points (1, 4, and 24 h; n = 6-7) as compared to age-matched sham controls (n = 6). A significant increase in the dendrimer uptake was detected in the injured brain of TBI animals as compared to non-injured brain of TBI animals and to the brains of sham animals (****P < 0.0001; (***P < 0.001; (**P < 0.01). **(C)** Quantitative biodistribution of *2DG-D-Cy5* in the major organs and serum of TBI animals at different time points (1, 4, and 24 h; n = 6-7). The data was obtained through fluorescence spectroscopy of homogenized tissue extracts containing *2DG-D-Cy5* and reported as a percentage of the injected dose in total organ (or total serum volume).

We further investigated the *in vivo* brain and organ uptake and biodistribution of fluorescently labeled *2DG-D* in a pediatric mouse model of TBI. This impact acceleration TBI model replicates the pathophysiology that is commonly observed in humans caused by falls and reliably induces diffuse axonal injury in the absence of skull fractures and parenchymal focal lesions 48, 59, 60. To evaluate the cellular co-localization of dendrimer across the BBB at the site of injury in the brain, fluorescently labeled *2DG-D* conjugates were administered at 6-h post-injury and animals were euthanized at 24-h post-injection. The 6-h time point was chosen to mimic clinical situations. We found that *2DG-D* was co-localized with neurons in the injured brain region (**Figure 5A**). This indicates that the novel *2DG-D* can cross the BBB and achieve cell-specific localization in the brain of TBI mice. We did not observe such uptake in the healthy regions of the brain of TBI animals. Since apoptotic cell death of neurons is the major hallmark of TBI, targeting these cells and rescuing them at the site of brain injury can lead to potential neurotherapeutics.

We further investigated the quantitative distribution of *2DG-D-Cy5* in the brains and organs of TBI animals at three distinct time intervals (1, 4, and 24 h; n = 6-7) and compared it to age-matched sham animals (n = 6). We divided the brain of the TBI animals into injured and non-injured regions to quantify the region-specific uptake of *2DG-D-Cy5*. We perfused the animals with phosphate-buffered saline (PBS) to mitigate interference from blood and dendrimer lodged in blood vessels. Upon one way-ANOVA analysis, there was significant difference in the *2DG-D-Cy5* uptake (F=23.18, p < 0.0001). Specifically, 1) *2DG-D-Cy5* uptake was significantly higher in the injured brain regions in the TBI animals at 1-, 4- and 24-h post-injection, compared with the shams at 24-h post-*2DG-D-Cy5* administration (p < 0.05), which indicates higher uptake of *2DG-D-Cy5* in injured animals, compared to healthy animals. 2) *2DG-D-Cy5* uptake significantly increased in the injured brain regions, compared with the non-injured brain regions of the TBI animals at 1-, 4-, and 24-h post-*2DG-D-Cy5* administration (p < 0.0001), which indicates targeted delivery of the *2DG-D-Cy5* to the injured brain region, not the non-injured brain regions. 3) There was no significant difference in the *2DG-D-Cy5* uptake at the injured brain regions at 1-, 4-, and 24-h post-*2DG-D-Cy5* administration (p>0.05), which indicates the long-lasting sustained retention of the *2DG-D-Cy5* at the injured brain regions in the TBI animals. 4) There was no significant difference in the *2DG-D-Cy5* uptake between the sham animals and the non-injured brain regions of the TBI animals at 1-, 4-, and 24-h post-*2DG-D-Cy5* administration (p>0.05). This implies that *2DG-D-Cy5* is not taken-up and/or retained by non-injured or healthy brain tissues even shortly after injection, indicating fast clearance, leading to less unwanted side effects (**Figure 5B**). This specific uptake is significant while delivering therapies to the affected areas in the brain without affecting the healthy areas.

Next, we assessed the quantitative biodistribution of *2DG-D* in other organs. Because the off-target accumulation of nanoparticles is a major concern in their clinical translation, we designed the *2DG-D* with a size in the range of renal filtration to avoid any unwanted accumulation in any major organs. The biodistribution of *2DG-D* was assessed in all major organs (heart, lungs, liver, kidneys, and spleen) and serum. Upon one way-ANOVA analysis, there was no significant difference in *2DG-D-Cy5* uptake in the kidneys between the TBI and sham animals at 1, 4 and 24-h post-injection (F=0.5588, p = 0.6408). The presence of dendrimer in kidneys was due to the renal clearance mechanism. There was significant difference in the *2DG-D-Cy5* uptake in heart (F=16.41, p < 0.0001), lungs (F=25.19, p < 0.0001), liver (F=23.87, p < 0.0001), spleen (F=6.192, p = 0.0035), and serum (F=225.4, p < 0.0001). Specifically, *2DG-D-Cy5* level in the heart, lungs, liver, spleen, and serum of the TBI animals decreased at 24 h. The clearance of dendrimer from sham animals exhibited a similar trend at 24 h, and there was no significant difference in the *2DG-D-Cy5* level at 24 h post-injection between the sham and TBI animals. Less than 0.2% dendrimer was detected in the serum 24 h after injection for both TBI and Sham animals. The results indicate that there was a temporary accumulation of dendrimer in these organs, but it did not retain and cleared by 24 h time point. The clearance *via* kidneys has been extensively validated for similarly sized clinical stage PAMAM-OH (4 nm, neutral) dendrimers, and other similarly sized hydroxyl dendrimers 4, 27, 61. The precise brain uptake and retention coupled with the rapid clearance from off-target organs makes *2DG-D* a unique and promising nanoplatform for developing neuron-targeted therapies for CNS disorders.

### Synthesis and characterization of dendrimer-pioglitazone (2DG-D-Pio) conjugates

While originally approved for the treatment of non-insulin-dependent diabetes mellitus, thiazolidinediones or glitazones have shown promising neuroprotective effects in different CNS injury models 34-37, 62-64. *Pio* is a selective agonist of the PPARγ that has shown beneficial effects in the treatment of neuronal injury and inflammation following brain injury 34-38. However, to achieve these effects, high drug levels at the target site are essential. *Pio* exhibits very low water solubility (0.00442 mg/mL) 39, and low brain penetration 65, 66. To achieve minimum therapeutic level concentration an increased dose is required which may lead to hypoglycemia and other severe side effects like bone loss, edema, blood cell loss, and hepatotoxicity 40, 41. *2DG-D* mediated targeted intracellular delivery of *Pio* can 1) decrease the dose and systemic side effects, 2) enhance the efficacy to attenuate the ongoing neuronal injury and inflammation, and 3) improve the aqueous solubility by several folds.

**Figure 6:**
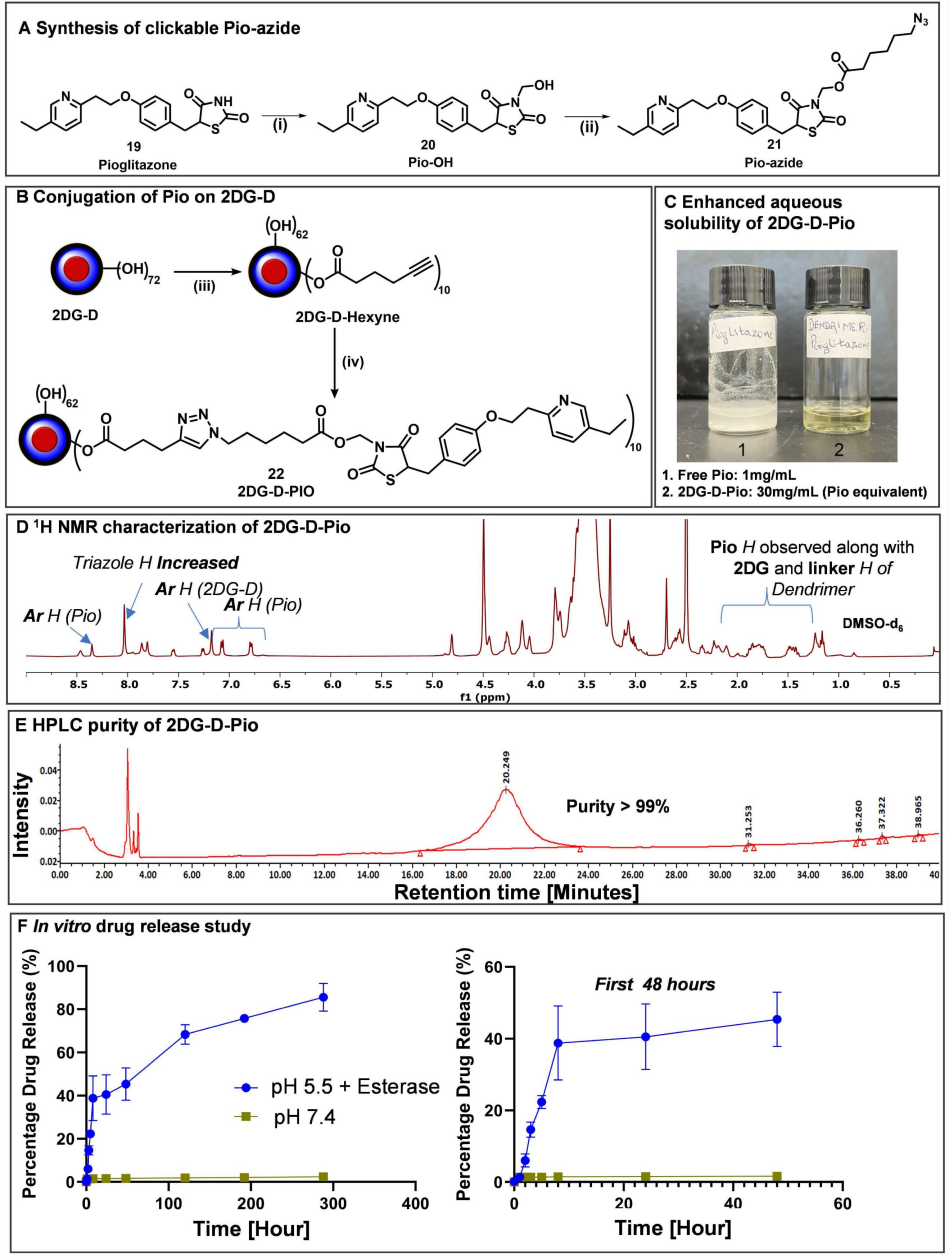
** Synthesis and characterization of 2DG-D-pioglitazone (*2DG-D-Pio*) conjugate. A)** Attachment of clickable linker on *Pio*; **B)** Conjugation of *Pio*-azide on *2DG-D* to synthesize *2DG-D-Pio* conjugate; *Reagents and conditions:* (i) Formaldehyde, Et_3_N, Anhy. DMF, 15 h, RT; 90 %; (ii) 6-azido-hexanoic acid, EDC.HCl, DMAP, Anhy. DMF, RT, 24 h, 70 %; (iii) 5-hexynoic acid, EDC.HCl, DMAP, DMF, 16 h, RT, 95%; (iv) CuSO_4_.5H_2_O, Sodium ascorbate, 40 °C, MW, 10 h, 95%. **C)** *2DG-D-Pio* demonstrates several folds higher water solubility than free *Pio*; **D)** ^1^H NMR spectrum of *2DG-D-Pio* depicting characteristic drug and dendrimer protons; **E)** *2DG-D-Pio* demonstrates >99 purity *via* HPLC; **F)** *2DG-D-Pio* shows sustained drug release profile at intracellular conditions.

To obtain the *2DG-D-Pio* conjugate, *Pio* containing a clickable linker (azide) was first synthesized. *Pio* (**19**) was treated with formaldehyde to produce *Pio*-OH (**20**), which was subsequently reacted with azido-hexynoic acid in the presence of EDC-DMAP to yield *Pio*-azide (**21**) as depicted in** Figure 6A**. On the other hand, *2DG-D* was modified to bring ~10 acetylene arms, which were reacted with *pio*-azide (**21**) using CuAAC click reaction to obtain *2DG-D-Pio* dendrimer conjugate **22** (**Figure 6B**). After dialysis purification and freeze-drying, the *2DG-D-Pio* was obtained in 95% yield. ^1^H NMR confirmed the attachment of 10 drug molecules per dendrimer corresponding to ~12 weight percent drug loading (**Figure 6D** and **S34**). The characterization of all the intermediates and final conjugates was carried out by NMR and mass spectroscopy (**Figures S1-S38**). The purity of *2DG-D-Pio* was ~99% by HPLC (**Figure 6E** and** S38**). The *2DG-D-Pio* **e**xhibited remarkable solubility in water exceeding 30 mg/mL (**Figure 6C**). This represents a substantial improvement in the solubility of *Pio* when conjugated to 2DG-D while the free drug exhibits negligible water solubility (0.00442 mg/mL) 39. The dendrimer conjugation remarkably improved the *Pio* water solubility by ~6000 folds.

We further evaluated the shelf stability of *2DG-D-Pio* formulation in PBS. The stability was assessed both at room temperature (RT) and 4 °C, over a period of 28 days. Remarkably, even after 28 days of storage, both at 4 °C and RT, the *2DG-D-Pio* formulations retained their stability, with purities of 99.34% and 98.24%, respectively as determined by HPLC (**Figures S39 and S40**). Importantly, there were no shifts in retention time observed during the entire 28-day period with no detectable release of *Pio* from the formulation under these storage conditions suggesting the stability of the conjugate.

Next, we conducted the *in vitro* drug release study from the conjugate, both in conditions mimicking the extracellular environment (physiological pH, PBS buffer at pH 7.4) and intracellular conditions under the influence of an enzyme (carboxyl-esterase, pH 5.5) (**Figure 6F**). In PBS buffer at pH 7.4, we detected negligible release of the drug, indicating the plasma stability of the *2DG-D-Pio* conjugate. To mimic the *in vivo* conditions, we further evaluated the stability of *2DG-D-Pio* in serum supported PBS. The *2DG-D-Pio* did not show any signs of degradation or *Pio* release over a period of 7 days as demonstrated by the HPLC chromatogram at various time points (**Figure S41**). This is important to maintain the target-specific intracellular delivery of the drug. At intracellular conditions, the dendrimer-drug conjugate exhibited a gradual and sustained release of the free drug. Over a 48 h period, less than 50% of the drug was released, and this release continued, reaching approximately 80% drug release over a 12-day period (**Figure 6F**). These release and stability studies provide valuable insights into drug release kinetics, which are relevant for designing treatment regimens and selecting appropriate time points for the *in vivo* studies.

### Preliminary biosafety studies

**Figure 7:**
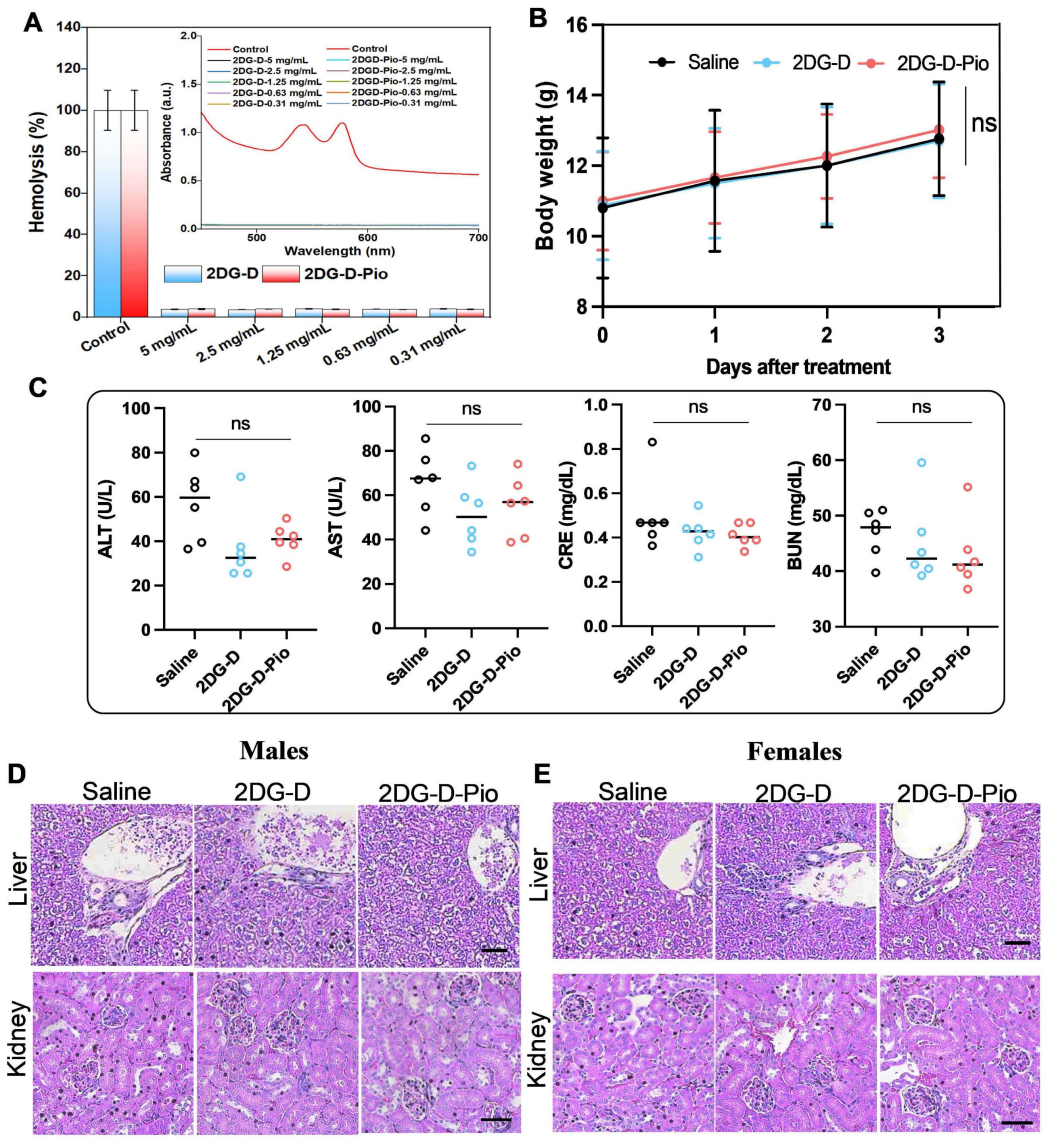
** Preliminary biosafety studies. A)** Hemocompatibility analysis of *2DG-D* and *2DG-D-Pio* dendrimer, histogram showing percentage hemolysis of *2DG-D* and *2DG-D-Pio* treated RBCs at different concentrations, the inset to (**A**) is spectrophotometric studies for determining the hemolysis index of dendrimers treated RBCs. **B-E**) Represent the toxicity evaluation of dendrimers.** B)** Body weight of the mice before and after the treatment. n = 6. **C)** ALT, AST, CRE, and BUN levels of the mice after the 3 days treatment. n = 6. Repressive images of hematoxylin - eosin staining of liver and kidney in male (**D**) and female (**E**) mice, scale bar=50µm. The data are presented as mean ± S.D. ns, no significance, for toxicity studies. For all the studies, the measurements were done in triplicates for the entire samples with proper control. For the cell viability studies, the p-values were calculated between Control (cells), DMSO (10%), and *2DG-D-Pio* dendrimer at each concentration, with * p < 0.05, ** p < 0.01 and **** p < 0.0001 and ns- non-significant.

*Hemocompatibility studies*. Before *in vivo* efficacy evaluation of the *2DG-D* and *2DG-D-Pio* dendrimers, *ex vivo* hemocompatibility studies were carried out to ensure that the dendrimers were safe to use under *in vivo* settings. From the hemolysis studies, it was observed that at all tested concentrations, the dendrimers were showing excellent compatibility with the rat RBCs. The UV absorption studies suggested no significant peak intensity at 540 nm for all tested concentrations, except for Triton X-100 control, suggesting no signs of hemolysis (**Figure 7A**). Further, the quantitative analysis suggested that the percentage hemolysis at each dendrimer concentration for *2DG-D* was ~4% (5 mg/mL), ~3.7% (2.5 mg/mL), ~4.1% (1.25 mg/mL), ~3.9% (0.63 mg/mL), and ~4.1% (0.31 mg/mL) and for *2DG-D-Pio*, it was, ~4.1% (5 mg/mL), ~4.0% (2.5 mg/mL), ~3.9% (1.25 mg/mL), ~3.8% (0.63 mg/mL), and ~3.9% (0.31 mg/mL). The overall hemolysis was found to be less than 5% for all the samples. Hence, it can be inferred from the results that, there are no toxic and hemolytic effects of *2DG-D* and *2DG-D-Pio* on the RBCs, suggesting safe use of both these dendrimers for *in vivo* efficacy evaluation.

*In vivo biosafety studies.* To further assess the systemic toxic effects of *2DG* and *2DG-D-Pio*, 100 µL of *2DG-D-Pio* (5mg/kg) or equivalent amount of *2DG-D* was administered, while the control group received the same volume of saline. No weight loss was observed after 3 days of treatment (**Figure 7B**). Liver enzymes, including alanine aminotransferase (ALT) and aminotransferase (AST), renal function index, creatinine (CRE), and urea nitrogen (BUN) were tested, and the levels were within the normal range in both the treatment and control groups (**Figure 7C**). In addition, there was no significant damage or difference in liver and kidney sections stained with hematoxylin-eosin after 3 days in the control and dendrimer-treated groups (**Figures 7D and 7E**). These data suggest that dendrimers are not toxic to the liver and kidney of male and female mice.

### 2DG-D-Pio improved behavioral outcomes

**Figure 8:**
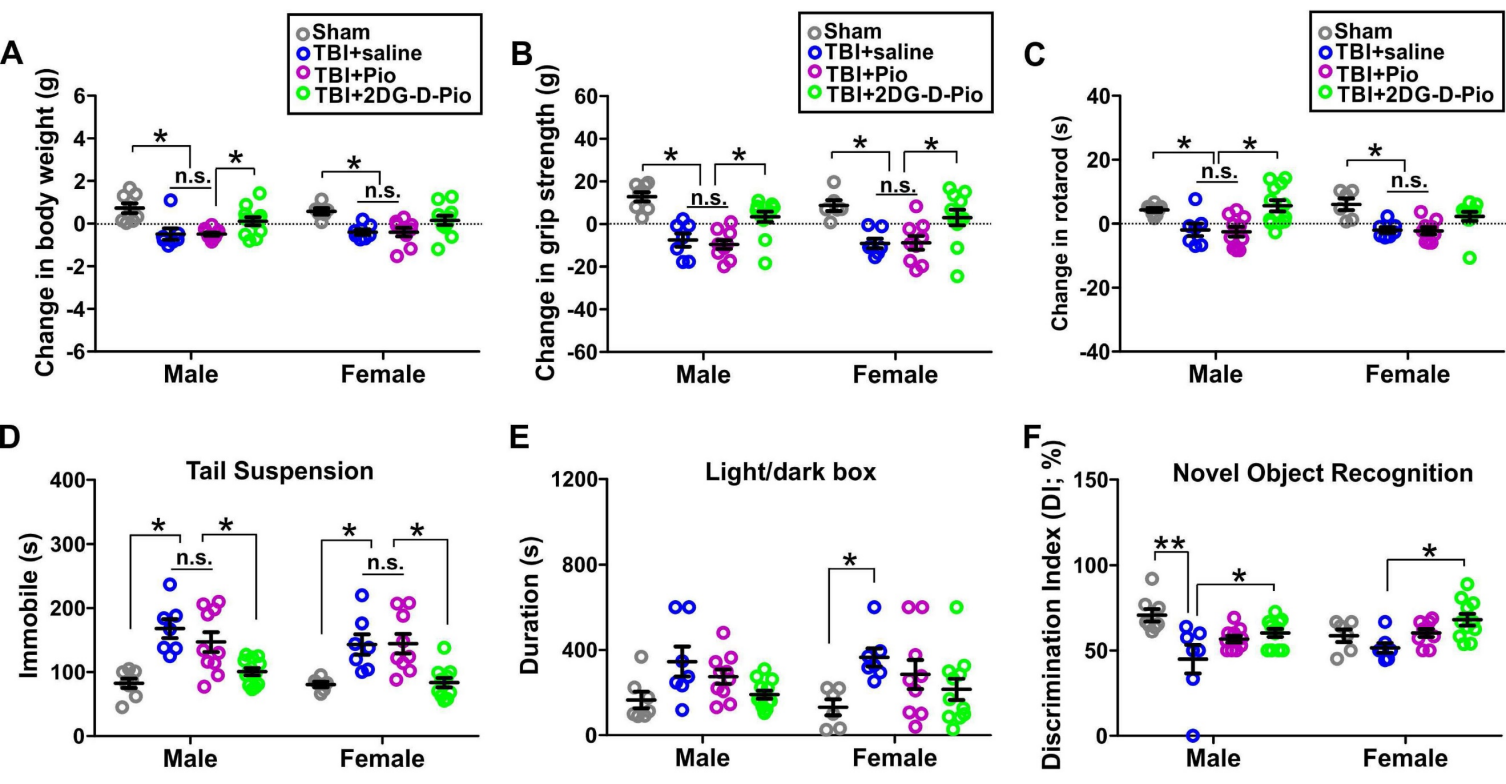
** Evaluation of behavioral outcomes.** Male and female mice littermates from the same litter were randomly divided into sham (n = 14, 8M/6F), TBI+saline (n = 14, 7M/7F), TBI+*Pio* (n = 19, 10M/9F), and TBI+*2DG-D-Pio* (n = 23, 12M/11F) groups. Mice in the TBI groups received free *Pio* (5 mg/kg, 100 μL), *2DG-D-Pio* (containing 5 mg/kg pioglitazone, 100 μL) or PBS (100 μL) at 6-h post-injury. Sham group did not receive any treatment. Behavioral testing were performed at 24-h post-treatment. Data were presented as mean ±SEM. Treatment groups were presented as sham (gray circles), TBI+saline (blue circles), TBI+*Pio* (magenta circles), and TBI+*2DG-D-Pio* (green circles). **A**) The body weight gain significantly decreased in both male TBI+saline and TBI+Pio groups, compared with the male sham group, and in the male TBI+Pio group, compared with male TBI+*2DG-D-Pio* group. **B)** The grip strength significantly decreased in both male and female TBI+saline and TBI+*Pio* groups, compared with the male and female sham and TBI+*2DG-D-Pio* groups. **C)** In the Rotarod test, the latency to the first fall significantly decreased in both male and female TBI+saline and TBI+*Pio* groups, compared with the male and female sham groups. In addition, the latency to the first fall significantly decreased in the male TBI+saline (p < 0.05) and TBI+*Pio* groups, compared with the male TBI+*2DG-D-Pio* group. **D)** The immobile time significantly increased in both male and female TBI+saline and TBI+Pio groups, compared with the male and female sham and TBI+*2DG-D-Pio* groups. **E)** The time spent in the light chamber significantly increased in the female TBI+Pio group, compared with the female sham group. **F)** The time spent with the novel object significantly decreased in the male TBI+saline group, compared with the male sham and TBI+*2DG-D-Pio* groups. The time spent with the novel object significantly decreased in the female TBI+saline group, compared with the female TBI+*2DG-D-Pio* group *, p < 0.05; **, p < 0.01; n.s, no significance.

To evaluate the efficacy of *Pio* and *2DG-D-Pio* on body weight, we compared the change in the body weight before TBI (baseline) and at 1-d post-treatment. Upon two-way ANOVA analysis [sex (male, female), treatment (sham, TBI+saline, TBI+*Pio*, TBI+*2DG-D-Pio*], there were significant differences in the (body weight)_change_ based on treatment [F_(3,62)_=12.58, p < 0.0001]. Specifically, body weight significantly decreased in both male and female TBI+saline and TBI+*Pio* groups, compared with the male (p < 0.01) and female sham groups (p < 0.05). Moreover, body weight significantly decreased in the male TBI+*Pio* group, compared with the TBI+*2DG-D-Pio* group (p < 0.05) (**Figure 8A**). To evaluate the muscle strength and sensorimotor coordination, we compared the changes in the grip strength and rotarod performance before (baseline) and at 1-d post-treatment. For grip strength, upon two-way ANOVA analysis, there were significant differences based on treatment [F_(3, 62)_=19.89, p < 0.0001]. Specifically, grip strength significantly decreased in both male and female TBI+saline (p < 0.001) and TBI+*Pio* (p < 0.05) groups, compared with the male and female sham and TBI+*2DG-D-Pio* groups (**Figure 8B**). For the Rotarod test, upon two-way ANOVA analysis, there were significant differences based on treatment [F_(3, 62)_=12.90, p < 0.0001]. Specifically, the latency to the first fall significantly decreased in both male and female TBI+saline (p < 0.05) and TBI+Pio (p < 0.05) groups, compared with the male and female sham groups. In addition, the latency to the first fall significantly decreased in the male TBI+saline (p < 0.05) and TBI+*Pio* (p < 0.05) groups, compared with the male TBI+*2DG-D-Pio* group (**Figure 8C**). Next, tail suspension and light/dark box tests were used to evaluate the anxiety and depression-like behaviors at 1-d post-treatment. For the tail suspension test, upon two-way ANOVA analysis, there were significant differences based on treatment [F_(3, 62)_=19.27, p < 0.0001]. Specifically, the duration of immobile significantly increased in both male and female TBI+saline (p < 0.01) and TBI+*Pio* (p < 0.01) groups, compared with the male and female sham and TBI+*2DG-D-Pio* groups (**Figure 8D**). For the light/dark box test, upon two-way ANOVA analysis, there were significant differences in the time spent in the light compartment based on treatment [F_(3, 62)_=6.29, p = 0.0009]. Specifically, the duration significantly increased in the female TBI+*Pio* group, compared with the female sham group (p < 0.05) (**Figure 8E**). Novel object recognition test was used to evaluate cognitive function at 1-d post-treatments. Upon two-way ANOVA analysis, there were significant differences in the discrimination index based on treatment [F_(3, 62)_=7.59, p = 0.0002]. Specifically, the mice in the male TBI+saline group spent significantly less time with the novel object, compared with the male sham (p < 0.001) and TBI+*2DG-D-Pio* (p < 0.05) groups. the mice in the female TBI+saline group spent significantly less time with the novel object, compared with the female TBI+*2DG-D-Pio* (p < 0.05) groups (**Figure 8F**).

*In vivo* results demonstrated that *2DG-D-Pio* showed a better efficacy in improving behavioral outcomes, compared with the free drug. For example, the body weight, grip strength and Rotarod performance significantly decreased in both male and female saline-treated and Pio-treated TBI animals, but not in the *2DG-D-Pio* treated group. Moreover, *2DG-D-Pio* treatment significantly improved body weight, grip strength and Rotarod, compared with free *Pio* treatment in males. In females, *2DG-D-Pio* treatment significantly improved grip strength, compared with free *Pio* treatment. In addition, *2DG-D-Pio* treatment significantly decreased immobile time during tail suspension test, compared with the TBI+saline and TBI+*Pio* groups in both males and females. Whereas *Pio*-treated TBI animals did not show significant improvement.

### 2DG-D-Pio improved neuroinflammatory responses

We first evaluated the effects of *2DG-D* alone on neuroinflammation and cell death. TBI mice received intraperitoneal injection of *2DG-D* (100 μL) or saline (100 μL) at 6-h post-injury. Animals were euthanized at 24-h post-treatment. The neurons from the injured brain regions were isolated for gene expression evaluation. We found that there was no significant difference in the expression of neuroinflammatory and cell death markers between the TBI+saline and TBI+*2DG-D* groups (**Supplemental Figure S45**). These data indicate that *2DG-D* alone did not have significant effect on neuroinflammation and cell death. Next, we evaluated the efficacy of *Pio* and *2DG-D-Pio* on neuroinflammatory responses using brain tissues from the injured brain regions (or the matching areas in the sham), which included all brain cell types (e.g. neurons, microglia, astrocytes, etc.). We found that *2DG-D-Pio* significantly improved neuroinflammatory responses, compared with the TBI+saline mice. *2DG-D-Pio* also showed a better efficacy than free *Pio* but did not reach statistical significance (**Supplemental Figure S46**).

**Figure 9:**
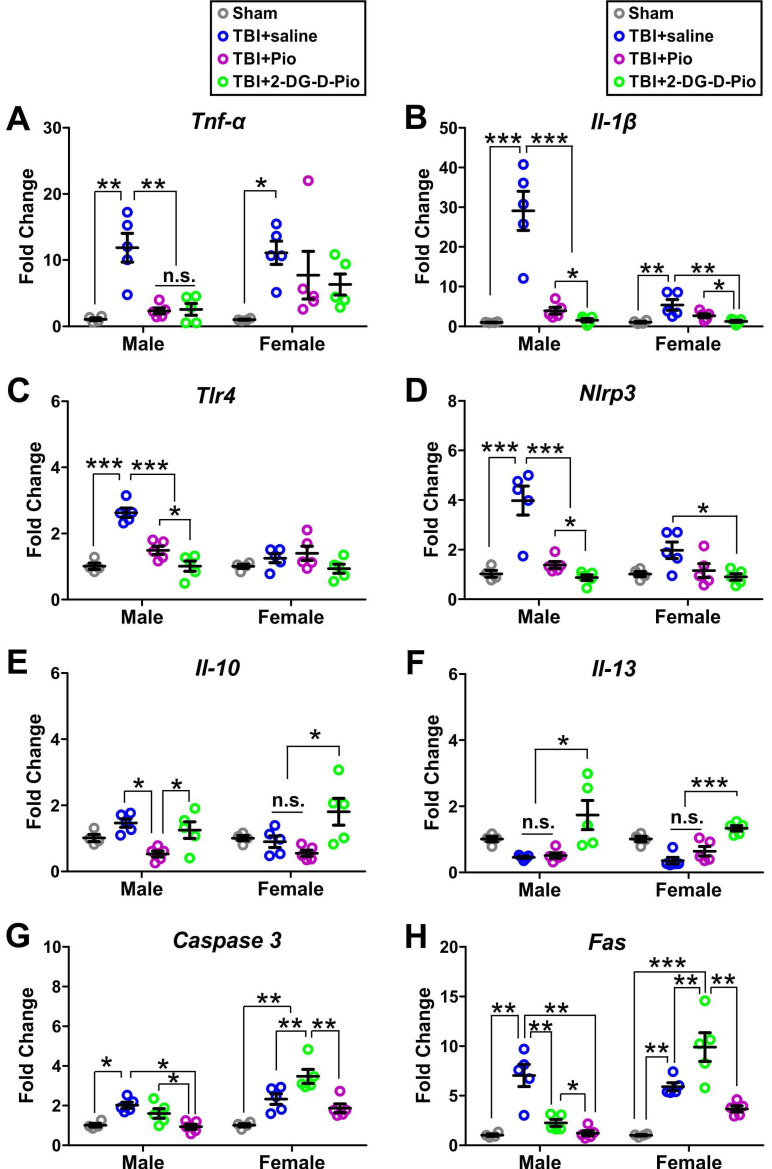
mRNA expressions of pro- and anti-inflammatory markers and cell death markers from sham (n = 8, 4M/4F), TBI+saline (n = 10, 5M/5F), TBI+*Pio* (n = 10, 5M/5F), and TBI+*2DG-D-Pio* (n = 10, 5M/5F) groups. Neurons were isolated from the injured brain regions (or the matching area from the sham mice) at 24-h post-treatment for gene expression evaluations. Data were presented as mean ±SEM. Treatment groups were presented as sham (gray circles), TBI+saline (blue circles), TBI+*Pio* (magenta circles), and TBI+*2DG-D-Pio* (green circles). **A-D)** The expression of pro-inflammatory makers, TNF-α (A), IL-1β (B), TLR4 (C), and NLRP3 (D). **E-F)** The expression of anti-inflammatory makers IL-10 (E) and IL-13 (F). **G-H)** The expression of cell death makers caspase-3 (G) and Fas (H). *, p < 0.05; **, p < 0.01; ***, p < 0.001; n.s., no significance.

To evaluate the effect of treatment on neurons, we isolated primary neurons from the injured brain regions of TBI animals (or the matching areas in the sham) after treatment to further evaluate the efficacy of *Pio* and *2DG-D-Pio* on neuro-inflammatory responses and cell death specifically in neurons. We first compared the mRNA expression of pro-inflammatory markers (TNF-α, IL-1β, TLR4, and NLRP3) at 1-d post-treatments. The expression of these pro-inflammatory markers was significantly higher in saline treated TBI animals compared to sham controls. The TNF-α expression significantly decreased in TBI male mice treated with free *Pio* (p < 0.05) or *2DG-D-Pio* (p < 0.05) compared to saline-treated TBI animals (**Figure 9A**). More specifically, upon two-way ANOVA analysis [sex (male, female), treatment (sham, TBI+saline, TBI+*Pio*, TBI+*2DG-D-Pio*)], there were significant differences in the TNF-α expression based on treatment [F_(3, 30)_=10.84, p < 0.0001]. Specifically, TNF-α expression significantly increased in both male (p < 0.01) and female (p < 0.01) TBI+saline group, compared with the male and female sham groups. Moreover, TNF-α expression significantly increased in the male TBI+saline group, compared with the male TBI+*Pio* (p < 0.05) and TBI+*2DG-D-Pio* (p < 0.05) groups (**Figure 9A**).

We further evaluated the IL-1 β expression. IL-1β increases early following experimental and human TBI, and is closely associated with injury severity 67. It stimulates glutamate excitotoxicity and promotes cell loss 68, while neutralization of IL-1β reduces neuronal death and improves cognitive outcome after TBI 69, 70. There were significant differences in the IL-1β expression based on treatment [F_(3, 30)_=31.66, p < 0.0001], sex [F_(3, 30)_=20.31, p < 0.0001], and the interaction (treatment x sex) [F_(3, 30)_=17.99, p < 0.0001]. In males, IL-1β expression significantly increased in the TBI+saline group, compared with sham (p < 0.0001), TBI+*Pio* (p < 0.0001) and TBI+*2DG-D-Pio* (p < 0.0001) groups. Moreover, IL-1β expression significantly increased in the TBI+*Pio* group, compared with the TBI+*2DG-D-Pio* group (p < 0.05). In females, IL-1β expression significantly decreased in *2DG-D-Pio* treated TBI animals compared to saline-treated animals (p < 0.001) groups and this reduction was significantly more in *2DG-D-Pio* group when comparted to the free *Pio* group (p < 0.05) (**Figure 9B**), suggesting the effect of neuron targeted delivery of *Pio via 2DG-D-Pio*.

We next examined the effect on TLR4 expression. There were significant differences in the TLR4 expression based on treatment [F_(3, 30)_=19.4, p < 0.0001], sex [F_(3, 30)_=13.87, p = 0.0008] and the interaction (treatment x sex) [F_(3, 30)_=10.44, p < 0.0001]. Specifically, TLR4 expression significantly increased in the male TBI+saline group, compared with the male sham (p < 0.0001). The TLR4 expression decreased significantly in both TBI+*Pio* (p < 0.0001), and TBI+*2DG-D-Pio* (p < 0.0001) groups compared to saline treated TBI males. The dendrimer conjugate *2DG-D-Pio* had a better effect in reducing the TLR4 expression compared to free *Pio* (p < 0.05) groups (**Figure 9C**). There were significant differences in the NLRP3 expression based on treatment [F_(3, 30)_=23.21, p < 0.0001], sex [F_(3, 30)_=7.2, p = 0.0117] and the interaction (treatment x sex) [F_(3, 30)_=5.8, p = 0.0030]. A similar trend to TLR4 expression was found in NLRP3 expression in males. However, the *2DG-D-Pio* treated female TBI animals showed significantly reduced NLRP3 expression compared to the TBI+*Pio* (p < 0.05) group (**Figure 9D**).

Next, we compared the mRNA expression of anti-inflammatory markers (IL-10 and IL-13) at 1-d post-treatments. IL-10 is an anti-inflammatory cytokine that can inhibit the expression of pro-inflammatory factors and mediate the recovery process following TBI 71. Moreover, IL-10 can prevent prolonged secondary brain damage by facilitating cytokine storm resolution 72. Upon two-way ANOVA analysis, there were significant differences in the IL-10 expression based on treatment [F_(3, 30)_=8.33, p = 0.0004]. Specifically, in males, IL-10 expression significantly decreased in the TBI+*Pio* group, compared with the TBI+saline (p < 0.05) and TBI+*2DG-D-Pio* (p < 0.01) groups. In females, IL-10 expression significantly increased in the TBI+*2DG-D-Pio* group, compared with the TBI+saline (p < 0.05) and TBI+*Pio* (p < 0.01) groups (**Figure 9E**). We next examined the IL-13 expression. IL-13, an anti-inflammatory cytokine, plays an important role in learning and memory 73. IL-13 is expressed in neurons and can modulate neuronal activity and synaptic plasticity 73. Studies have shown that IL-13 protects neurons against excitotoxic insults after TBI 22. There were significant differences in the IL-13 expression based on treatment [F_(3, 30)_=15.41, p < 0.0001]. IL-13 expression significantly increased in both male (p < 0.05) and female (p < 0.001) TBI+*2DG-D-Pio* groups, compared with the male and female TBI+saline and TBI+*Pio* groups (**Figure 9F**). In our previous studies, we have demonstrated that pediatric TBI causes aberrant neuro-inflammatory responses, abnormal protein accumulation, and cell loss, resulting in impaired sensorimotor function, cognitive deficits, and depressive behaviors 48-50, 53, 74, 75. Therefore, the decreased IL-1β and increased IL-10 and IL-13 after *2DG-D-Pio* treatment might rescue neuronal loss and be responsible for the improved neuro-behaviors.

We further compared the mRNA expression of cell death markers (Caspase-3 and Fas) at 1-d post-treatments. Upon two-way ANOVA analysis, there were significant differences in the caspase-3 expression based on treatment [F_(3, 30)_=19.08, p < 0.0001], sex [F_(3, 30)_=24.27, p < 0.0001], and the interaction (treatment x sex) [F_(3, 30)_=6.80, p = 0.0012]. In both males and females, the Caspase 3 expression significantly increased in saline treated TBI animals compared to sham controls. In males, both free *Pio* and *2DG-D-Pio* treatment decreased the Caspase 3 expression compared to saline-treated group, however, the expression significantly decreased in the TBI+*2DG-D-Pio* group compared to the free *Pio* group (p < 0.05). In females, while *2DG-D-Pio* treatment significantly lowered the Caspase 3 expression compared to the saline-treated animals, the treatment with free *Pio* showed an increase in the expression of Caspase 3 (**Figure 9G**). We further evaluated the Fas expression, another cell death marker. There were significant differences in the Fas expression based on treatment [F_(3, 30)_=26.24, p < 0.0001], sex [F_(3, 30)_=18.66, p = 0.0002], and the interaction (treatment x sex) [F_(3, 30)_=14.72, p < 0.0001]. Similar to Caspase 3, the Fas expression was significantly higher in saline treated TBI animals compared to sham controls irrespective of the sex. In males, both dendrimer conjugate and free drug treated animals showed significantly reduced Fas expression compared to saline treated animals, however, the reduction was significantly more in the *2DG-D-Pio* group compared to the free *Pio* treated animals. Like the trend seen in females for Caspase 3 expression, the free *Pio* treated animals demonstrated significantly higher Fas expression compared to sham control, saline-treated, and *2DG-D-Pio* treated animals. In females, Fas expression significantly increased in the TBI+*Pio* group, compared with the TBI+saline (p < 0.01) and TBI+*2DG-D-Pio* (p < 0.01) groups (**Figure 9H**).

Previous studies have shown that *Pio* has neuroprotective and anti-inflammatory effects after TBI 36. For example, in a controlled cortical impact (CCI) model of TBI, *Pio* treatment at 15 min post-injury exhibits neuroprotective function *via* activating PPARγ and reducing NF-κB and IL-6 76. In the present study, we chose the 6 h time point to reflect the clinical treatment time for TBI patients 77, which provides the basis for assessment of therapeutic time window of the novel dendrimer platform. Interestingly, there are sex differences in the behavioral outcomes and the expression of inflammatory and cell death markers among treatment groups. Studies have shown that there are sex differences in the pharmacokinetics of *Pio*, and the efficacy of *Pio* treatment in nonalcoholic fatty liver disease is also gender dependent 78, 79. Moreover, growing evidence indicates that TBI alone can induce sex-specific neuroinflammatory responses 48-50, 53, 74, 75, and behavioral outcomes, depending on differential cellular responses, sex hormones, and metabolism 80, 81. Therefore, the sex differences in behaviors and neuroinflammatory responses can be caused by the combined effects of brain injury and pioglitazone treatment, however, the underlying mechanisms need to be further investigated.

## Conclusions

The primary challenge in treating brain disorders has long been the major hurdle in drug delivery across the BBB. Even if drugs or nanoparticles manage to traverse the compromised BBB after brain injury or neuroinflammation, effectively reaching key cells involved in brain diseases, such as neurons, remains a significant hurdle. Typically, nanocarriers are modified with targeting ligands to facilitate delivery across the BBB to specific cell types. However, this post-synthetic functionalization process is often time-consuming and meticulous, posing challenges related to batch-to-batch reproducibility and other variabilities. The distinctive feature of *2DG-D* is its straightforward synthesis and inherent capacity to target and localize within neurons precisely at the site of brain injury. We have successfully demonstrated the delivery of a neuroprotective drug, pioglitazone, using *2DG-D*, resulting in improved behavioral outcomes and neuroinflammatory responses in a pediatric mouse model of TBI. These promising *in vitro* and *in vivo* results coupled with a simple approach for the construction of *2DG-D* makes it a potential nanoplatform for addressing brain diseases.

## Supplementary Material

Details about synthetic materials and methods, in vitro drug release studies, formulation stability studies, and supplementary figures.

## Figures and Tables

**Table 1:** Primers for qPCR

Gene	Forward primer	Reverse Primer
*Tnf-α*	TCAGCCGATTTGCTATCTC ATA	AGTACTTGGGCAGATTGACCTC
*Il-1β*	GGTGTGTGACGTTCCCATTA	ATTGAGGTGGAGAGCTTTCAG
*Il-4*	GACGGCACAGAGCTATTGAT	GGATATGGCTCCTGGTACATTC
*Il-6*	GTCTGTAGCTCATTCTGCTCTG	GAAGGCAACTGGATGGAAGT
*Il-10*	TTGAATTCCCTGGGTGAGAAG	TCCACTGCCTTGCTCTTATTT
*Il-13*	GCTGAGCAACATCACACAAG	AATCCAGGGCTACACAGAAC
*Tgf-β1*	GGTGGTATACTGAGACACCTTG	CCCAAGGAAAGGTAGGTGATAG
*iNOS*	GGAATCTTGGAGCGAGTTGT	CCTCTTGTCTTTGACCCAGTAG
*Nlrp3*	ACGTGTTCCAGAAGGAAGTG	GCCTCCTCTTCCAGCAAATA
*Tlr4*	GGGTATTTGACACCCTCCATAG	CAAGAGTGCTGAGGGAATACAG
*Gapdh*	AACAGCAACTCCCACTCTTC	CCTGTTGCTGTAGCCGTATT

## Abbreviations

BBB: blood brain barrier
cDNA: single-stranded complementary DNA
CuAAC: copper (I) catalyzed click chemistry
2-DG: 2-deoxy-glucose
2DG-D: 2-deoxy-glucose dendrimer
DMF: *N*, *N*-dimethylformamide
Gapdh: glyceraldehyde 3-phosphate dehydrogenase
GRAS: generally recognized as safe
IACUC: Institutional Animal Care and Use Committee
IL-1β: interleukin-1 beta
MW: microwave
iNOS: inducible nitric oxide synthase
Pio: pioglitazone
1NLRP3: NLR family pyrin domain containing 3
PEG: polyethylene glycol
RT: room temperature
TBI: traumatic brain injury
TGF-β1: transforming growth factor beta
TLR4: Toll-like receptor 4
and TNF-α: tumor necrosis factor alpha
