# Supplementary material for: Discovery of 2-deoxy glucose surfaced mixed layer dendrimer: a smart neuron targeted systemic drug delivery system for brain diseases
Source: Theranostics. 2024 May 19;14(8):3221–45. doi: 10.7150/thno.95476 (PMC11155412; doi:10.7150/thno.95476)
Supplement: Supplementary file 1 — Details about synthetic materials and methods, in vitro drug release studies, formulation stability studies, and supplementary figures. [file thnov14p3221s1.pdf]

Supporting Information

Discovery of 2-deoxy glucose surfaced mixed layer dendrimer: a smart neuron targeted systemic drug delivery system for brain diseases

Anubhav Dhull[#1], Zhi Zhang[#2], Rishi Sharma[#1], Aqib Iqbal Dar[1], Anu Rani[1], Jing Wei[3], Shamila Gopalakrishnan[1], Amanda Ghannam[2], Victoria Hahn[2], Anunay James Pulukuri[1], Stefanie Tasevski[2], Sara Moughni[2], Boyang Jason Wu[3], Anjali Sharma[1]*

[1]*Department of Chemistry, College of Arts and Sciences, Washington State University, 1470 NE College Ave, Pullman, WA, USA 99164.*

[2]*Department of Natural Sciences, College of Arts, Sciences, and Letters, University of Michigan - Dearborn, 4901 Evergreen Rd, Dearborn, MI, USA 48128.*

[3]*Department of Pharmaceutical Sciences, College of Pharmacy and Pharmaceutical Sciences, Washington State University, Spokane, WA, USA 99202.*

*Correspondence: Anjali Sharma, Department of Chemistry, Washington State University, Troy Hall, Rm 222, 1470 E. College Avenue, Pullman, WA 99164, USA.

E-mail: anjali.sharma@wsu.edu

[#]These authors contributed equally to this work.

Contents:

1. Chemistry-Experimental Section

1.1 Materials

1.2 Instrumentation

1.3 Synthesis protocols

1.4 In vitro drug release studies

1.5 2DG-D-Pio formulation stability studies

2. Supplementary Figures

1. Chemistry-Experimental Section:

1.1 Materials.

All starting materials and reagents were purchased from Sigma-Aldrich and Merck. Commercially available reagents and solvents for synthesis were analytical grade and used without further purification. Thin-layer chromatography was performed on a film of silica gel that contained a fluorescent indicator F_{254} supported on an aluminum sheet (Merck). Spectra/Por dialysis membranes were purchased from Repligen.

1.2 Instrumentation.

Nuclear Magnetic Resonance (NMR) spectra of samples were recorded on a Bruker spectrometer operating at 500 MHz at 25 °C. The samples were prepared in deuterated chloroform ($CDCl_3$), deuterated DMSO (DMSO-d_6) or deuterated water (D_2O).

Microwave reactions were performed in a Biotage Initiator+ instrument using a sealed 10 mL/ 20 mL process vials. Reaction times refer to irradiation time at the target temperature, not the total irradiation time. The temperature was measured with an IR sensor.

The purity and drug release studies were analyzed using high-performance liquid chromatography (HPLC). The HPLC was performed using a Waters Acquity Arc system (Milford, MA, USA), equipped with binary pumps, 2998 PDA detector, and a 2475 fluorescence detector. The analyses were performed using Waters Empower software. The samples were run using Waters C18 symmetry 300, 5 μm, 4.6 × 250 mm column using a gradient flow method starting with 90:10 (Solvent A: 0.1% TFA and 5% ACN in water; Solvent B: 0.1% TFA in ACN), gradually increasing to 50:50 (A:B) at 20 minutes, 10:90 (A:B) at 38 minutes finally returning to 90:10 (A:B) at 40 minutes. A flow rate of 1 mL/min was maintained during the run. The dendrimers and drug conjugates were detected at 210 and 254 nm. The 2DG-D-Cy5 was detected at 650nm.

The particle size and zeta potential distribution were determined by dynamic light scattering (DLS) using a Malvern Zetasizer Nano 90 (Westborough, MA). To measure the size, the 2DG-D was dissolved in deionized water (18.2 Ω) to create a solution with a final concentration of 0.5 mg/mL. This solution was then passed through 0.2 μm syringe filters (Pall Corporation, 0.2 μm HT Tuffryn

membrane) directly into the cell (UV transparent disposable cuvette, dimensions: 12.5 × 12.5 × 45 mm). For zeta potential measurement, a sample at a concentration of 0.2 mg/mL in 10 mM NaCl was prepared using the same procedure. The measurements were conducted using a Malvern Zetasizer Nanoseries disposable folded capillary cell.

High resolution mass spectra were performed by Dr. Yue Li in the department of Chemistry and Biochemistry at the University of Maryland–College Park using ESI by direct infusion on a Bruker MALDI-TOF (trans-2-[3-(4-tert-Butylphenyl)-2-methyl-2-propenylidene]malononitrile matrix), and Bruker Q-TOF.

1.3 Synthesis protocols

Synthesis of intermediates and dendrimers

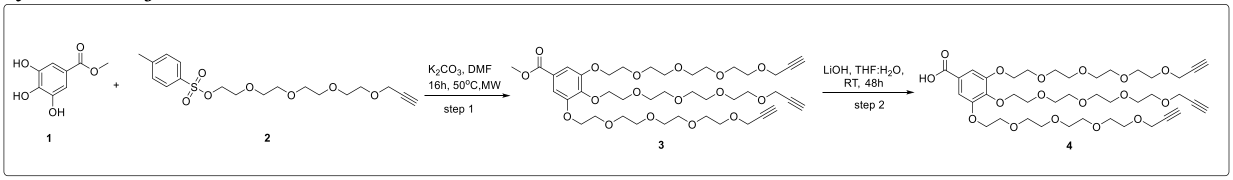

Synthesis of methyl 3,4,5-tris((3,6,9,12-tetraoxapentadec-14-yn-1-yl)oxy)benzoate (3): Compound **1** (600 mg, 1.0 eq, 3.26 mmol) and K_2CO_3 (3.6 g, 26 mmol, 8.0 eq) was dissolved in a 5 mL of DMF in a microwave glass vial under inert atmosphere. After 10 minutes, solution of Compound **2** (5g, 4.0 eq, 13 mmol) in 7.5 mL DMF was added dropwise to the mixture. The reaction vial was irradiated to 50 ^{o}C for 16 hours in microwave synthesizer. After completion of the reaction as evident from TLC, DCM was added to reaction mixture and filtered to remove K_2CO_3. Filtrate was washed with water (3 × 50 mL) and chilled brine (2 × 50 ml), dried over Na_2SO_4, and evaporated in vacuo. The crude product was purified by silica flash column chromatography [methanol/dichloromethane, 4:96 (v/v)] to afford compound **3** in 60% yield.

Brown viscous liquid; ^{1}H NMR (500 MHz, $CDCl_3$) δ 2.43 (s, 3H), 3.63-3.72 (m, 35H), 3.78 (t, J = 5.2 Hz, 3H), 3.84-3.90 (m, 7H), 4.17-4.25 (m, 12H), 7.29 (s, 2H). ^{13}C NMR (125 MHz, DMSO-d_6) δ 52.65, 57.94, 60.67, 68.97, 68.99, 69.39, 69.96, 70.22, 70.25, 70.30, 70.32, 70.38, 70.41, 72.40, 72.81, 77.55, 77.59, 80.79, 108.54, 124.82, 142.35, 152.48, 166.28. (MALDI-TOF) *m/z*: calculated for $C_{41}H_{62}O_{17}$ $[M+Na]^+$: 849.3885; found 849.3870.

Synthesis of 3,4,5-tris(2-(2-(2-(2-hydroxyethoxy)ethoxy)ethoxy)ethoxy)benzoic acid (4): To stirred solution of Compound **3** (1 g, 1.0 eq, 1.21 mmol) in 5 mL THF:H_2O (4:1) was added

solution of LiOH.H_2O (290 mg, 10 eq, 12.1 mmol) 1 mL DI water. The reaction mixture was stirred at room temperature for 48h until complete conversion was achieved. The reaction mixture was diluted with water (150 mL), acidified (pH 2-4) with dropwise addition of 1N HCl and extracted with DCM (2 ×100 mL). The combined organic layer was washed brine (50 mL), then dried (Na_2SO_4), filtered, and evaporated in vacuo. The crude product was purified by silica flash column chromatography [methanol/dichloromethane, 6:94 (v/v)] to afford compound **4** in 95% yield.

Brown viscous liquid; ^{1}H NMR (500 MHz, $CDCl_3$) δ 2.31 (m, 3H), 3.49 – 3.59 (m, 35H), 3.67 (t, *J* = 5.1 Hz, 3H), 3.73 (t, *J* = 5.1 Hz, 4H), 4.05 – 4.14 (m, 12H), 7.14 (s, 2H). ^{13}C NMR (125 MHz, $CDCl_3$) δ 58.39, 58.41, 68.93, 69.09, 69.10, 69.70, 70.38, 70.40, 70.55, 70.58, 70.59, 70.63, 70.67, 70.68, 70.70, 70.86, 72.46, 74.61, 74.65, 79.63, 79.68, 109.74, 124.18, 143.20, 152.33, 169.93. (MALDI-TOF) *m/z*: calculated for $C_{40}H_{60}O_{17}$ [M-H]$^+$: 811.3752; found 811.3752.

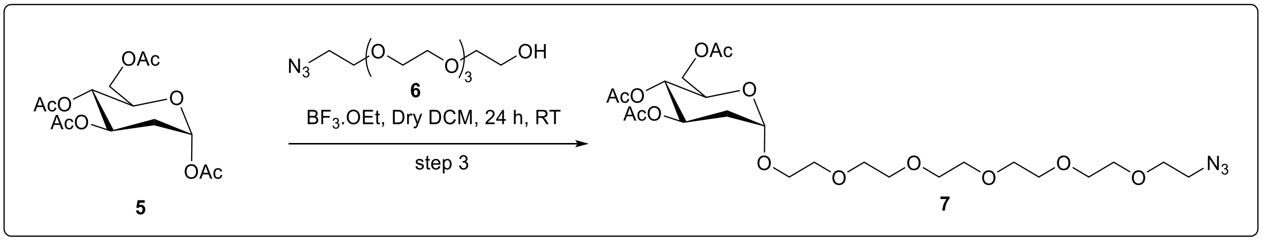

Synthesis of (2R,3S,4R,6S)-2-(acetoxymethyl)-6-((17-azido-3,6,9,12,15-pentaoxaheptadecyl) oxy)tetrahydro-2H-pyran-3,4-diyl diacetate (7): Compound **5** (1.27 g, 1.0 eq, 3.82 mmol) was dried on high vacuum for 5 minutes, dissolved in 10 mL anhydrous DCM and the solution was stirred at 0°C . To this stirred solution was added boron trifluoride etherate (1.18 mL, 2.5 eq, 9.60 mmol) dropwise at 0°C followed by drop wise addition of Azido-PEG6-Alcohol **6** (1.27 g, 1.0 eq, 3.82 mmol). The reaction mixture was allowed to stir at room temperature for 24h. The reaction was constantly monitored with the help of thin-layer chromatography (TLC) stained with H_2SO_4 dip. After maximum conversion on TLC, the reaction was quenched with ice. The reaction mixture was diluted with DCM and the organic layer was washed with saturated sodium bicarbonate solution (2 × 50 ml) and brine (2 × 50 ml). The combined organic layer was dried over Na_2SO_4, filtered, and evaporated in vacuo. The crude product was purified by silica flash column chromatography [ethyl acetate/hexane, 90:10 (v/v)] to afford compound **7** in 50% yield.

Viscous liquid; ^{1}H NMR (500 MHz, $CDCl_3$) δ 1.82 (td, J = 12.47, 3.47 Hz, 1H), 1.98-2.11 (m, 9H), 2.26 (dd, *J* = 13.04, 5.38 Hz, 1H), 3.39 (t, *J* = 5.23 Hz, 2H), 3.58-3.77 (m, 22H), 4.00-4.05 (m, 2H),

4.31 (dd, J = 12.56, 4.35 Hz, 1H), 4.98-5.02 (m, 2H), 5.30-5.36 (m, 1H). ^{13}C NMR (125 MHz, CDCl3) δ 20.77, 20.80, 21.01, 34.94, 50.68, 62.31, 66.84, 67.74, 69.08, 69.36, 70.04, 70.16, 70.58, 70.60, 70.63, 70.66, 70.68, 70.70, 77.23, 97.10, 169.96, 170.23, 170.81. (MALDI-TOF) m/z: calculated for $C_{24}H_{41}N_3O_{13}$ $[M+Na]^+$ 602.2537 found 602.2526.

Specific rotation: (α_D^{20}= 53.8° (c= 1, $CHCl_3$).

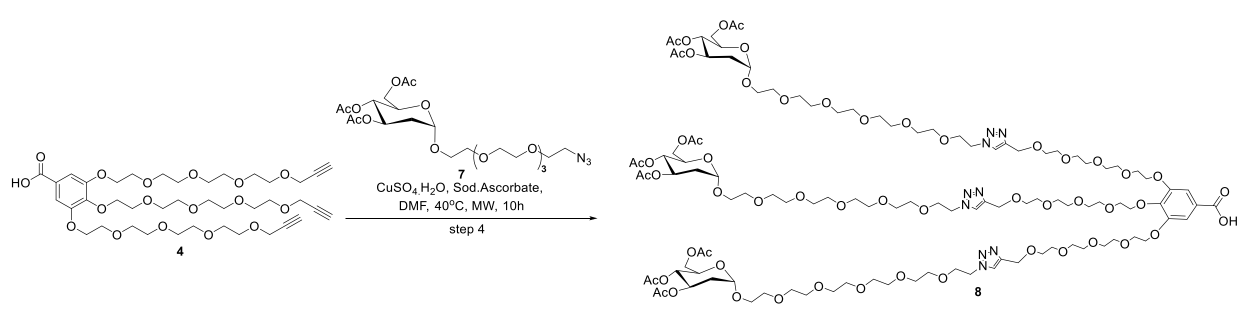

Synthesis of 3,4,5-tris((1-(1-(17-(((2S,4R,5S,6R)-4,5-diacetoxy-6-(acetoxymethyl)tetrahydro-2H-pyran-2-yl)oxy)-3,6,9,12,15-pentaoxaheptadecyl)-1H-1,2,3-triazol-4-yl)-2,5,8,11-tetraoxatridecan-13-yl)oxy)benzoic acid (8): To a stirred solution of compound **4** (280 mg, 1.0 eq, 0.344 mmol) in 4 mL DMF in a microwave vial was added solution of compound **7** (678 mg, 3.5 eq, 1.171 mmol) dissolved in 4 mL DMF followed by the addition of $CuSO_4.5H_2O$ (5mol% per acetylene) dissolved in 1mL DI water. After 2 minutes, Sodium Ascorbate (10 mol% per acetylene) was added, and the reaction was performed under microwave irradiation at 40°C for 10h. Progress of the reaction was monitored with thin-layer chromatography (TLC). After complete conversion, the reaction mixture was diluted with DCM (100 mL) and washed with saturated EDTA solution (7 × 30 ml) and brine (1 × 50 ml), then dried (Na_2SO_4), filtered, and evaporated in vacuo. The crude product was purified by silica flash column chromatography [methanol/dichloromethane, 8:92 (v/v)] to afford compound **8** in 90% yield.

Viscous Liquid; ^{1}H NMR (500 MHz, $CDCl_3$) δ 1.80 (td, J = 12.1, 3.6 Hz, 3H), 1.96 – 2.03 (m, 18H), 2.06 (s, 9H), 2.24 (dd, J = 13.0, 5.4 Hz, 3H), 3.56 – 3.66 (m, 97H), 3.68 – 3.87 (m, 17H), 3.97 – 4.05 (m, 5H), 4.14 – 4.24 (m, 7H), 4.29 (dd, J = 12.5, 4.3 Hz, 3H), 4.48 – 4.54 (m, 6H), 4.66 (d, J = 12.6 Hz, 6H), 4.94 – 5.01 (m, 6H), 5.30 (ddd, J = 12.2, 9.5, 5.2 Hz, 3H), 7.36 (s, 2H), 7.69 – 7.75 (m, 3H). ^{13}C NMR (125 MHz, $CDCl_3$) δ 19.46, 19.76, 19.80, 19.82, 19.93, 20.00, 28.69, 28.94, 33.91, 35.10, 52.46, 61.30, 61.43, 61.51, 62.57, 63.06, 65.81, 66.05, 66.73, 67.70,

67.91, 68.06, 68.10, 68.33, 68.78, 69.14, 69.31, 69.44, 69.47, 69.52, 69.56, 69.61, 69.64, 69.73, 69.87, 70.93, 71.32, 76.26, 96.07, 97.60, 98.72, 108.61, 141.48, 151.18, 166.39, 168.94, 169.24, 169.81. (MALDI-TOF) *m/z*: calculated for $C_{112}H_{183}N_9O_{56}$ 2550.17 found 2550.18.

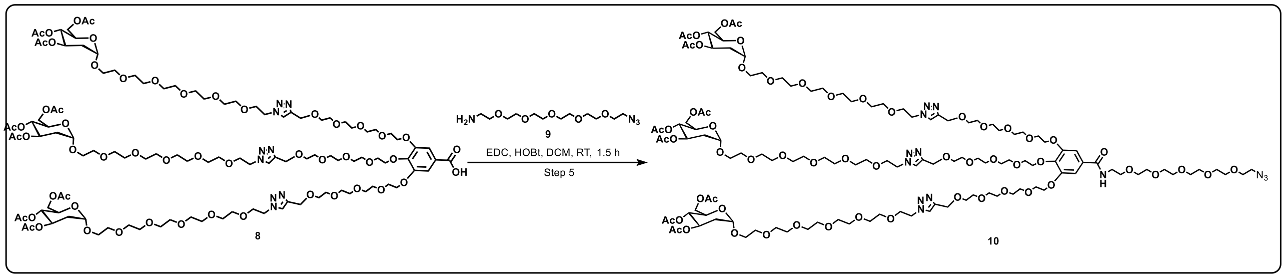

Synthesis of compound 10: Compound **8** (200 mg, 1.0 eq, 0.078 mmol) was dissolved in 4mL Anhy. DCM and stirred at 0°C. EDC.HCl (22.54 mg, 1.5 eq, 0.1176 mmol) and HOBT (16.72 mg, 1.4 eq, 0.109 mmol) were added to the reaction mixture and stirred for 40 minutes. Compound **9** (47.55 mg, 2.0 eq, 0.157 mmol) was dissolved in 1mL Anhy. DCM and was added to the reaction mixture. The Reaction mixture was allowed to stir at room temperature for 1.5 hours. Progress of reaction was monitored with the help of thin-layer chromatography (TLC) in UV light. The reaction mixture was diluted with DCM and the organic layer was washed with water (5 × 30 ml) and brine (2 × 30 ml), then dried (Na_2SO_4), filtered, and evaporated in vacuo. The crude product was purified by silica flash column chromatography [methanol/dichloromethane, 10:90 (v/v)] to afford compound **10** in 90% yield.

^{1}H NMR (500 MHz, DMSO-d$_6$) δ 1.72-1.83 (m, 3H), 1.93-2.05 (m, 27H), 2.06-2.14 (m, 3H), 3.35-3.43 (m, 5H), 3.43-3.74 (m, 127H), 3.72-3.89 (m, 7H), 3.90-4.08 (m, 5H), 4.08-4.22 (m, 7H), 4.43-4.63 (m, 11H), 4.80-4.90 (m, 2H), 4.96-5.02 (m, 2H), 5.07-5.20 (m, 2H), 7.19 (d, *J* = 7.4 Hz, 2H), 7.97-8.12 (m, 3H), 8.46 (t, *J* = 5.7 Hz, 1H, NH). ^{13}C NMR (125 MHz, DMSO-d$_6$) δ ^{13}C NMR (126 MHz, DMSO) δ 20.90, 20.99, 21.11, 34.94, 49.75, 50.43, 55.40, 62.49, 63.97, 66.57, 67.70, 68.80, 68.96, 69.17, 69.36, 69.40, 69.50, 69.71, 69.82, 69.98, 70.07, 70.14, 70.21, 70.24, 70.26, 70.30, 70.41, 72.31, 96.60, 106.71, 106.72, 124.70, 144.24, 152.23, 165.99, 169.95, 170.21, 170.56.

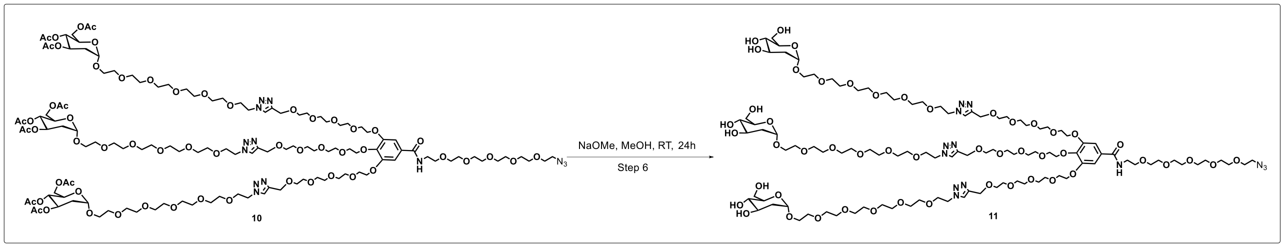

Synthesis of N-(17-azido-3,6,9,12,15-pentaoxaheptadecyl)-3,5-bis((1-(1-(17-(((2S,4R,5S,6R)-4,5-dihydroxy-6-(hydroxymethyl)tetrahydro-2H-pyran-2-yl)oxy)-3,6,9,12,15-pentaoxaheptadecyl)-1H-1,2,3-triazol-4-yl)-2,5,8,11-tetraoxatridecan-13-yl)oxy)-4-((1-(1-(17-(((2S,5S,6R)-4,5-dihydroxy-6-(hydroxymethyl)tetrahydro-2H-pyran-2-yl)oxy)-3,6,9,12,15-pentaoxaheptadecyl)-1H-1,2,3-triazol-4-yl)-2,5,8,11-tetraoxatridecan-13-yl)oxy)benzamide (11): To a stirred solution of compound **10** (1g, 1.0 eq, 0.347mmol) dissolved in dry MeOH (10mL), sodium methoxide (300 mL, 1.1 M solution in MeOH) was added dropwise to adjust the pH around 8.5-9. The reaction mixture was stirred overnight at room temperature. The completion of the reaction was monitored by TLC. On completion, the pH was adjusted between 6 to7 with an Amberlist IR-120 resin. The resin was removed by filtration, and the solvent was removed by rotary evaporation to yield compound **11** in 89%.

Viscous Liquid; ^{1}H NMR (500 MHz, DMSO-d$_6$) δ 1.39 – 1.47 (m, 3H), 1.84 – 1.93 (m, 3H), 2.98 – 3.06 (m, 3H), 3.17 (d, J = 5.0 Hz, 2H), 3.33 – 3.70 (m, 133H), 3.75 – 3.82 (m, 9H), 4.04 – 4.16 (m, 6H), 4.42 (t, J = 5.9 Hz, 3H), 4.45 – 4.59 (m, 12H), 4.73 (d, J = 4.9 Hz, 3H), 4.82 (d, J = 3.4 Hz, 3H), 4.85 (d, J = 5.1 Hz, 3H), 7.19 (s, 2H), 8.05 (s, 3H), 8.46 (t, J = 5.7 Hz, 1H). ^{13}C NMR (125 MHz, DMSO-d$_6$) δ 38.31, 40.35, 49.76, 50.43, 61.47, 63.94, 66.04, 67.82, 68.41, 68.80, 69.15, 69.37, 69.40, 69.47, 69.70, 69.98, 70.00, 70.06, 70.13, 70.20, 70.23, 70.25, 70.29, 70.40, 71.06, 72.05, 72.10, 72.30, 73.55, 77.51, 83.13, 97.12, 99.76, 106.70, 119.22, 124.72, 129.69, 140.34, 144.24, 152.23, 166.07. (MALDI-TOF) m/z: calculated for $C_{106}H_{189}N_{13}O_{51}$ [M-H]$^{+}$: 2459.2517 found 2459.3218.

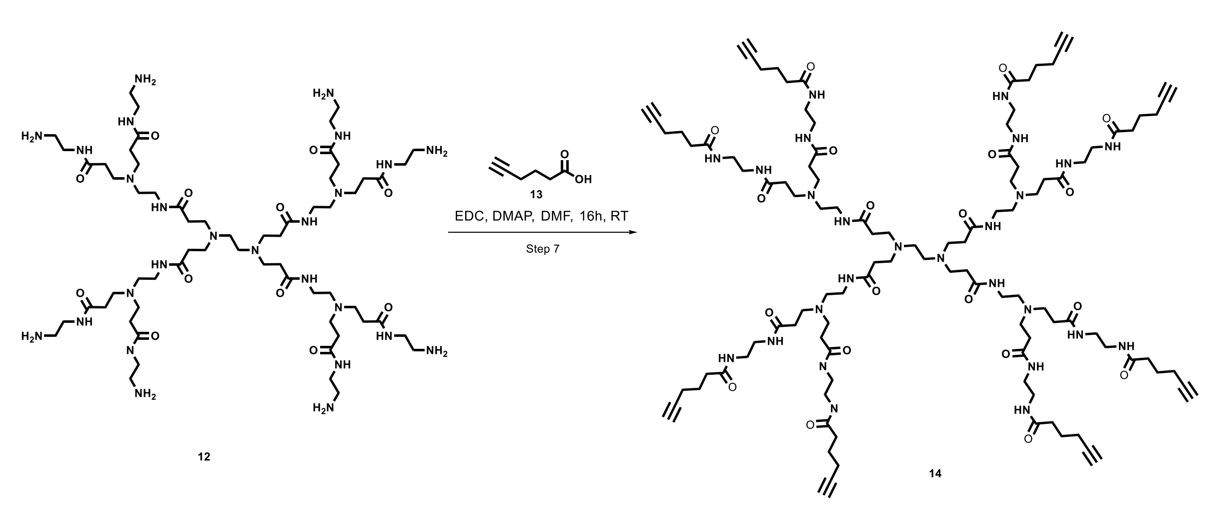

Synthesis of compound 14: Hexynoic Acid **13** (2g, 24 eq, 18.48 mmol) was dissolved in Anhy. DMF (5 mL) and was activated by adding EDC (3.5g, 24 eq, 18.48 mmol) and stirred for 15 minutes. It was then added dropwise to the G1 PAMAM dendrimer (1.1g, 1.0 eq, 0.77 mmol) solution under continuous stirring followed by addition of DMAP (1.1g, 12 eq, 9.24 mmol) and the reaction mixture was stirred for 24 hours at room temperature. Upon completion, the dialysis was performed using a 1 kDa dialysis membrane in DMF for 12h. The final dialysis was performed against DI water. The aqueous solution was lyophilized to afford compound **14** in 84% yield.

^{1}H NMR (500 MHz, DMSO-d_6) δ 1.62 – 1.70 (m, 16H), 2.01 – 2.34 (m, 63H), 2.37 – 2.48 (m, 13H), 2.54 – 3.02 (m, 39H), 3.17 (s, 14H), 3.33 (s, 11H), 7.61 – 8.21 (m, 20H). ^{13}C NMR (125 MHz, DMSO-d_6) δ 17.87, 24.65, 33.71, 34.64, 37.36, 38.78, 38.82, 50.01, 50.14, 52.61, 55.37, 71.90, 84.56, 171.73, 172.02, 172.13.

Synthesis of compound 15: To a stirred solution of compound **14** (52 mg, 1.0 eq, 0.024 mmol) in DMF (4 mL) in a microwave vial was added solution of compound **10** (613 mg, 9.0 eq, 0.191 mmol) dissolved in DMF (4 mL). To this solution, $CuSO_4.5H_2O$ (6.0 mg, 1.0 eq, 0.024 mmol) and sodium ascorbate (7.0 mg, 1.5 eq, 0.037 mmol) dissolved in minimum amount of deionized water were added. The reaction was performed in microwave condition at 40 ^{o}C for 12 h. The completion of reaction was tracked using HPLC. Upon completion, the dialysis was performed using a 3.5 kDa dialysis membrane in DMF for 12h. The final dialysis was performed in DI water. The product was lyophilized to afford compound **15** in 90% yield.

^{1}H NMR (500 MHz, DMSO-d_6) δ 1.72 – 1.78 (m, 24H), 1.93 – 2.01 (m, 216H), 2.10 (dd, J = 13.0, 5.5 Hz, 24H), 3.34 – 3.62 (m, 1024H), 3.63 – 3.70 (m, 44H), 3.70 – 3.88 (m, 108H), 3.87

– 4.10 (m, 68H), 4.09 – 4.22 (m, 60H), 4.40 – 4.61 (m, 116H), 4.84 (t, *J* = 9.8 Hz, 26H), 4.99 (d, *J* = 3.3 Hz, 22H), 5.06 – 5.19 (m, 24H), 7.18 (s, 16H), 7.82 (s, 7H, NH), 8.04 (s, 24H, triazole-H), 8.42 – 8.50 (m, 8H, triazole-H). ^{13}C NMR (125 MHz, DMSO-d_6) δ 20.89, 20.98, 21.11, 34.93, 49.75, 62.49, 63.97, 66.57, 67.70, 68.81, 68.96, 69.16, 69.36, 69.40, 69.82, 69.98, 70.07, 70.13, 70.21, 70.24, 70.26, 70.30, 70.30, 70.41, 72.32, 96.60, 106.71, 124.74, 152.24, 169.96, 170.22, 170.57.

Synthesis of compound 16 (*2DG-D*): To a stirred solution of compound **14** (483 mg, 1.0 eq, 0.222 mmol) in DMF (5 mL) in a microwave vial was added solution of compound **11** (5 mg, 9.0 eq, 2 mmol) dissolved in deionized water (5 mL). To this solution, $CuSO_4.5H_2O$ (55.0 mg, 1.0 eq, 0.222 mmol) and sodium ascorbate (66.6 mg, 1.5 eq, 0.333 mmol) dissolved in minimum amount of deionized water was added. The reaction vial was irradiated under microwave conditions at 80 ^{o}C for 15h. The completion of reaction was tracked using HPLC. Upon completion, the dialysis was performed using a 3.5 kDa dialysis membrane in DMF for 12h. The final dialysis was performed in D.I. water. The product was lyophilized to afford compound **16** in 95% yield.

^{1}H NMR (500 MHz, DMSO-d_6) δ 1.44 (dd, *J* = 12.3, 3.6 Hz, 24H), 1.87 (dd, *J* = 12.9, 5.2 Hz, 24H), 2.97 – 3.13 (m, 60H), 3.47 – 3.70 (m, 1175H), 3.71 – 3.90 (m, 155H), 4.04 – 4.20 (m, 53H), 4.38 – 4.56 (m, 132H), 4.70 – 4.93 (m, 65H), 7.18 (s, 16H), 7.78 – 7.91 (m, 12H), 8.04 (s, 24H), 8.47 (t, *J* = 5.7 Hz, 8H). ^{13}C NMR (125 MHz, D_2O) δ 36.58, 37.19, 39.42, 39.55, 49.94, 60.29, 60.59, 60.88, 63.02, 65.99, 67.90, 68.12, 68.34, 68.66, 68.83, 68.91, 69.10, 69.39, 69.41, 69.49, 69.53, 69.55, 69.58, 69.61, 69.66, 69.83, 70.12, 70.40, 70.77, 70.92, 71.08, 71.67, 71.94, 72.19, 75.95, 91.23, 93.33, 97.32, 106.28, 125.42, 139.68, 151.88.

Synthesis of compound 17: Hexynoic Acid **13** (3.16 mg, 4.0 eq, 0.028 mmol) in Anhy. DMF (5 mL) was taken, EDC (6.75 mg, 0.035 mmol) was added, and stirred for 15 minutes. To stirred solution of 2DG dendrimer **16** (154 mg, 1.0 eq, 0.007 mmol) in DMF (5 mL) was added hexynoic acid solution dropwise under continuous stirring followed by the addition of DMAP (4.3mg, 0.035mmol) and the reaction mixture was stirred for 24 hours at room temperature. The reaction completion was confirmed by the shift in the retention time of the chromatogram in HPLC. The dialysis was performed using a 1 kDa dialysis membrane in DMF for 12h. The final dialysis was performed against D.I. water. The product was lyophilized to afford compound **17** in 84% yield.

^{1}H NMR (500 MHz, DMSO) δ 1.43 (dd, *J* = 14.3, 10.8 Hz, 24H), 1.65 – 1.75 (m, 25H), 1.79 (dd, *J* = 10.4, 5.4 Hz, 25H), 1.84 – 1.92 (m, 24H), 2.09 – 2.16 (m, 25H), 2.16 – 2.25 (m, 43H),

2.36 – 2.44 (m, 32H), 2.58 (t, *J* = 7.8 Hz, 29H), 2.61 – 2.83 (m, 71H), 2.89 (s, 9H), 2.98 – 3.15 (m, 95H), 3.26 – 3.48 (m, 535H), 3.55 – 3.69 (m, 252H), 3.78 (dt, *J* = 20.2, 5.2 Hz, 160H), 4.03 – 4.18 (m, 88H), 4.30 (d, *J* = 11.5 Hz, 12H), 4.40 – 4.56 (m, 178H), 4.73 (s, 13H), 4.80 – 4.92 (m, 45H), 5.08 – 5.17 (m, 8H), 7.18 (s, 16H), 7.79 – 7.87 (m, 20H), 7.95 (s, 8H), 8.04 (s, 24H), 8.46 (t, *J* = 5.8 Hz, 8H). ^{13}C NMR (125 MHz, DMSO-d$_6$) δ 17.53, 23.97, 25.10, 25.61, 35.31, 38.25, 38.33, 38.79, 49.63, 49.76, 60.67, 61.48, 63.96, 64.15, 66.05, 66.24, 68.42, 68.82, 69.17, 69.23, 69.41, 69.49, 69.99, 70.01, 70.07, 70.14, 70.21, 70.23, 70.25, 70.30, 70.41, 70.49, 72.12, 72.18, 72.33, 72.80, 73.56, 97.14, 97.23, 106.72, 122.71, 124.72, 140.36, 144.25, 152.24.

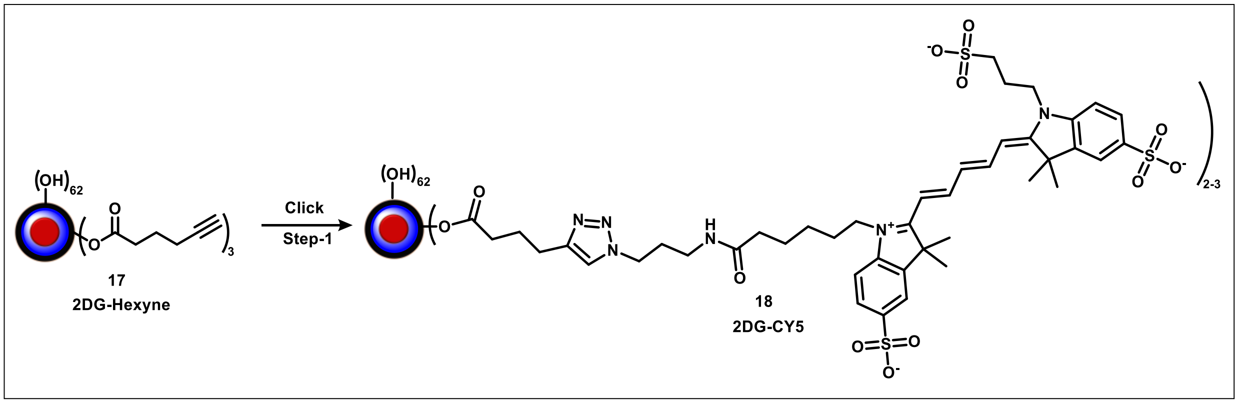

Synthesis of 2DG-D-Cy5 compound 18: To a stirred solution of compound **17** (27mg, 1.0 eq, 0.00120 mmole) in 1 mL DI water in a microwave vial was added solution of Cy5 Azide (4.3 mg, 3.5 eq, 0.00417 mmole) dissolved in 1 mL DMF followed by the addition of $CuSO_4.5H_2O$ (10 mol% per acetylene) dissolved in 0.1mL DI water. After 2 minutes, Sodium Ascorbate (15 mol% per acetylene) was added, and the reaction vial was irradiated under microwave at 40°C for 10h. Upon completion, the dialysis was performed using a 1 kDa dialysis membrane against pure water for 12h. The aqueous solution was lyophilized to afford compound **18** in 87% yield.

^{1}H NMR (500 MHz, DMSO-d$_6$) δ 1.25 (dd, *J* = 11.8, 5.3 Hz, 25H), 1.38 – 1.57 (m, 28H), 1.68 (s, 34H), 1.87 (dd, *J* = 13.4, 5.5 Hz, 31H), 1.99 – 2.14 (m, 21H), 2.19 (t, *J* = 7.1 Hz, 12H), 2.80 (s, 8H), 2.92 – 3.10 (m, 38H), 3.13 – 3.68 (m, 982H), 3.71 – 3.89 (m, 69H), 3.99 – 4.19 (m, 51H), 4.22 – 4.33 (m, 14H), 4.39 – 4.60 (m, 80H), 4.73 (d, *J* = 4.9 Hz, 9H), 4.78 – 4.90 (m, 24H), 5.14 (d, *J* = 5.5 Hz, 6H), 6.30 (d, *J* = 13.4 Hz, 6H), 7.18 (s, 16H), 7.32 (d, *J* = 7.7 Hz, 7H), 7.54 – 8.21 (m, 58H), 8.30 – 8.57 (m, 18H).

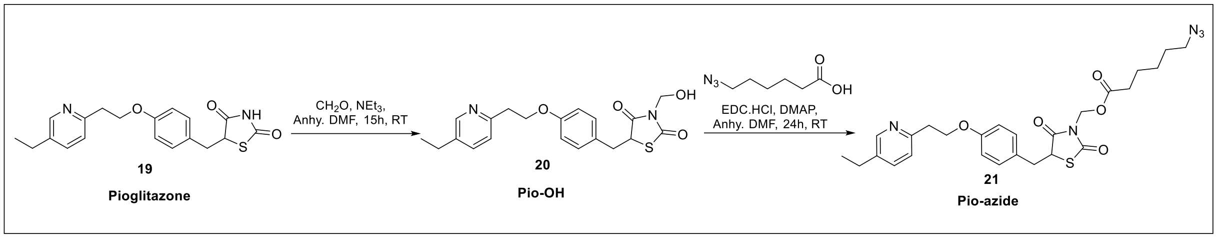

Synthesis 5-(4-(2-(5-ethylpyridin-2-yl)ethoxy)benzyl)-3-(hydroxymethyl)thiazolidine-2,4-dione (20): To a stirred solution of Pioglitazone (1 g, 1.0 eq, 2.8 mmol) in a 6 mL was added formaldehyde (33.6 mg, 4.5 eq, 11.2 mmol) and triethylamine (1.135 g, 4.5 eq, 11.2 mmol) under inert atmosphere. The reaction mixture was stirred overnight at room temperature. After completion of the reaction, DCM was added to reaction mixture and filtered. Filtrate was washed with water (3 × 50 mL) and chilled brine (2 × 50 ml), dried over Na_2SO_4, and evaporated in vacuo. The crude product was purified by silica flash column chromatography [methanol/dichloromethane, 9:91 (v/v)] to afford compound **20** in 90% yield.

^{1}H NMR (500 MHz, DMSO-d$_6$) δ 1.18 (t, *J* = 7.5 Hz, 3H), 2.59 (q, *J* = 7.6 Hz, 2H), 3.05 – 3.18 (m, 3H), 3.55 – 3.74 (m, 1H), 3.85 – 4.03 (m, 1H), 4.29 (q, *J* = 6.4 Hz, 2H), 4.73 (dd, *J* = 7.6, 1.6 Hz, 1H), 5.66 (h, *J* = 5.4 Hz, 1H), 6.54 (t, *J* = 7.5 Hz, 1H), 6.77 – 6.88 (m, 2H), 7.07 (t, *J* = 9.1 Hz, 2H), 7.27 (dd, *J* = 8.0, 2.4 Hz, 1H), 7.57 (dd, *J* = 7.9, 2.4 Hz, 1H), 8.36 (d, *J* = 2.3 Hz, 1H). ^{13}C NMR (125 MHz, DMSO-d$_6$) δ 15.88, 24.39, 25.43, 25.97, 28.36, 33.70, 37.23, 38.63, 38.82, 50.90, 66.29, 66.40, 67.12, 70.10, 79.17, 79.43, 79.69, 114.45, 114.54, 123.52, 126.57, 127.40, 131.93, 132.06, 136.18, 137.15, 149.02, 155.89, 158.00, 158.19, 171.89, 172.43, 177.91.

Synthesis 5-(4-(2-(5-ethylpyridin-2-yl)ethoxy)benzyl)-2,4-dioxothiazolidin-3-yl 6-azidohexanoate (21): The solution of 6-azidohexanoic acid (814.14 mg, 2.0eq, 5.174 mmol) in Anhy. DMF (5 mL) was activated by adding EDC (992 mg, 2.0eq, 5.174 mmol) and stirred for 15 minutes. To this stirring solution, compound **19 (**1 g, 1.0 eq, 2.587 mmol) was added followed by addition of DMAP (231 mg, 0.8 eq, 2.32 mmol) and the reaction mixture was stirred for 24 hours at room temperature. Upon completion, DCM was added to reaction mixture and filtered. Filtrate was washed with water (3 × 50 mL) and chilled brine (2 × 50 ml), dried over Na_2SO_4, and evaporated in vacuo. The crude product was purified by silica flash column chromatography [methanol/dichloromethane, 10:90 (v/v)] to afford Pio-azide compound **20** in 70% yield.

^{1}H NMR (500 MHz, DMSO-d_6) δ 1.18 (t, *J* = 7.6 Hz, 3H), 1.30 (tt, *J* = 9.9, 6.3 Hz, 2H), 1.45 – 1.56 (m, 4H), 2.32 (t, *J* = 7.3 Hz, 2H), 2.59 (q, *J* = 7.6 Hz, 2H), 3.12 (q, *J* = 6.1 Hz, 3H), 3.21 (d, *J* = 13.9 Hz, 1H), 3.29 (t, *J* = 6.9 Hz, 3H), 4.30 (t, *J* = 6.7 Hz, 2H), 4.38 (d, *J* = 11.4 Hz, 1H), 4.53 (d, *J* = 11.5 Hz, 1H), 6.80 – 6.91 (m, 2H), 7.07 – 7.14 (m, 2H), 7.27 (d, *J* = 7.9 Hz, 1H), 7.57 (dd, *J* = 7.9, 2.3 Hz, 1H), 8.37 (d, *J* = 2.3 Hz, 1H). ^{13}C NMR (125 MHz, DMSO-d_6) δ 15.88, 24.39, 25.43, 25.97, 28.36, 33.69, 37.22, 38.79, 50.89, 66.36, 66.42, 67.13, 79.17, 79.43, 79.70, 114.56, 123.53, 126.54, 132.07, 136.19, 137.16, 149.04, 155.88, 158.21, 172.43. (ESI) *m/z*: calculated for $C_{26}H_{31}N_3O_5S$ $[M+H]^+$:526.20 found 526.14.

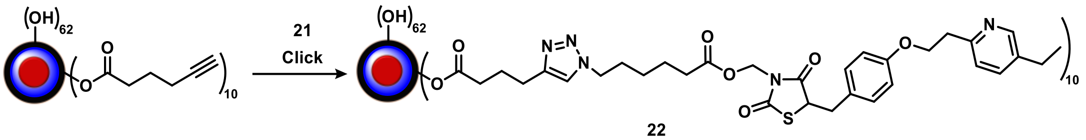

2DG-Hexyne

22
2DG-PIO

Synthesis of compound 22 (*2DG-D-Pio*): To a stirred solution of 2DG-D-$Hexyne_{10}$ (100 mg, 1.0 eq, 0.0043 mmol) in 1 mL DI Water in a microwave vial was added solution of compound **21** (22.68 mg, 10 eq, 0.043 mmol) dissolved in 1 mL DMF followed by the addition of $CuSO_4.5H_2O$ (10 mol% per acetylene) dissolved in 1mL DI water. After 2 minutes, Sodium Ascorbate (15 mol% per acetylene) was added, and the reaction vial was irradiated under microwave at 40°C for 10h. Upon completion, the dialysis was performed using a 1 kDa dialysis membrane against D.I. water for 12h. The aqueous solution was lyophilized to afford final compound **22** in 95% yield.

^{1}H NMR (500 MHz, DMSO-d_6) δ 1.07 – 1.34 (m, 73H), 1.34 – 1.56 (m, 32H), 1.63 – 1.93 (m, 59H), 2.10 (d, *J* = 7.4 Hz, 16H), 2.15 – 2.26 (m, 39H), 2.34 (d, *J* = 7.5 Hz, 20H), 2.53 – 2.67 (m, 87H), 2.70 (s, 51H), 2.96 – 3.14 (m, 118H), 3.25 (s, 141H), 3.29 – 3.69 (m, 3741H), 3.70 – 3.86 (m, 182H), 4.01 – 4.17 (m, 87H), 4.21 – 4.35 (m, 52H), 4.40 – 4.56 (m, 155H), 4.81 (d, *J* = 3.7 Hz, 18H), 6.79 (d, *J* = 8.2 Hz, 20H), 7.07 (d, *J* = 8.2 Hz, 20H), 7.17 (s, 20H), 7.26 (d, *J* = 7.9 Hz, 10H), 7.55 (d, *J* = 8.0 Hz, 8H), 7.78 – 7.90 (m, 31H), 7.95 (s, 6H), 8.03 (s, 32H), 8.35 (s, 8H), 8.47 (s, 7H). ^{13}C NMR (125 MHz, DMSO-d_6) δ 15.86, 24.27, 24.77, 25.10, 25.42, 25.61, 25.72, 33.74, 37.26, 38.32, 38.79, 49.44, 49.62, 49.75, 50.92, 59.26, 61.47, 63.95, 66.05, 67.07, 68.42, 68.80, 69.16, 69.23, 69.40, 69.48, 69.98, 70.00, 70.06, 70.13, 70.20, 70.23, 70.25, 70.29,

70.40, 72.11, 72.32, 73.55, 97.13, 106.70, 114.27, 123.48, 124.71, 131.87, 136.17, 144.24, 149.01, 152.22, 173.85.

1.4 In vitro drug release and stability studies.

Drug release studies were conducted *in vitro* under both plasma conditions (PBS pH 7.4 and 2% FBS in PBS) and intracellular conditions (citrate buffer pH 5.5 with esterase). *2DG-D-Pio* was dissolved at a concentration of 1 mg/mL in each buffer and subjected to incubation at 37 °C with continuous shaking to mimic physiological conditions. At designated time intervals, samples were withdrawn, promptly quenched with an equal volume of methanol, and stored at −20 °C until further use. The released drug was subsequently analyzed using HPLC, and the extent of drug release was determined by comparison to the standard curve established for free *Pio* on HPLC.

1.5 2DG-D-Pio formulation stability studies.

2DG-D-Pio was formulated at 50mg/mL in PBS and sterile filtered through 0.4µm filters. The formulations were kept at either room temperature or 4°C. The aliquots were analyzed via HPLC for the purity at 0h, 1-d, 7-d, and 28-d time points.

2. Supplementary Figures.

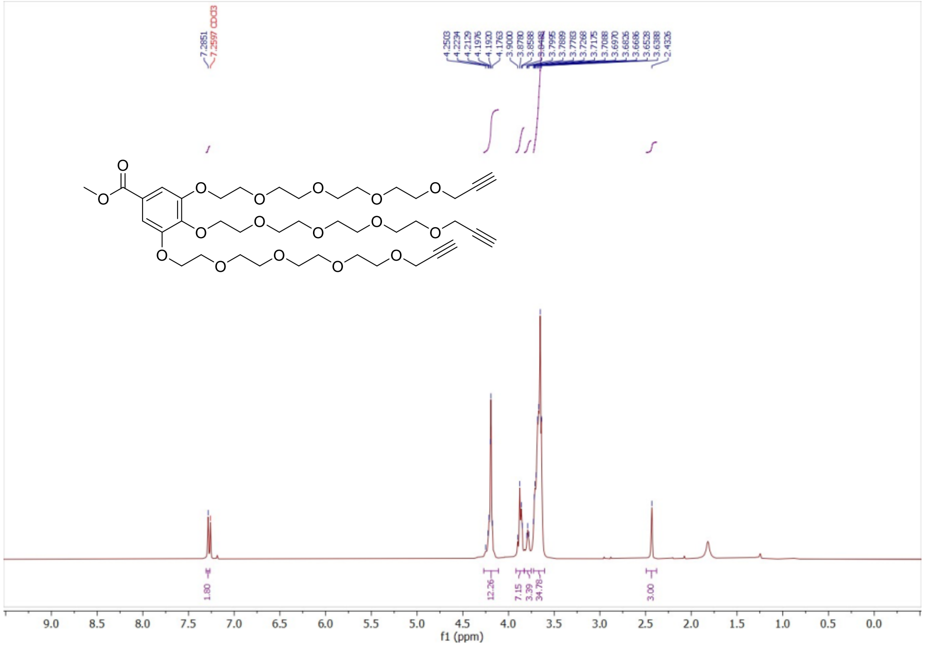

Figure S1: ^{1}H NMR of compound **3** in $CDCl_3$ (500 MHz).

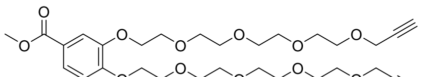

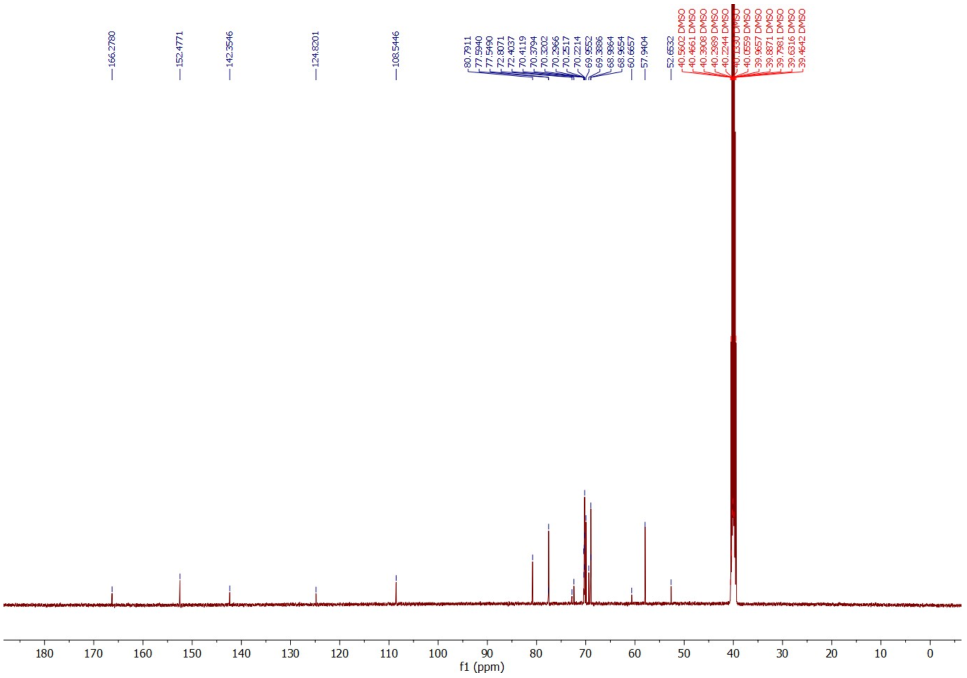

Figure S2: ^{13}C NMR of compound **3** in $CDCl_3$ (125 MHz).

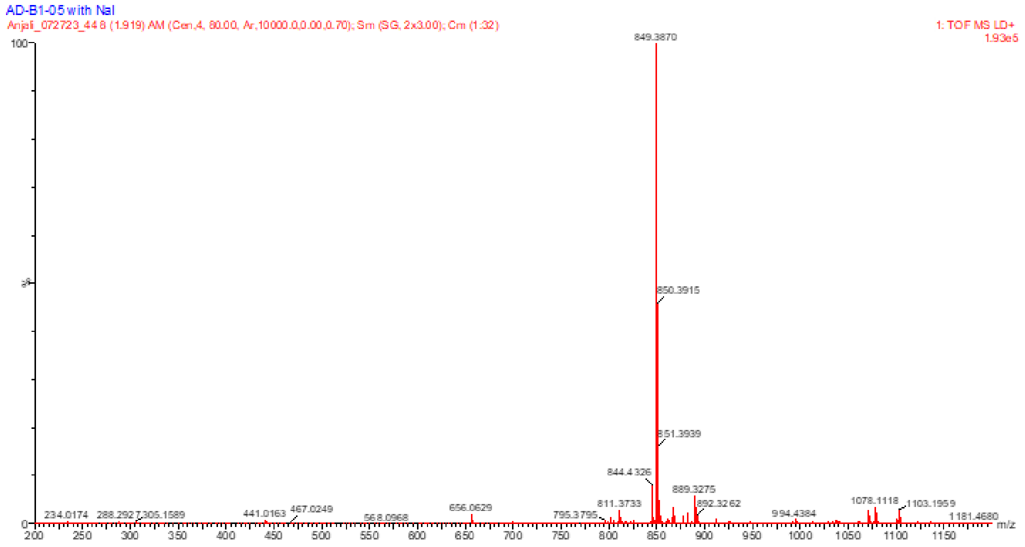

.

Figure S3: Mass spectrum (MALDI-ToF) of compound **3**

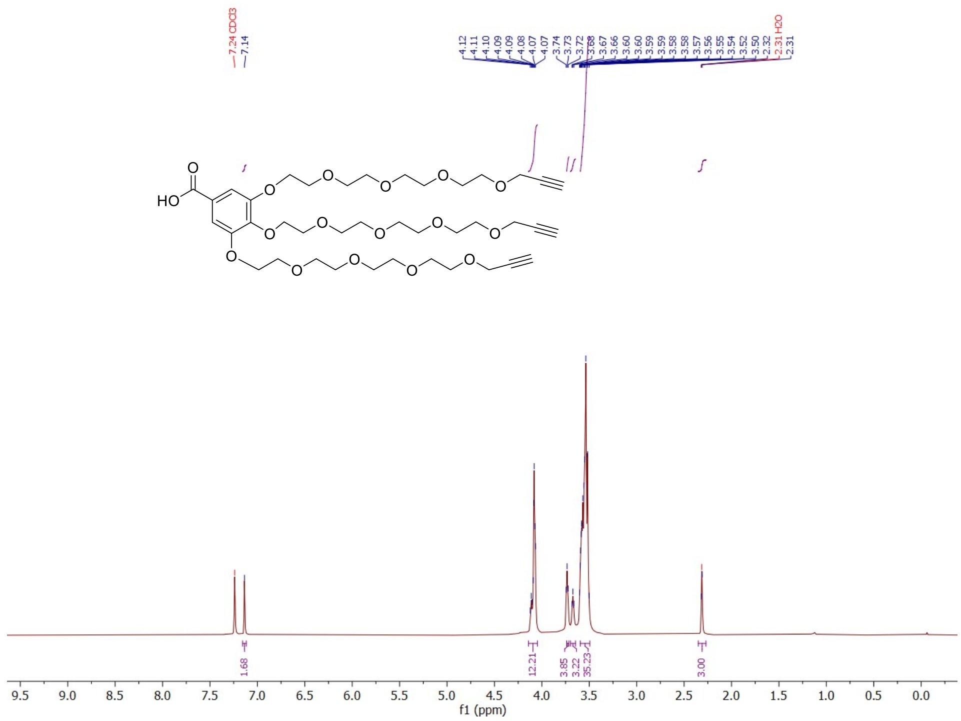

Figure S4: ^{1}H NMR of compound **4** in $CDCl_3$ (500 MHz).

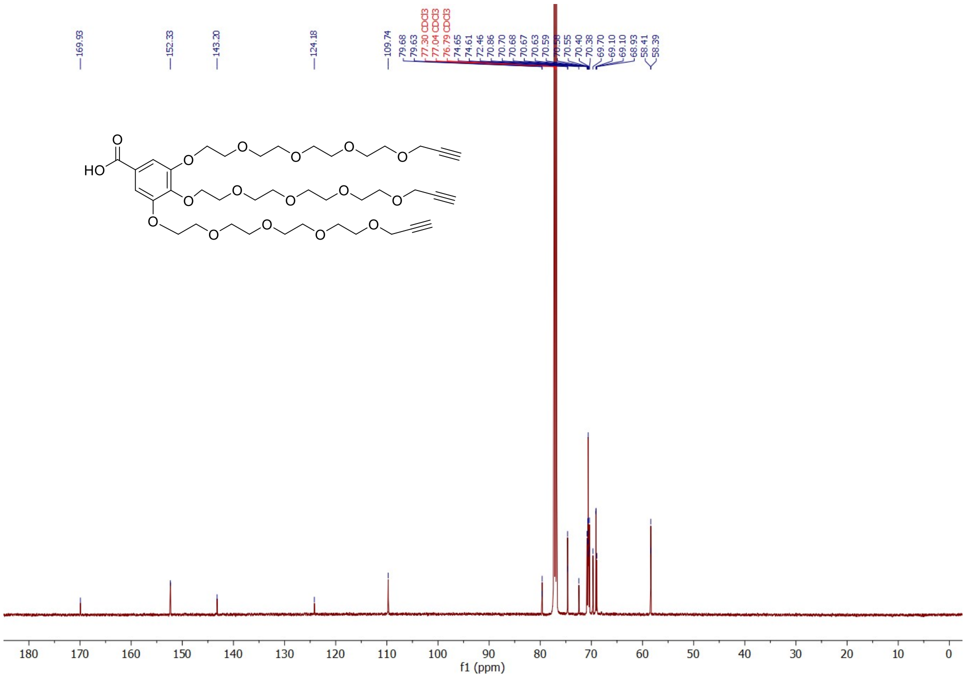

Figure S5: ^{13}C NMR of compound **4** in $CDCl_3$ (125 MHz).

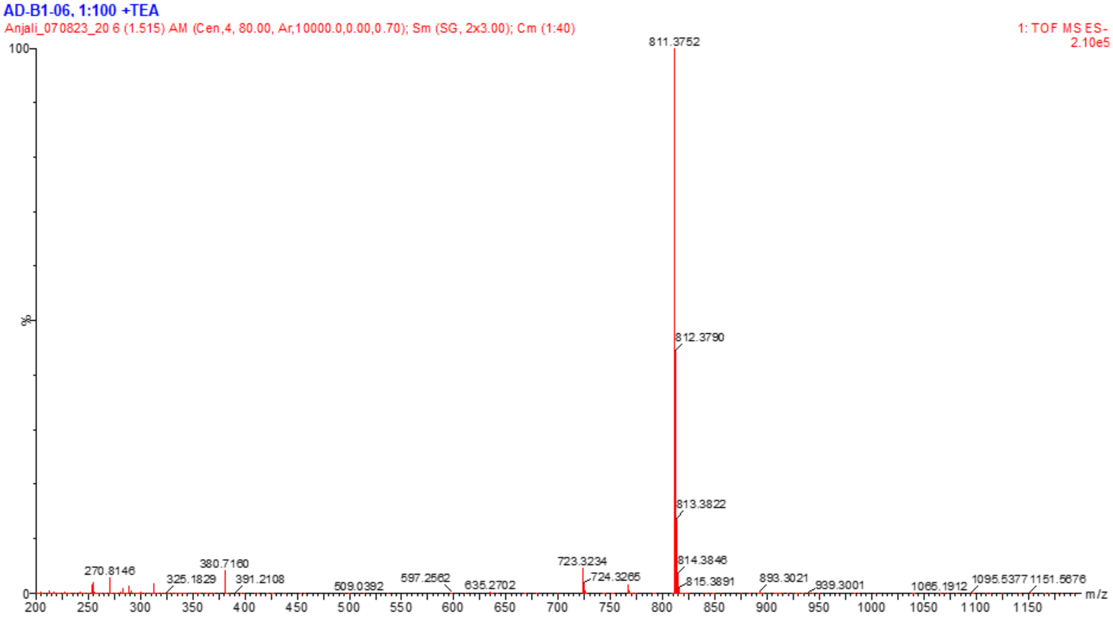

Figure S6: Mass spectrum (TOF MS ES-) of compound **4**.

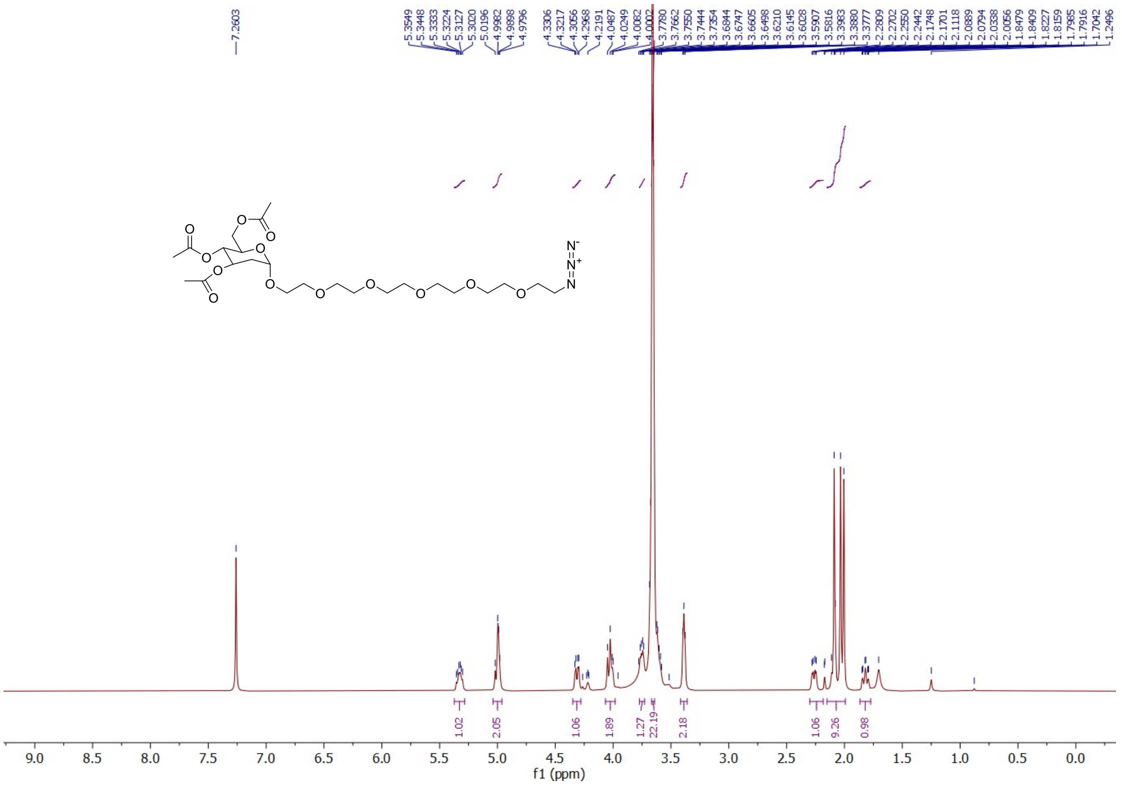

Figure S7: ^{1}H NMR of compound **7** in $CDCl_3$ (500 MHz).

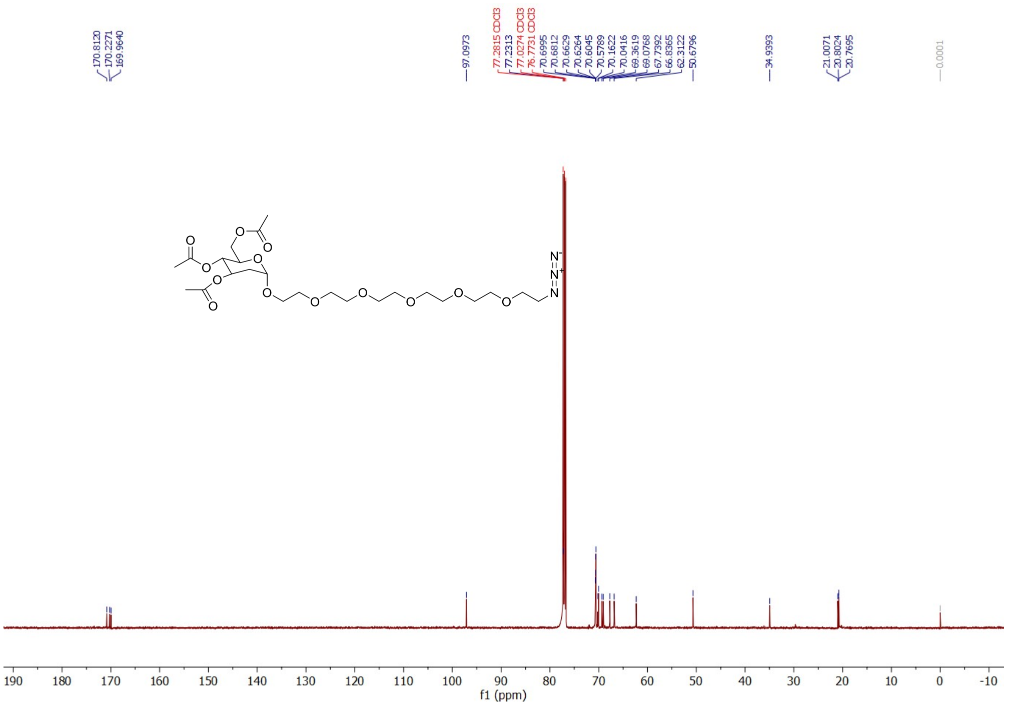

Figure S8: ^{13}C NMR of compound **7** in $CDCl_3$ (125 MHz).

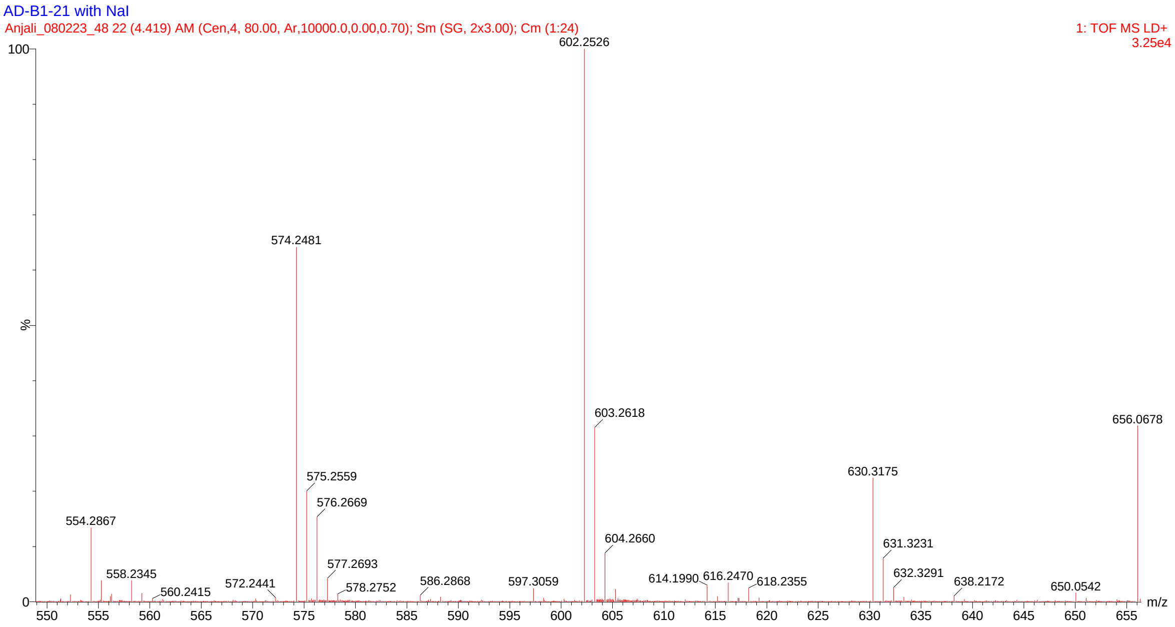

Figure S9: Mass spectrum (MALDI ToF) of compound **7**.

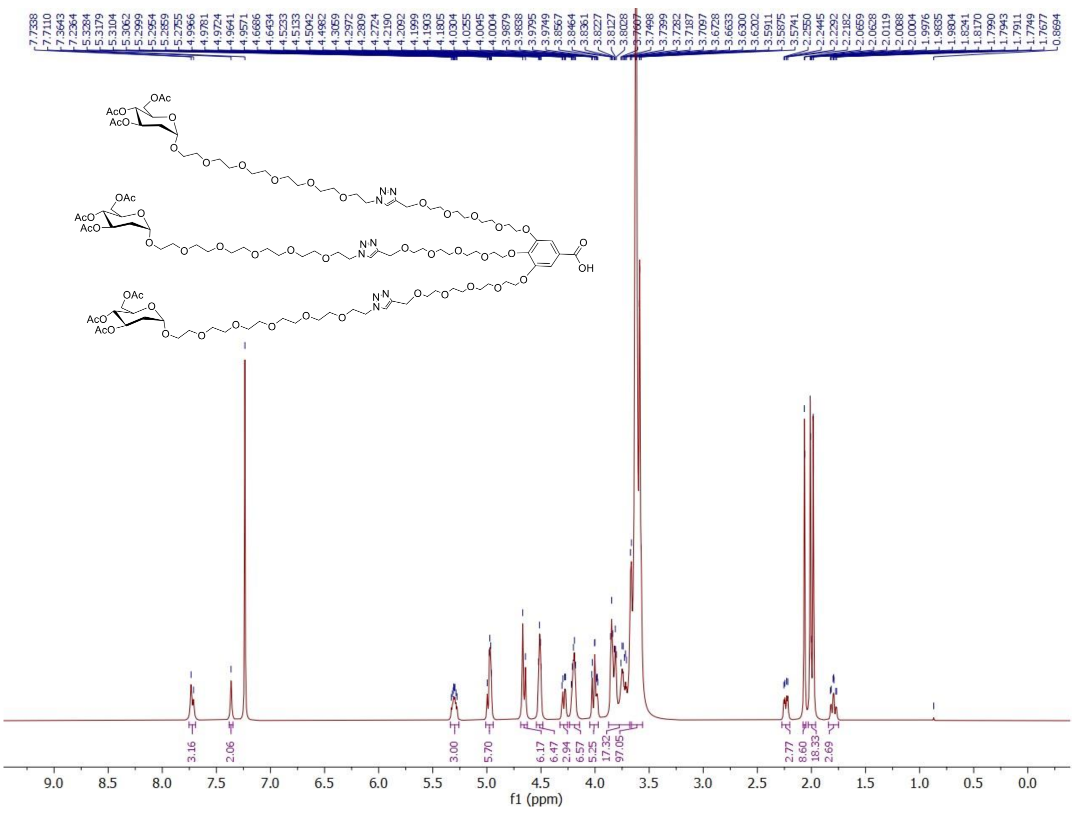

Figure S10: A. ^{1}H NMR of compound **8** in $CDCl_3$ (500 MHz).

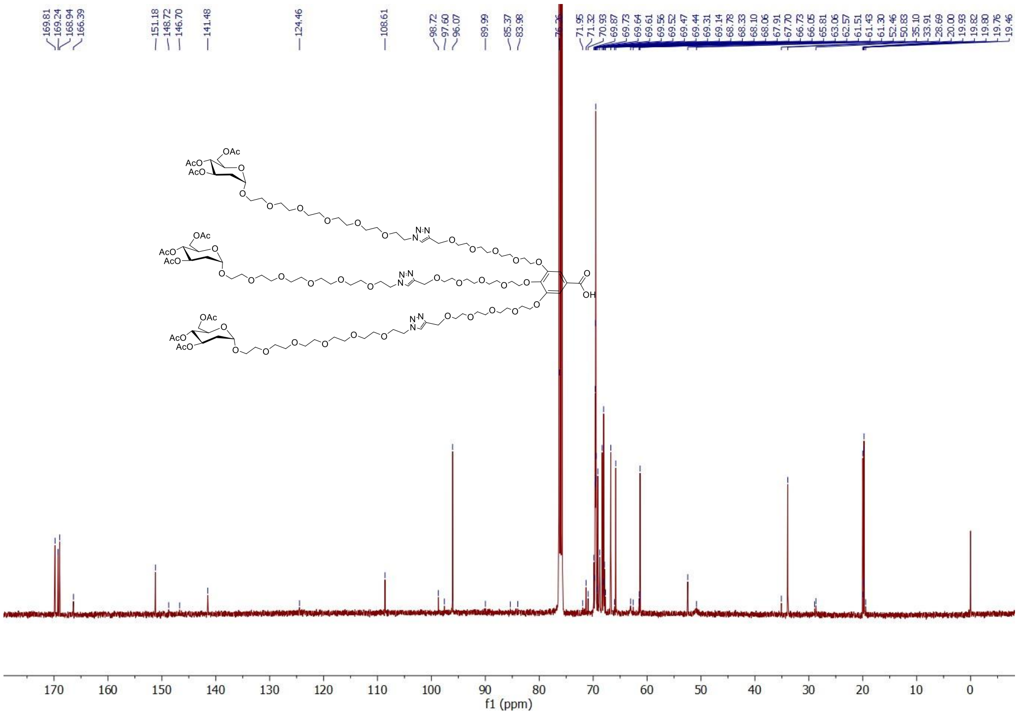

Figure S11: ^{13}C NMR of compound **8** in $CDCl_3$ (125 MHz).

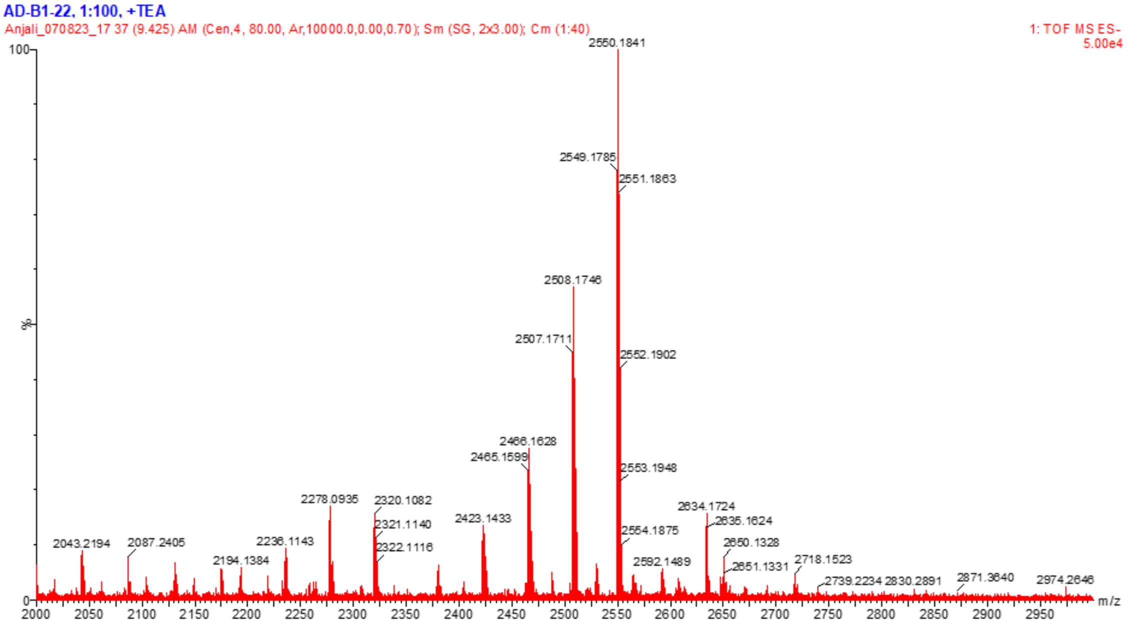

Figure S12: Mass spectrum (TOF MS ES-) of compound **8.**

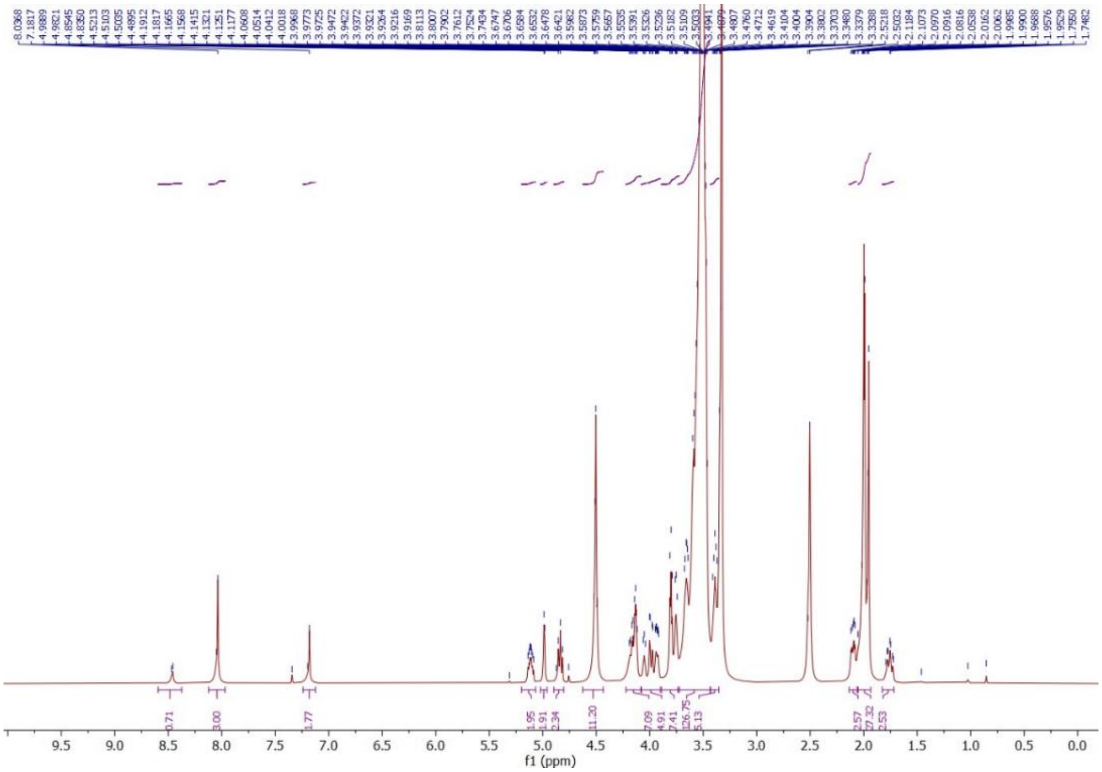

Figure S13: ^{1}H NMR of compound **10** in DMSO-d_6 (500 MHz).

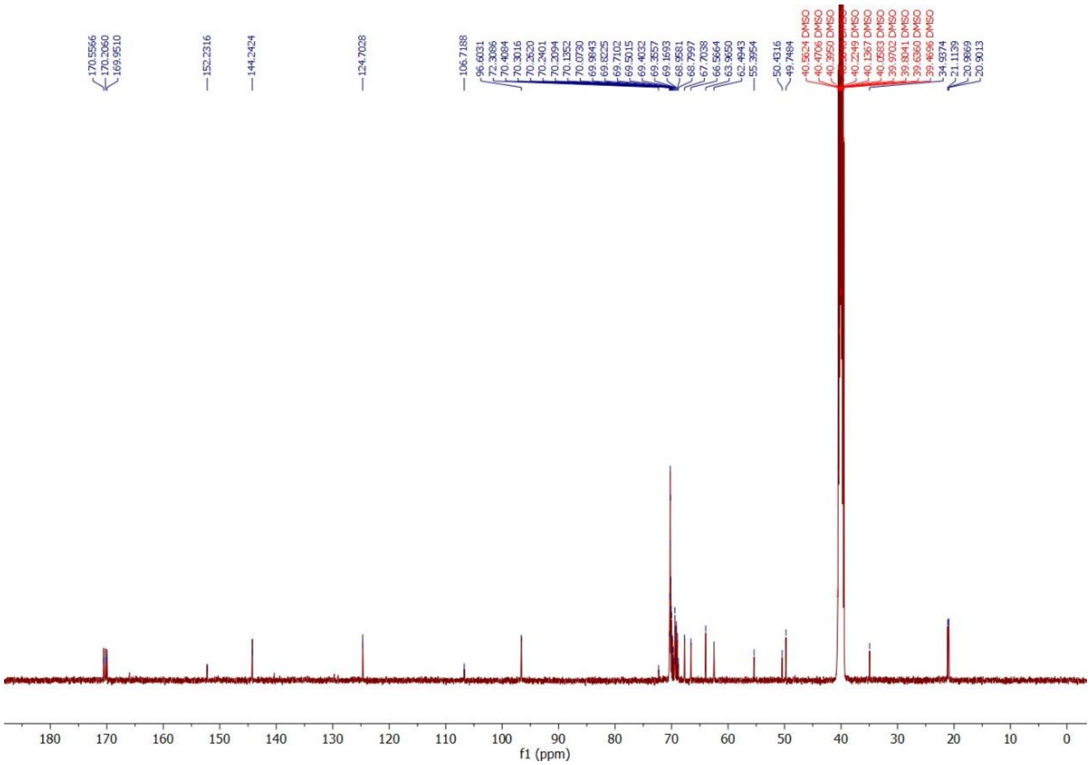

Figure S14: ^{13}C NMR of compound **10** in DMSO-d$_6$ (125 MHz).

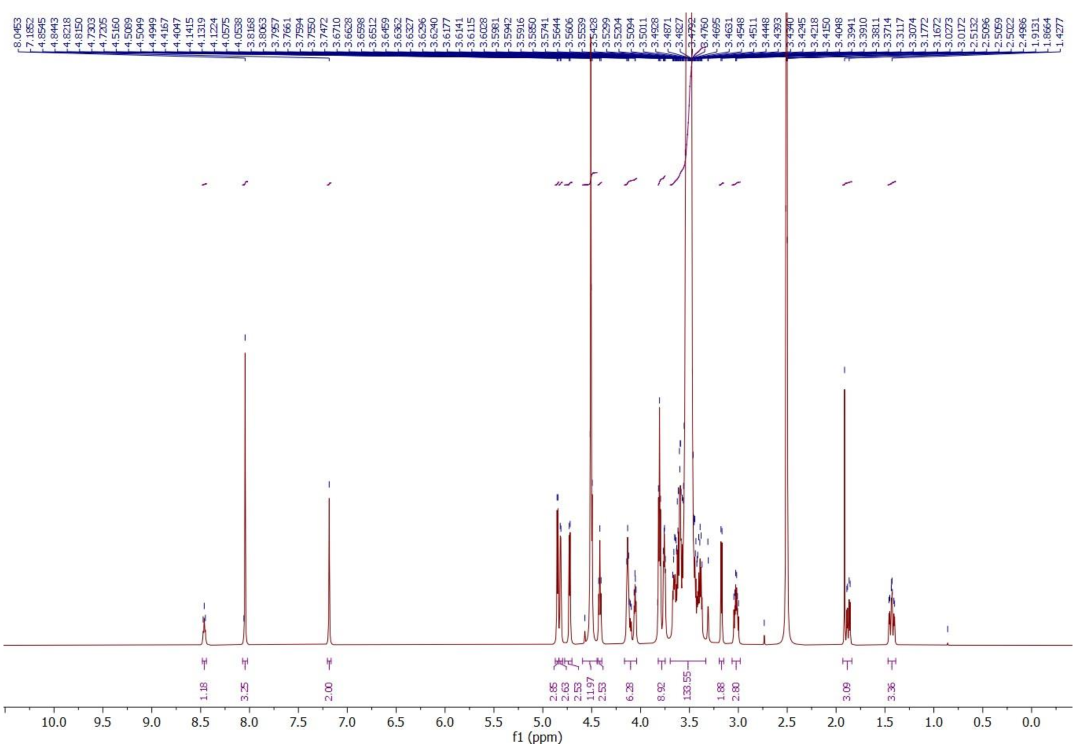

Figure S15: ^{1}H NMR of compound **11** in DMSO-d_6 (500 MHz).

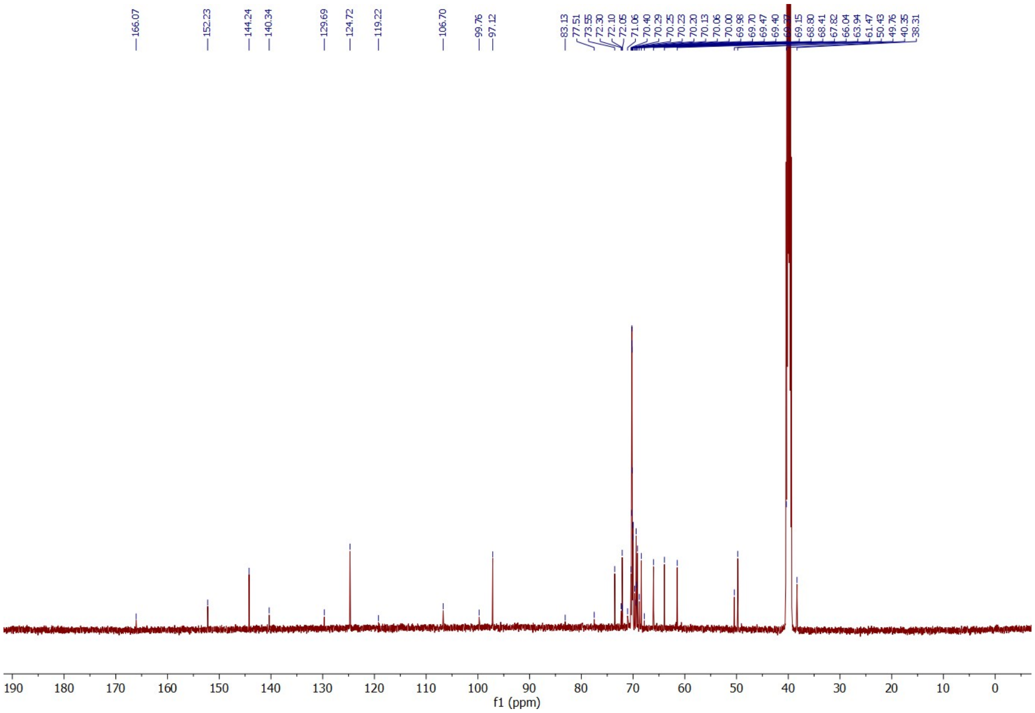

Figure S16: ^{13}C NMR of compound **11** in DMSO-d_6 (125 MHz).

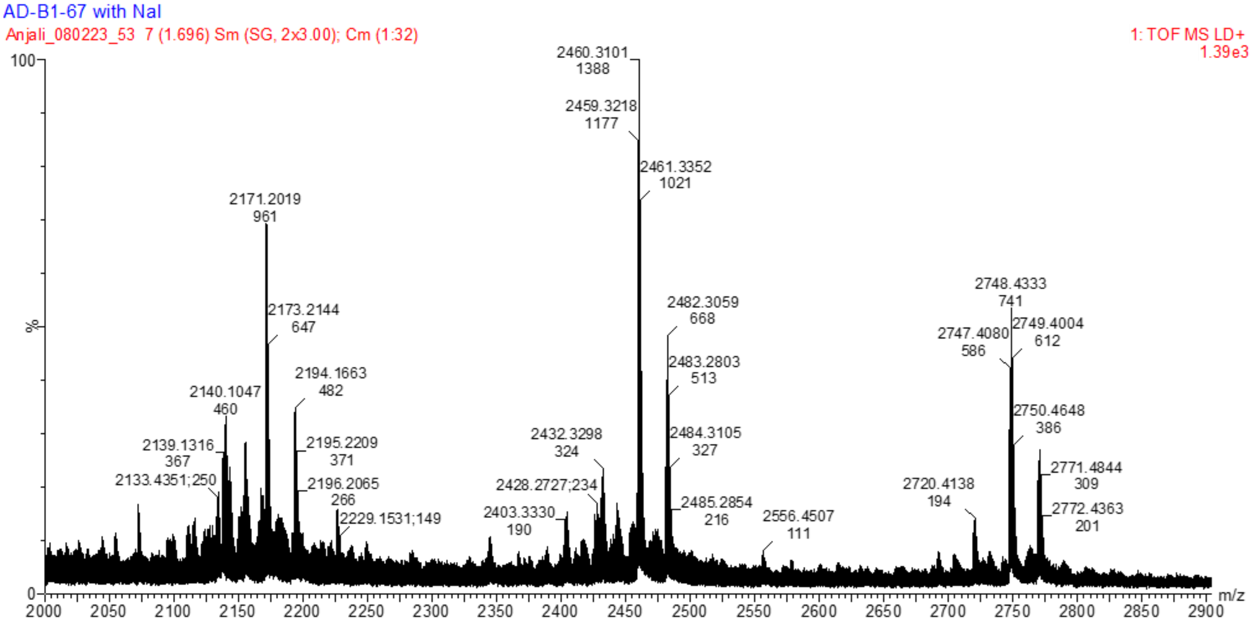

Figure S17: Mass spectrum (MALDI-ToF) of compound **11**

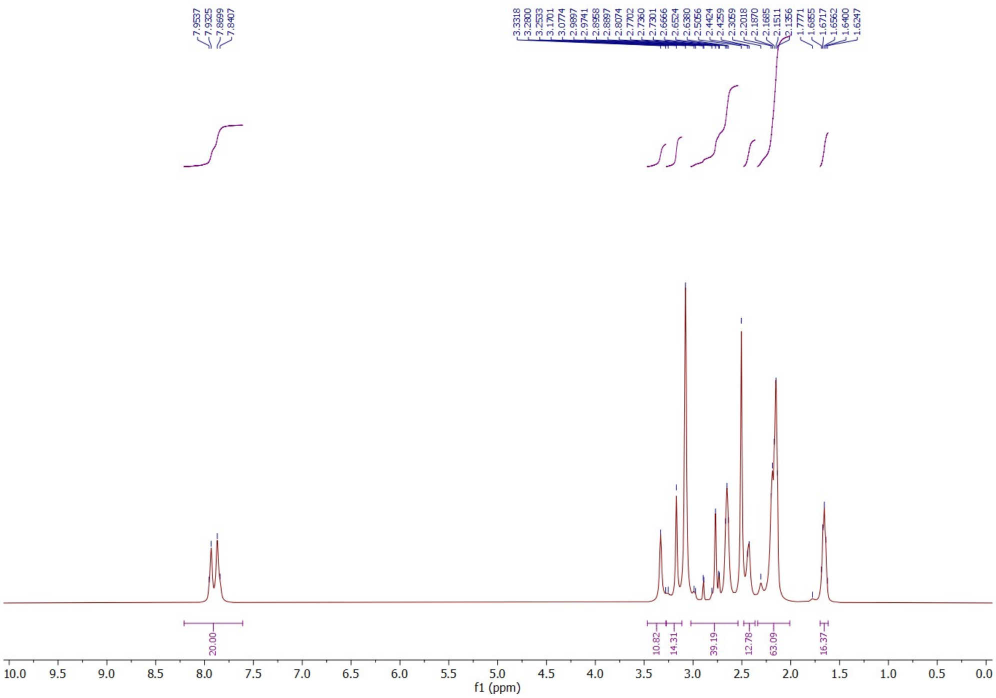

Figure S18: ^{1}H NMR of compound **14** in DMSO-d_6 (500 MHz).

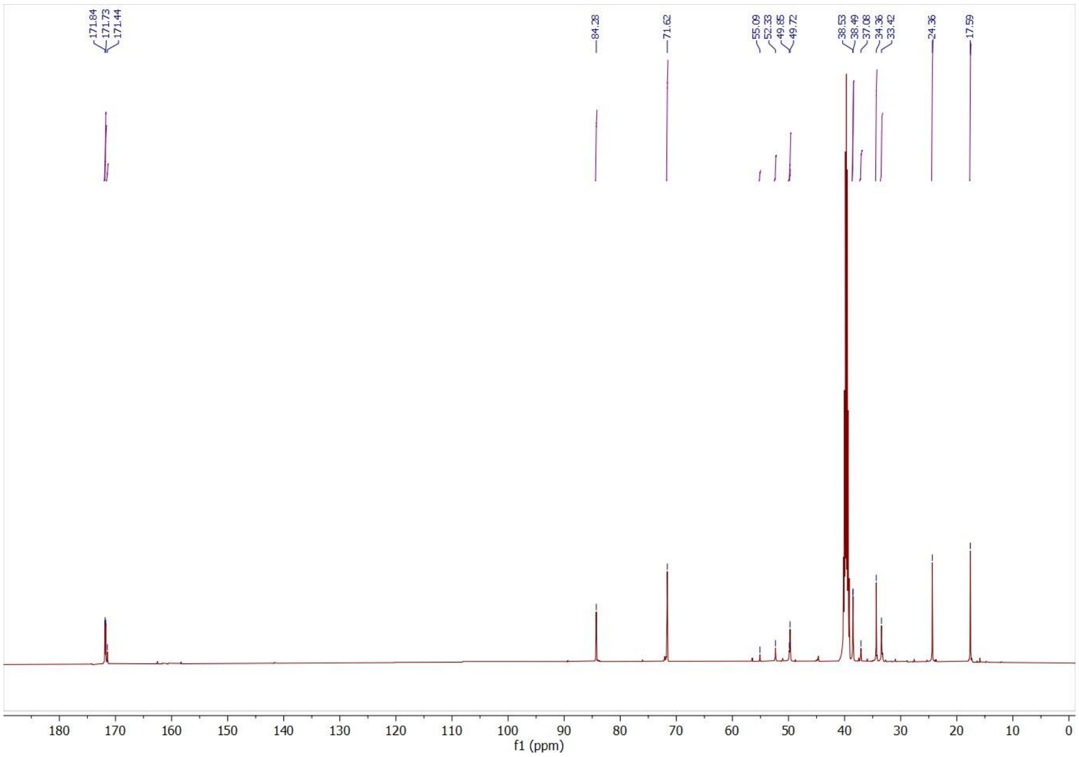

Figure S19: ^{13}C NMR of compound **14** in DMSO-d_6 (125 MHz).

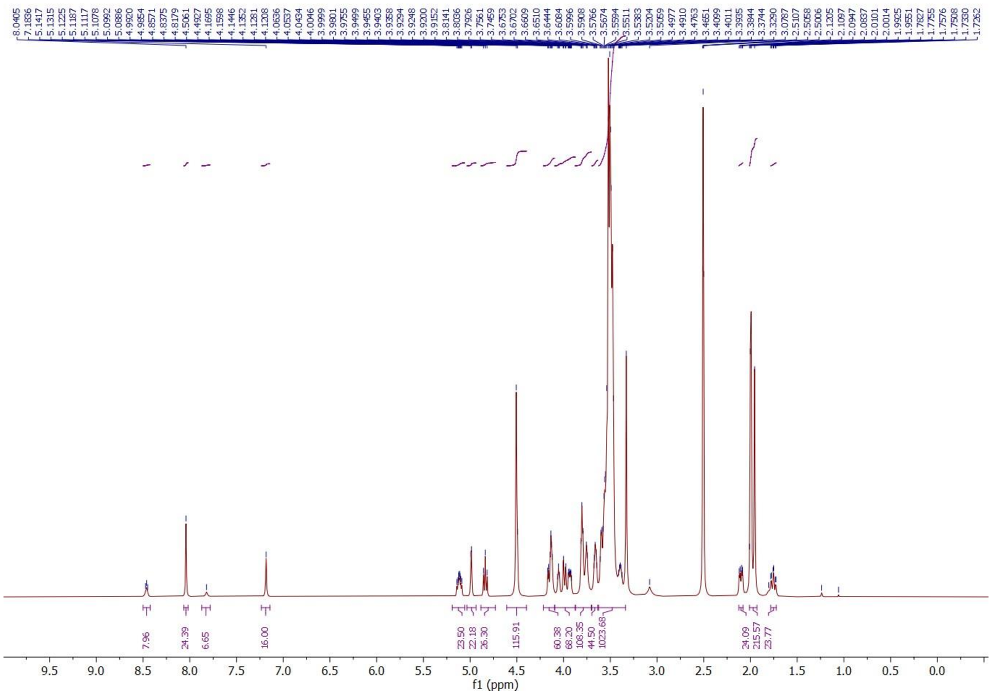

Figure S20: ^{1}H NMR of compound **15** in DMSO-d_6 (500 MHz).

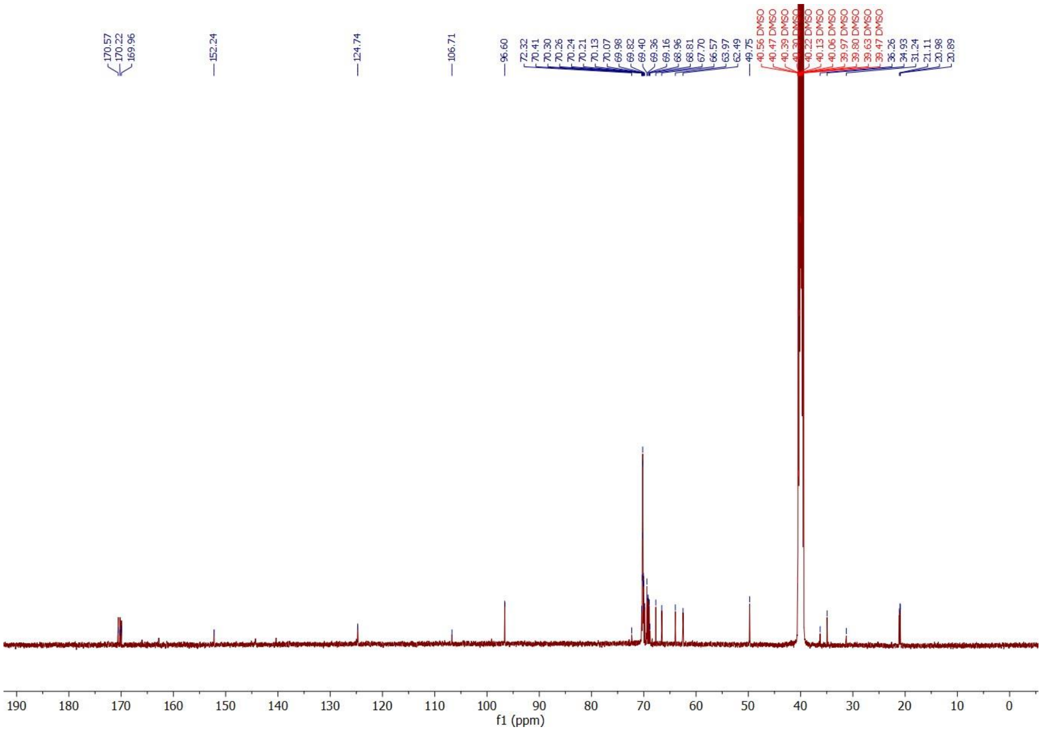

Figure S21: ^{13}C NMR of compound **15** in DMSO-d_6 (125 MHz).

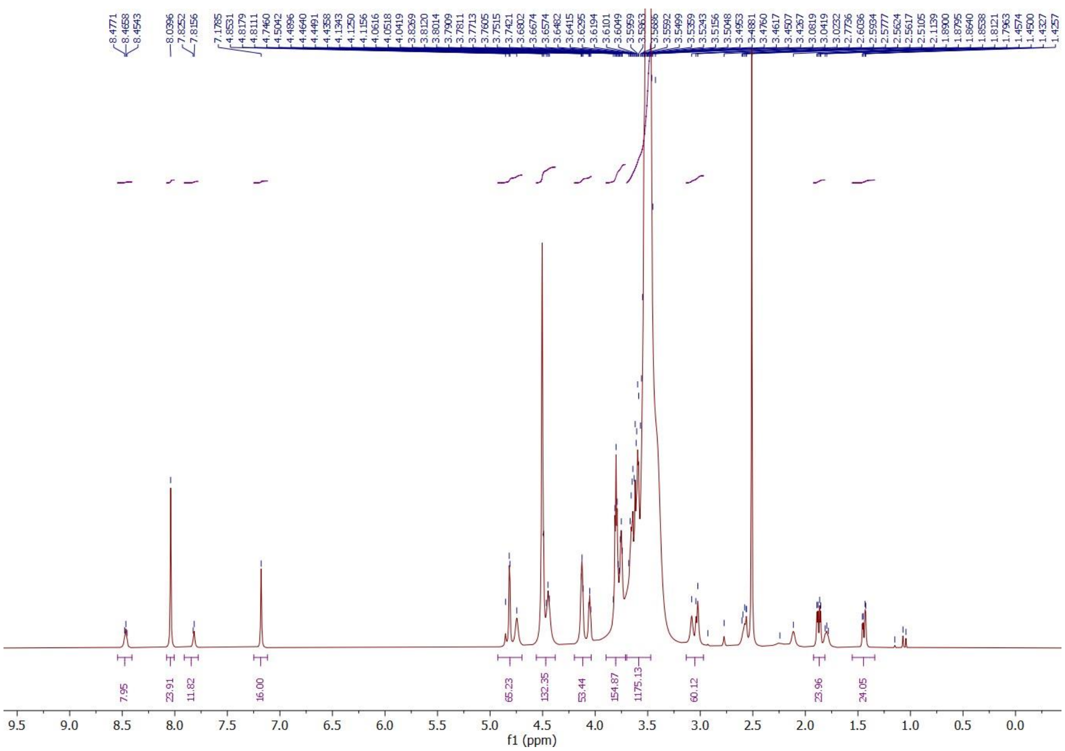

Figure S22: ^{1}H NMR of compound **16** in DMSO-d_6 (500 MHz).

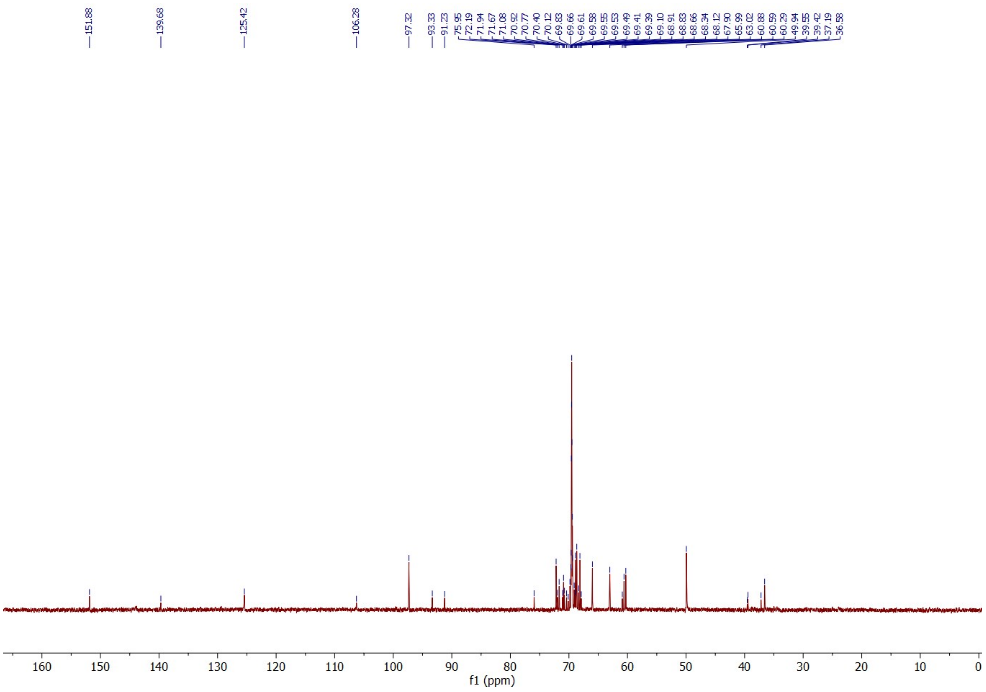

Figure S23: ^{13}C NMR of compound **16** in DMSO-d_6 (125 MHz).

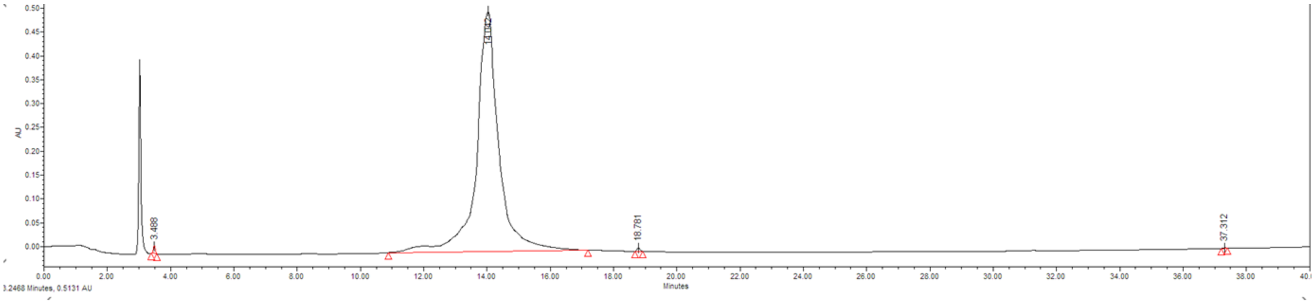

	Name	Retention Time (min)	Area (µV*sec)	% Area
1		3.488	74210	0.30
2		14.042	24804018	99.48
3		18.781	48575	0.19
4		37.312	7968	0.03

Figure S24: HPLC chromatogram dendrimer **16**.

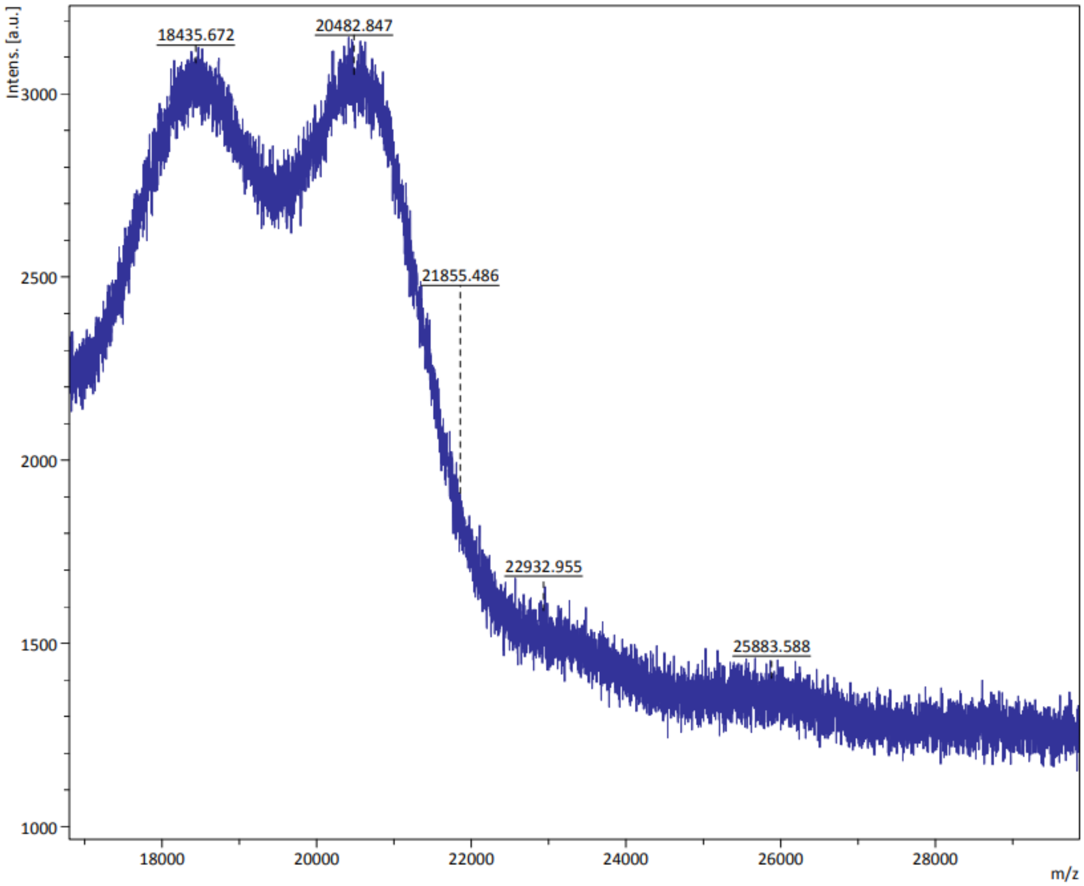

Figure S25: MALDI-ToF spectra dendrimer **16**.

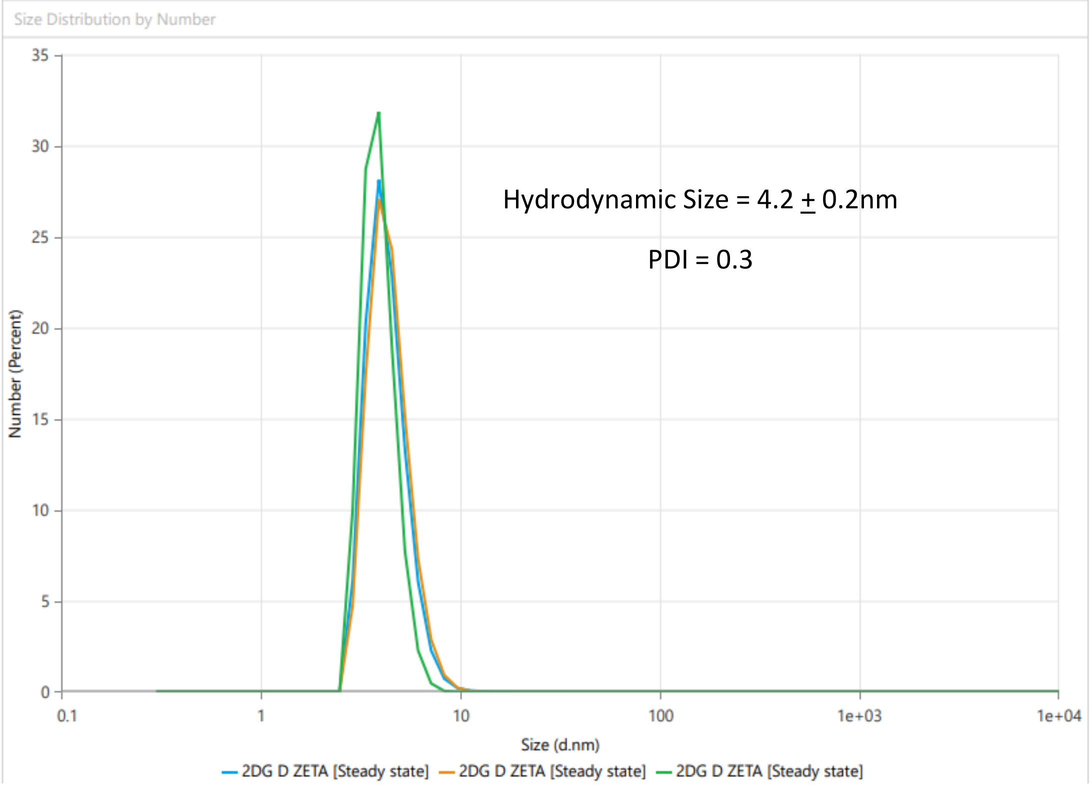

Figure S26: Size distribution of dendrimer **16** analyzed by DLS.

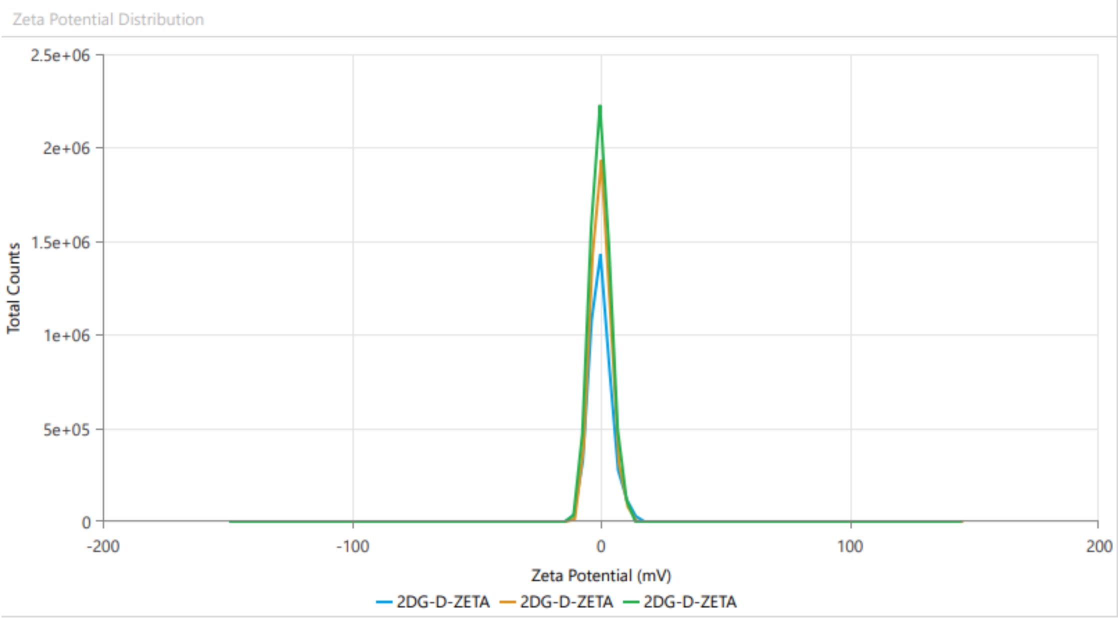

Figure S27: Zeta potential distribution of dendrimer **16**.

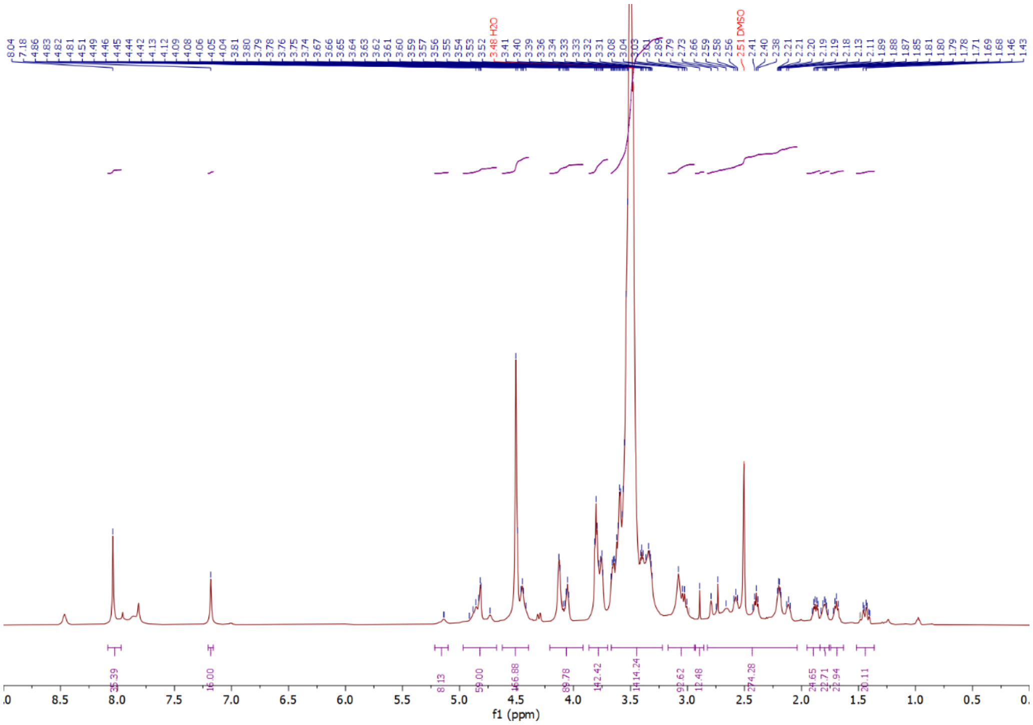

Figure S28: ^{1}H NMR of compound **17** in DMSO-d$_6$ (500 MHz).

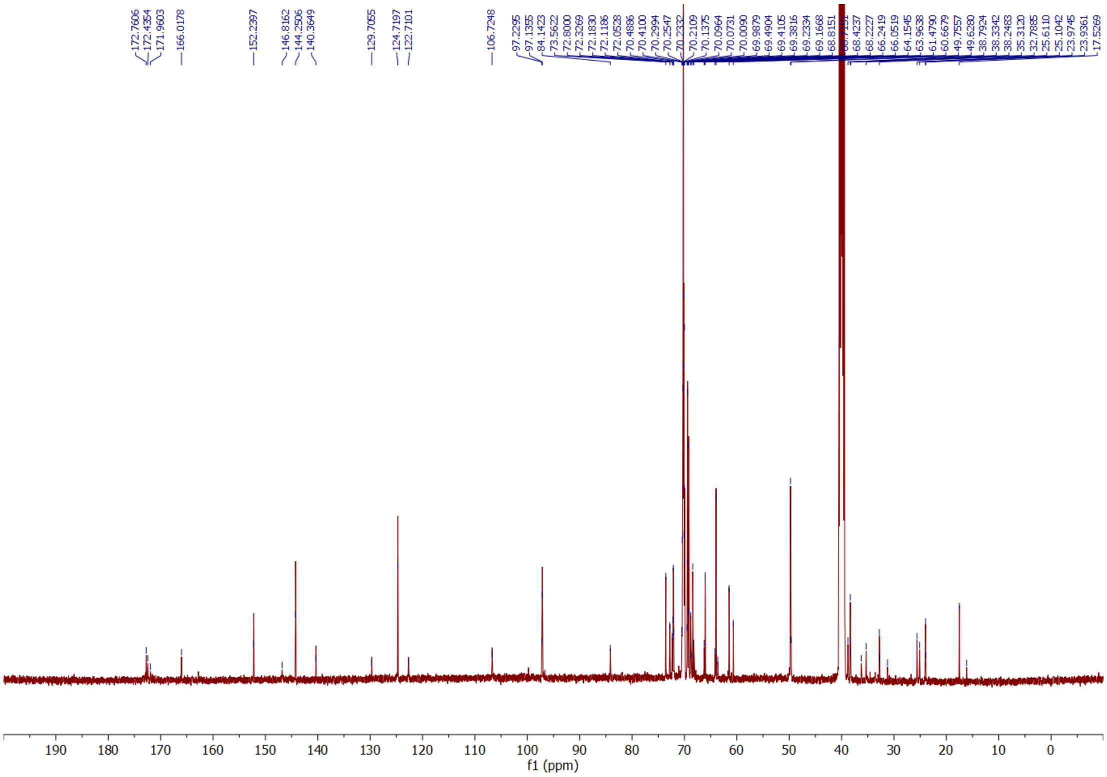

Figure S29: ^{13}C NMR of compound **17** in DMSO-d$_6$ (125 MHz).

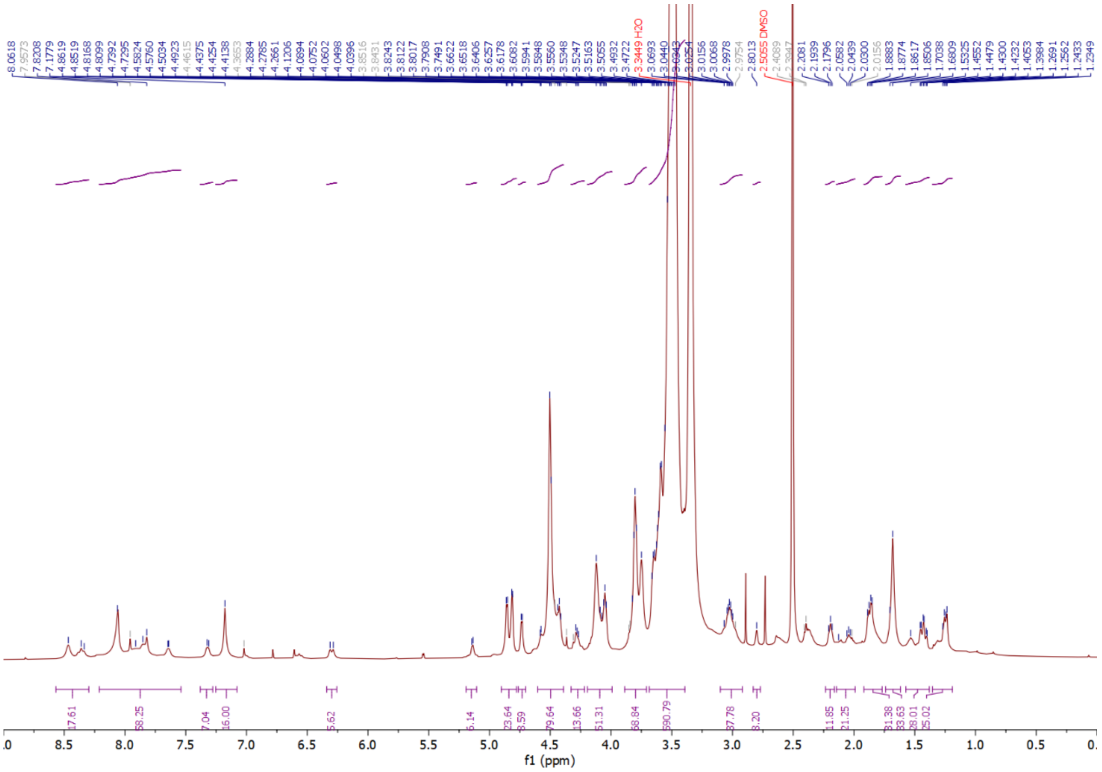

Figure S30: ¹H NMR of compound **18** in DMSO-d$_6$ (500 MHz).

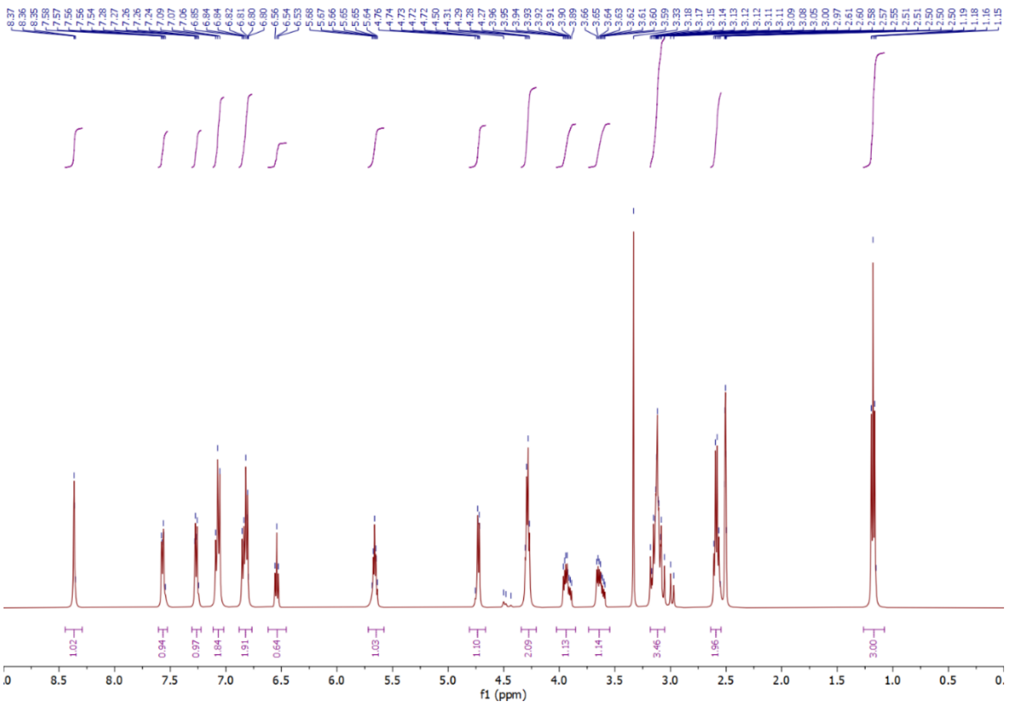

Figure S31: ¹H NMR of compound **20** in DMSO-d$_6$ (500 MHz).

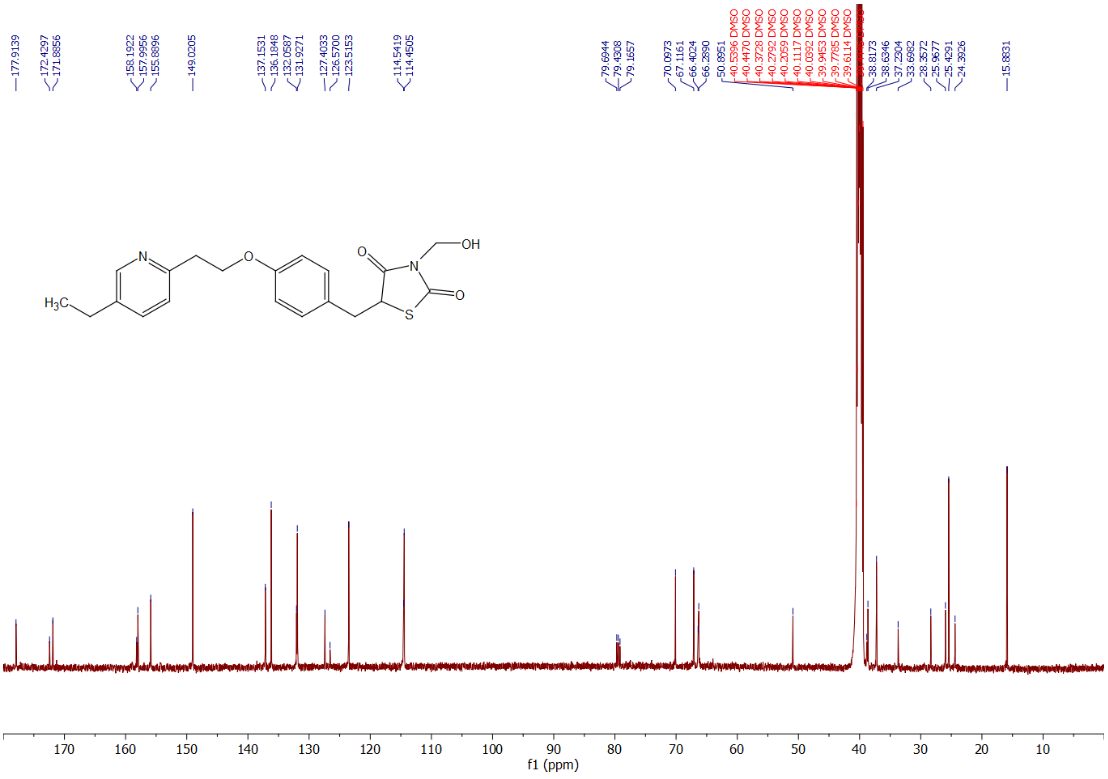

Figure S32: ^{13}C NMR of compound **20** in DMSO-d$_6$ (125 MHz).

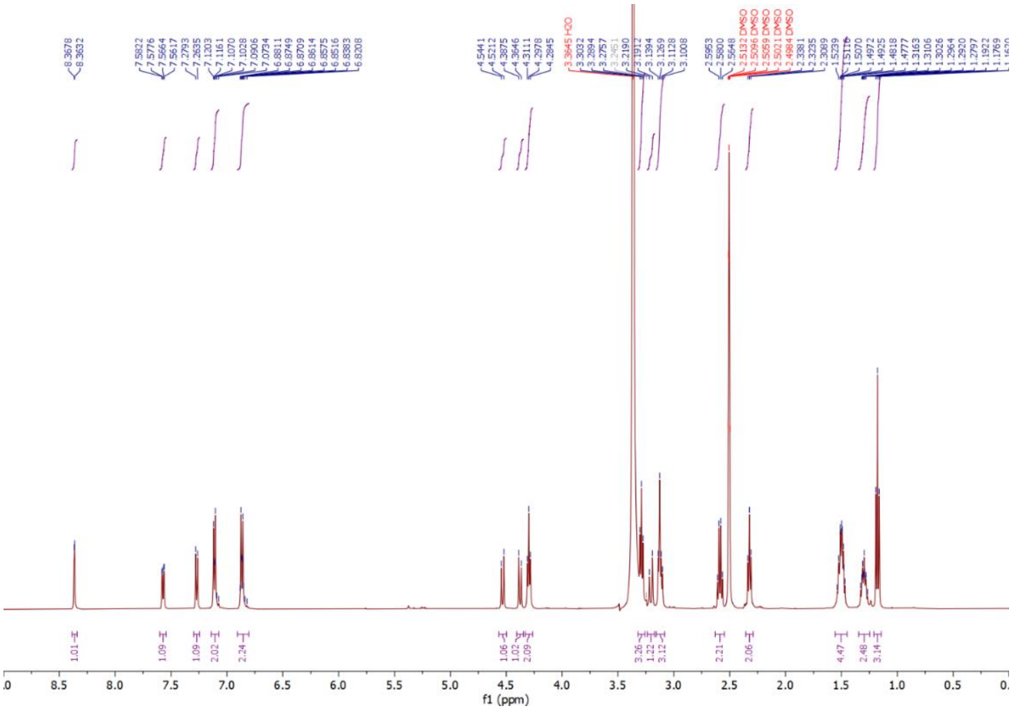

Figure S33: ^{1}H NMR of compound **21** in DMSO-d$_6$ (500 MHz).

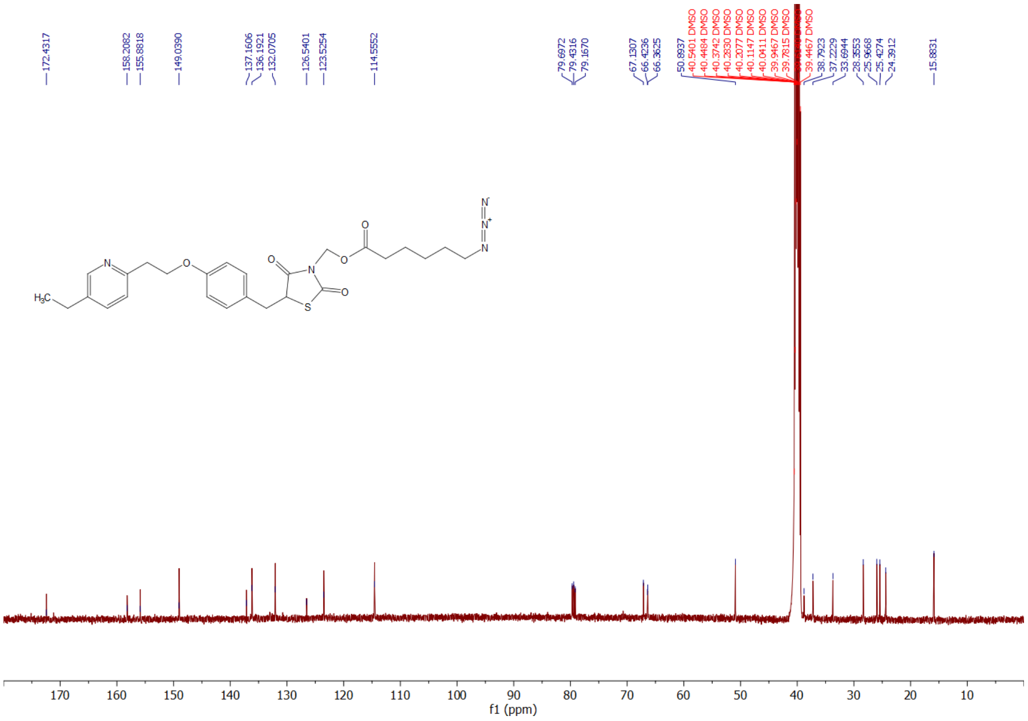

Figure S34: ^{13}C NMR of compound **21** in DMSO-d$_6$ (125 MHz)

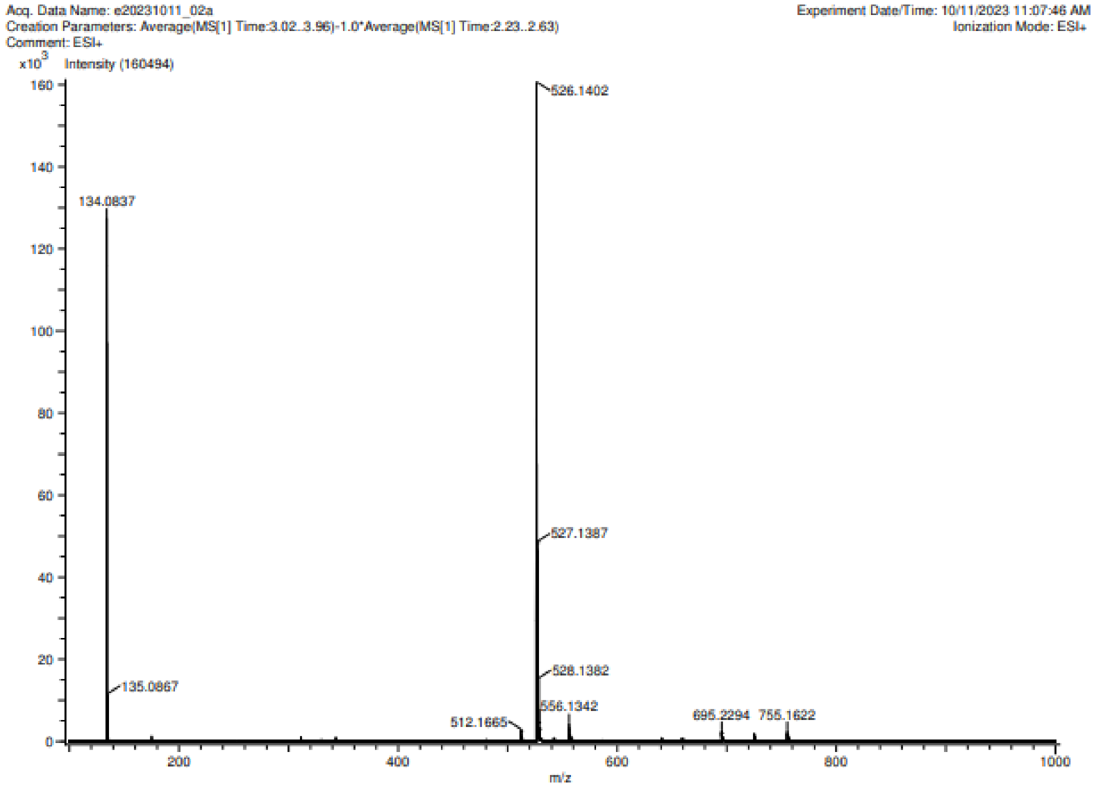

Figure S35: ESI mass spectra dendrimer **21**.

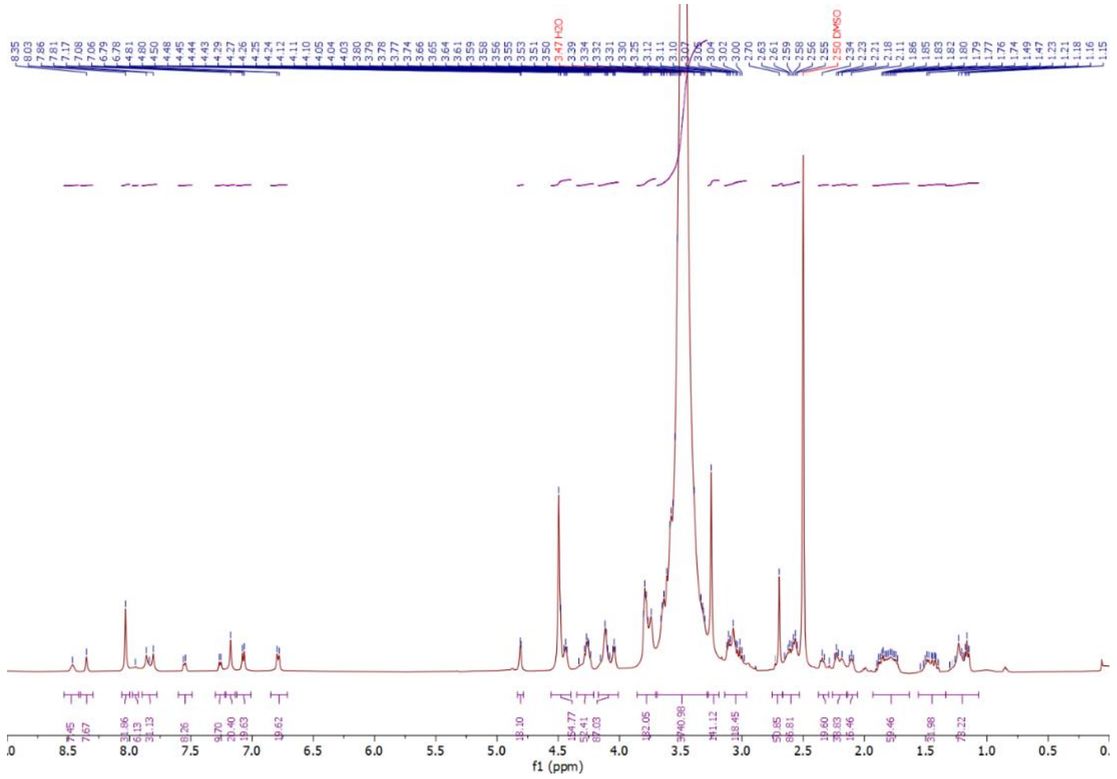

Figure S36: ^{1}H NMR of compound **22** in DMSO-d_6 (500 MHz).

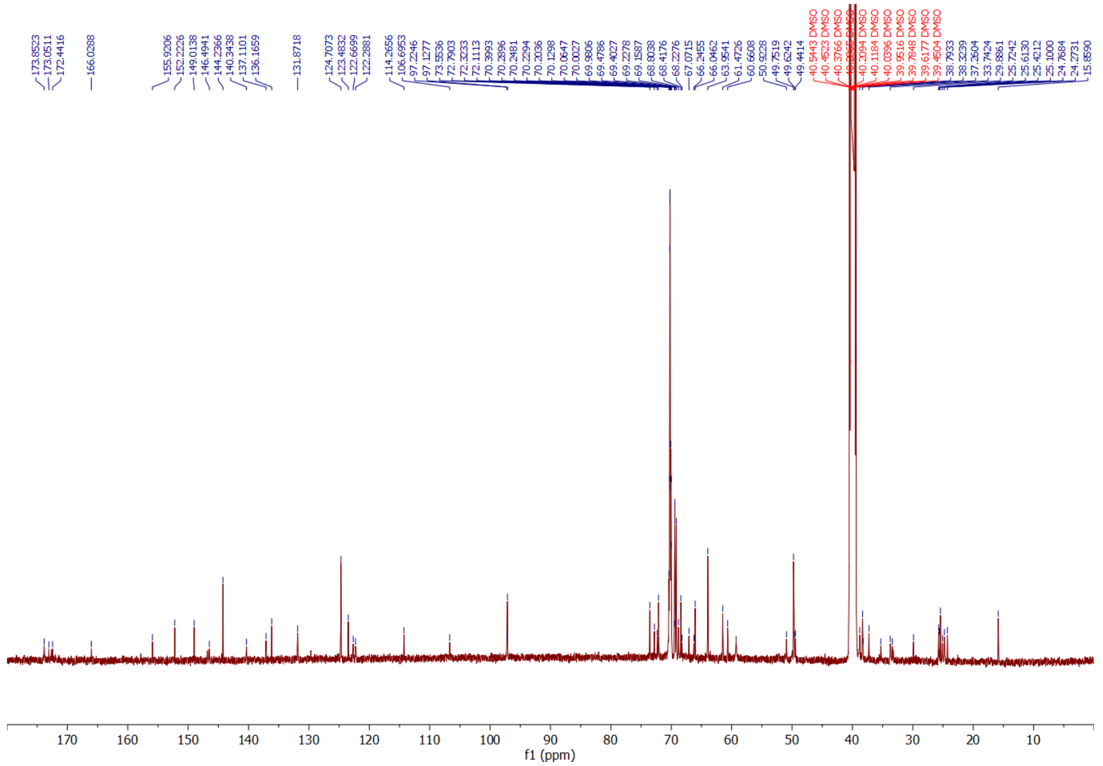

Figure S37: ^{13}C NMR of compound **22** in DMSO-d_6 (125 MHz).

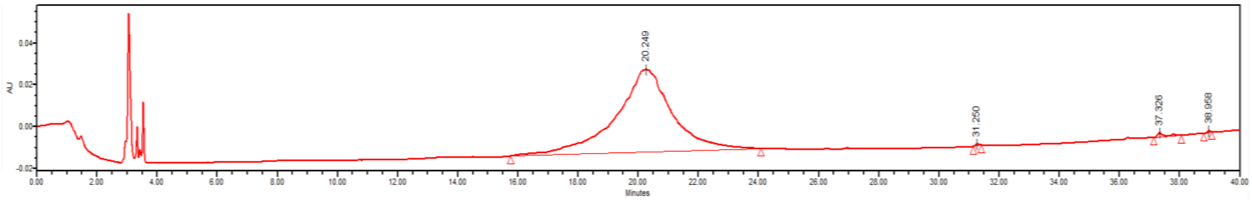

Retention Time (min)	Area (µV*sec)	% Area
20.249	5185236	99.14
31.255	17667	0.34
37.322	18643	0.36
38.962	8487	0.16

Figure S38: HPLC chromatogram of compound **22**.

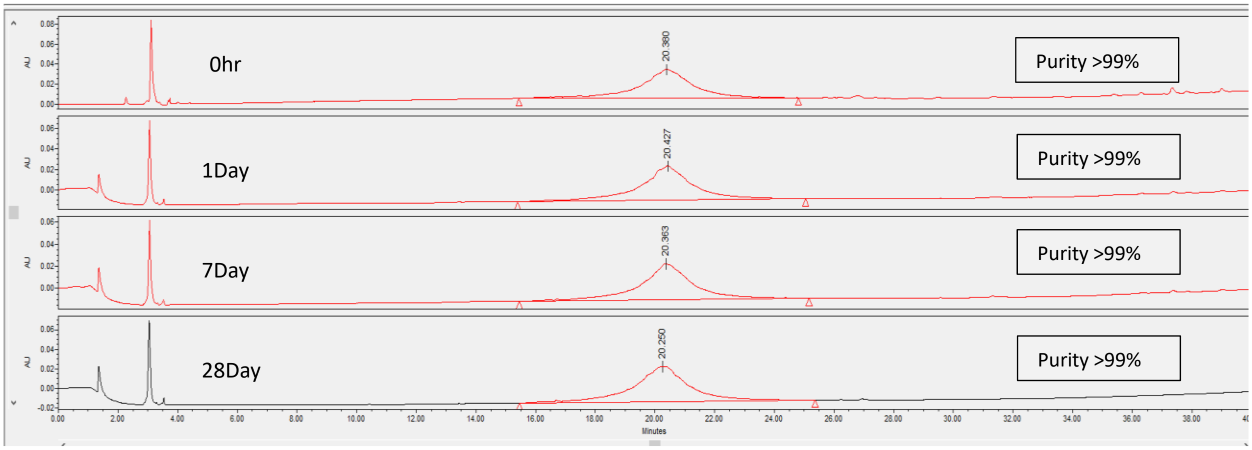

Figure S39: Formulation of *2DG-D-Pio* in PBS was found to be stable at RT until 28 Days as shown by the HPLC chromatogram @269nm at various time points.

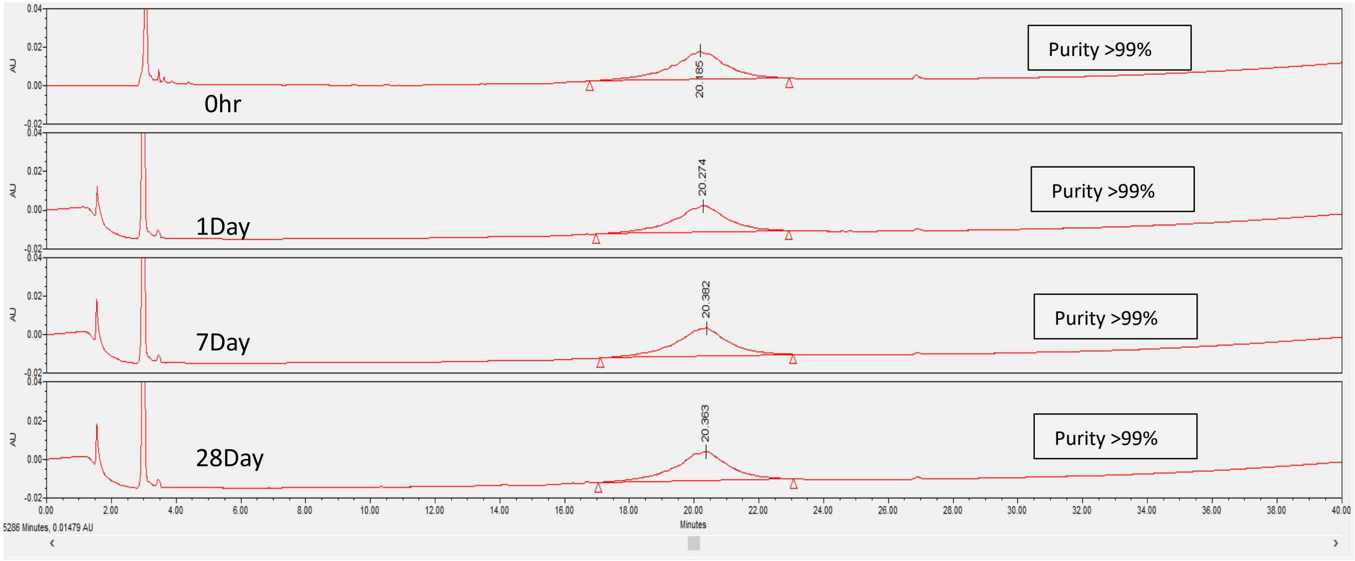

Figure S40: Formulation of *2DG-D-Pio* in PBS was found to be stable at 4°C until 28 Days as shown by the HPLC chromatogram @269nm at various time points.

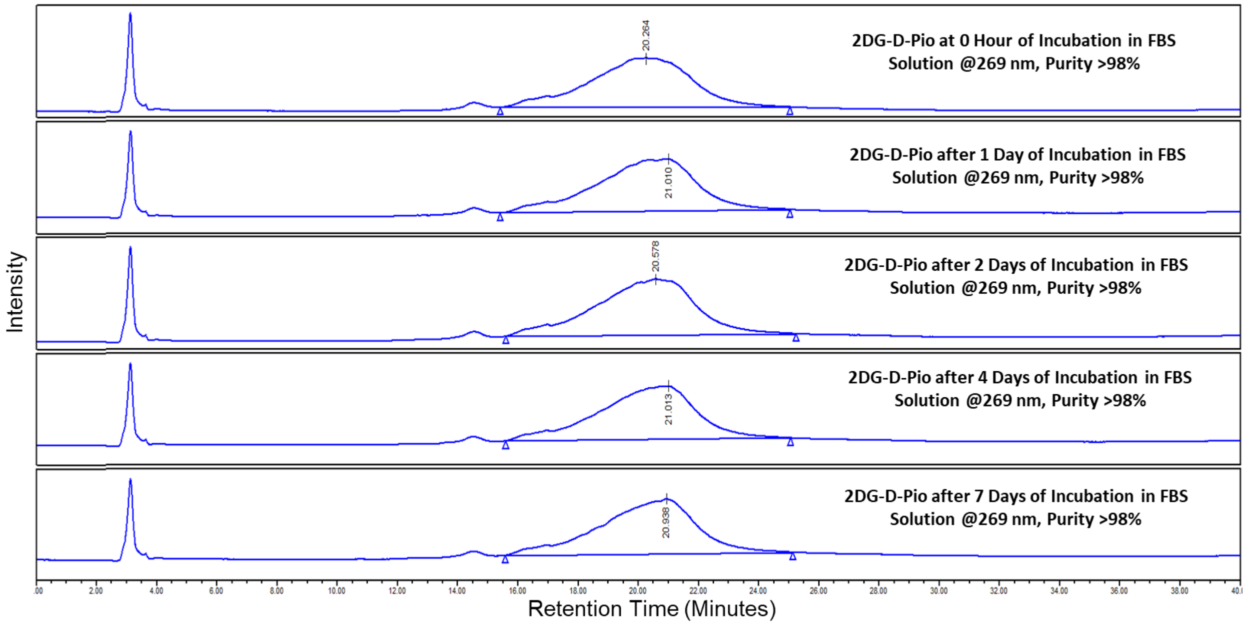

Figure S41: *2DG-D-Pio* in 2% FBS in PBS was found to be stable at physiological temperature until 7 Days as shown by the HPLC chromatogram @269nm at various time points.

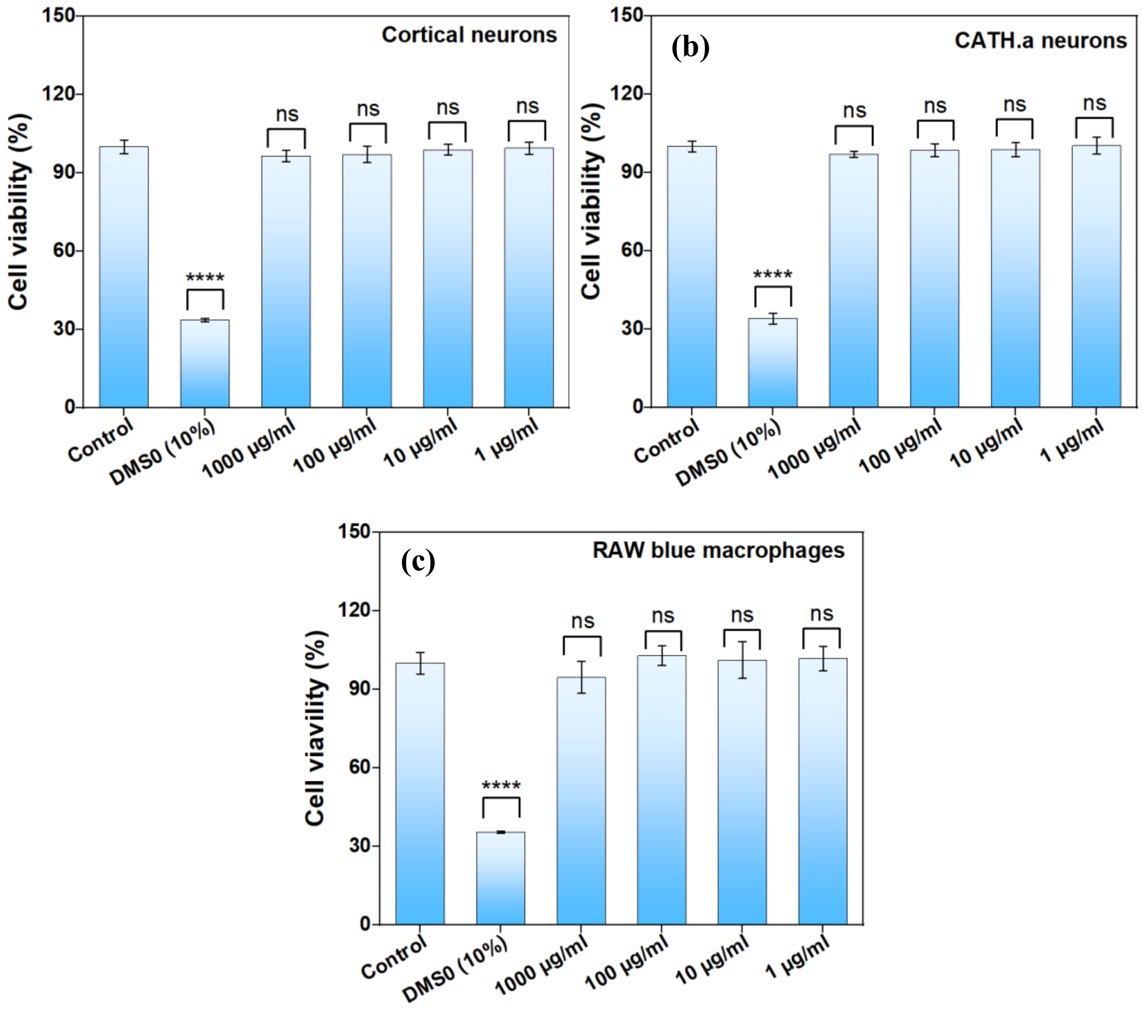

Figure S42: Cytocompatibility of (a) cortical neurons, (b) CATH.a neurons and (c) RAW blue macrophages treated with *2DG-D* dendrimer at different concentrations. The *2DG-D* dendrimer was found to be nontoxic to all the three cell types as determined by Two-way ANOVA (Turkey's multiple comparisons test), with no significant effect of treatments at all the concentrations. For this, the p-values were calculated between control, DMSO, and *2DG-D* treated cells, with **** $p < 0.0001$ and ns- non significant.

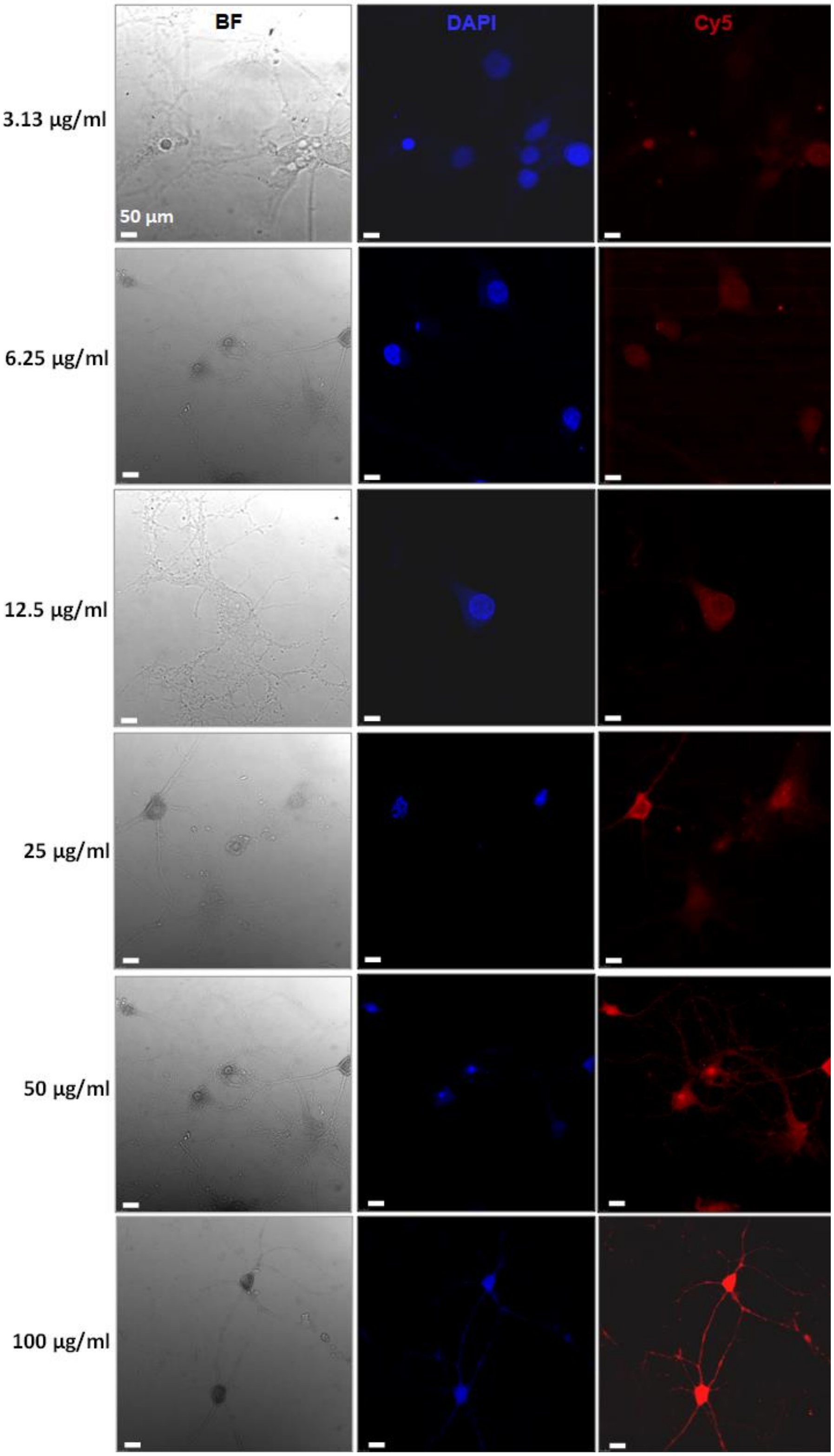

Figure S43. Confocal laser scanning micrographs showing concentration dependent uptake of the *2DG-D-Cy5* dendrimer by the cortical nuerons. The of scale bar shown in the figure is 50 µm.

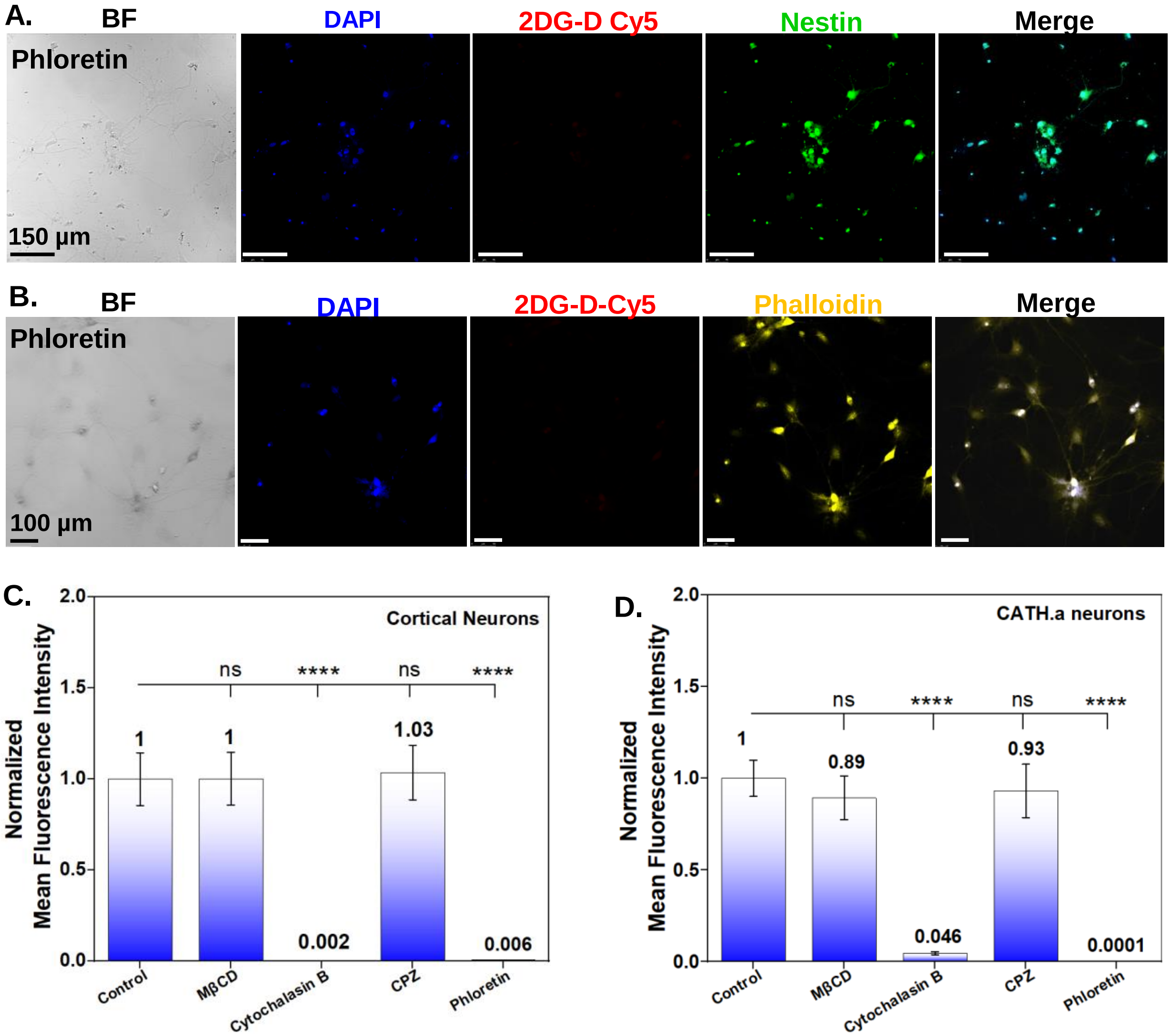

Figure S44. Confocal micrographs showing effect of Phloretin inhibitor on the uptake of the *2DG-D-Cy5* dendrimer on (a) cortical and (b) CATH.a mouse nuerons, showng no red fluorescence of Cy5 inside these neuronal cells. The cells stained with blue (DAPI), green (Nestin), yellow (Phalloidin) represent cell nucleus, nestin protein, and actin microflament proteins, respectively. The scale bar shown in the figure is 150 μm (a) and 100 μm (b). Images are representative for three independent experiments. Normalized mean fluorescence intensities (MFIs) of (c) cortical neurons and (d) CATH.a neurons showing uptake of *2DG-D-Cy5* in presence of trafficking inhibitors using ImageJ software. For this, the p-values were calculated between control (no inhibitor) with inhibitor treated cells, with **** $p < 0.0001$ and ns- non significant.

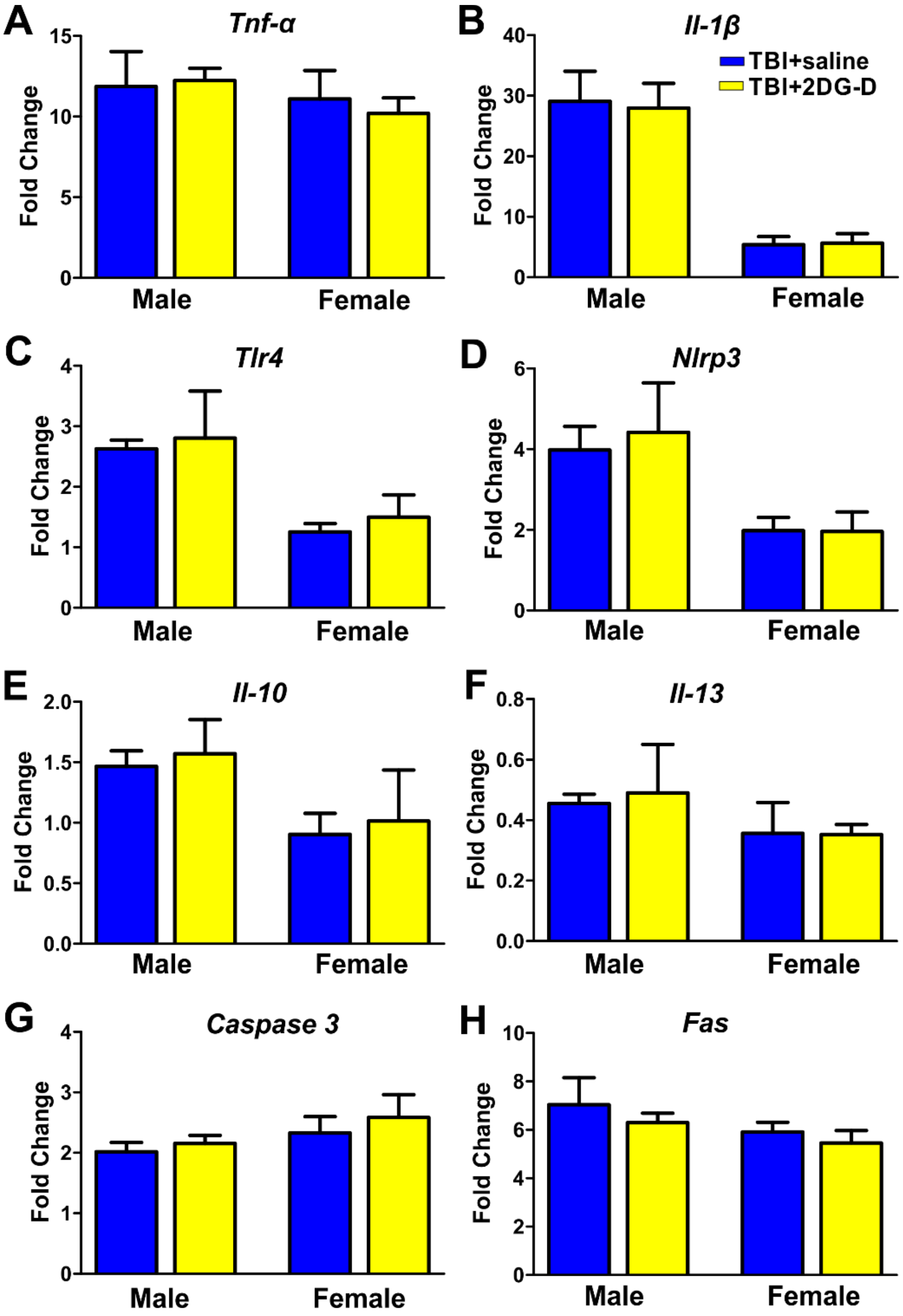

Figure S45. mRNA expressions of pro- and anti-inflammatory markers and cell death markers from TBI+saline (n=10, 5M/5F) and TBI+*2DG-D* (n=4, 2M/2F) groups. Neurons were isolated from the injured brain regions at 24-h post-treatment for gene expression evaluations. Data were presented as mean ±SEM. Treatment groups were presented as TBI+saline (blue) and TBI+*2DG-D* (yellow). **A-D,** The expression of pro-inflammatory makers, TNF-α (A), IL-1β (B), TLR4 (C), and NLRP3 (D). **E, F,** The expression of anti-inflammatory makers IL-10 (E) and IL-13 (F). **G, H,** The expression of cell death makers caspase-3 (G) and Fas (H).

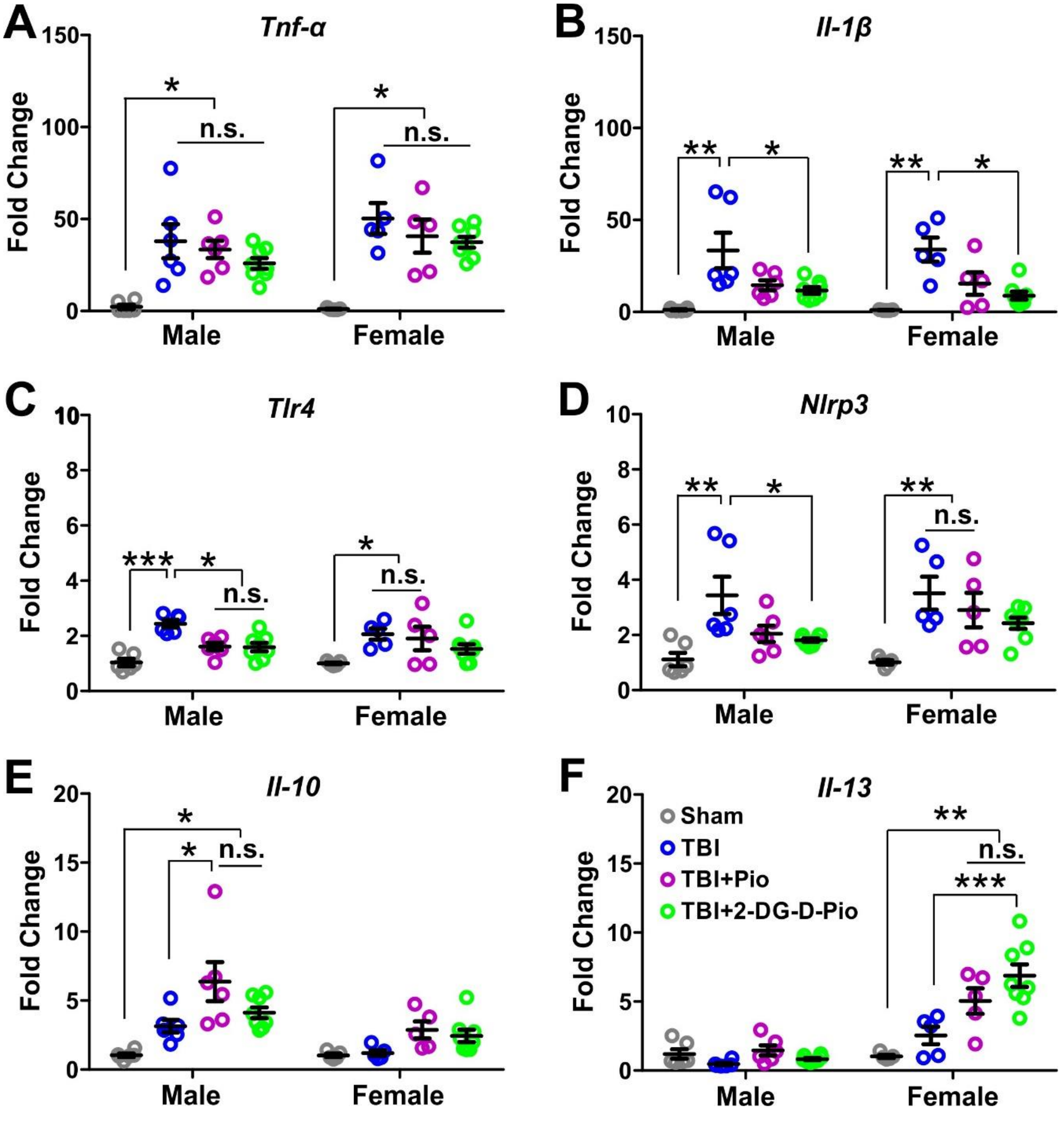

Figure S46. mRNA expressions of pro- and anti-inflammatory markers from sham (n=11, 6M/5F), TBI+saline (n=11, 6M/5F), TBI+*Pio* (n=11, 6M/5F), and TBI+*2DG-D-Pio* (n=16, 8M/8F) groups. The brain tissues from the injured area (or the matching area from the sham mice) were harvested at 24-h post-treatment for gene expression evaluations. **A,** TNF-α significantly increased in both male and female TBI+saline, TBI+*Pio*, and TBI+*2DG-D-Pio* groups, compared with the sham groups. **B,** IL-1β significantly increased in both male and female TBI+saline groups, compared with the male and female sham and TBI+*2DG-D-Pio* groups. **C,** TLR4 significantly increased in

male TBI+saline group, compared with the male sham, TBI+*Pio*, and TBI+*2DG-D-Pio* groups. TLR4 significantly increased in female TBI+saline and TBI+*Pio* groups, compared with the female sham group. **D,** NLRP3 significantly increased in male TBI+saline group, compared with the male sham and TBI+*2DG-D-Pio* groups. NLRP3 significantly increased in female TBI+saline and TBI+*Pio* groups, compared with the female sham group. **E,** IL-10 significantly increased in both male TBI+*Pio* and TBI+*2DG-D-Pio* groups, compared with the male sham group. In addition, IL-10 significantly increased in the male TBI+*Pio* group, compared with the male TBI+saline group. **F,** IL-13 significantly increased in both female TBI+*Pio* and TBI+*2DG-D-Pio* groups, compared with the female sham group. In addition, IL-13 significantly increased in female TBI+ *2DG-D-Pio* group, compared with the female TBI+saline group. *, $p<0.05$; **, $p<0.01$; ***, $p<0.001$; n.s., no significance.
